# Comparative Transcriptome Analysis of Iron and Zinc Deficiency in Maize (*Zea mays* L.)

**DOI:** 10.3390/plants9121812

**Published:** 2020-12-21

**Authors:** Mallana Gowdra Mallikarjuna, Nepolean Thirunavukkarasu, Rinku Sharma, Kaliyugam Shiriga, Firoz Hossain, Jayant S Bhat, Amitha CR Mithra, Soma Sunder Marla, Kanchikeri Math Manjaiah, AR Rao, Hari Shanker Gupta

**Affiliations:** 1 Maize Research Lab, Division of Genetics, ICAR-Indian Agricultural Research Institute, New Delhi 110012, India; tnepolean@gmail.com (N.T.); rinkusharma882012@gmail.com (R.S.); kaliyugs@gmail.com (K.S.); fh_gpb@yahoo.com (F.H.); 2 IARI-Regional Research Centre, Dharwad, Karnataka 580005, India; jsbhat73@gmail.com; 3 ICAR-National Institute for Plant Biotechnology, New Delhi 110012, India; amithamithra.nrcpb@gmail.com; 4 ICAR-National Bureau of Plant Genetic Resources, New Delhi 110012, India; ssmarl@yahoo.com; 5 Division of Soil Science and Agricultural Chemistry, ICAR-Indian Agricultural Research Institute, New Delhi 110012, India; manjaiah.math@gmail.com; 6 ICAR-Indian Agricultural Statistics Research Institute, New Delhi 110012, India; ar.rao@icar.gov.in

**Keywords:** functional genomics, homeostasis, hormonal regulation, iron, maize, malnutrition, photosynthesis, zinc

## Abstract

Globally, one-third of the population is affected by iron (Fe) and zinc (Zn) deficiency, which is severe in developing and underdeveloped countries where cereal-based diets predominate. The genetic biofortification approach is the most sustainable and one of the cost-effective ways to address Fe and Zn malnutrition. Maize is a major source of nutrition in sub-Saharan Africa, South Asia and Latin America. Understanding systems’ biology and the identification of genes involved in Fe and Zn homeostasis facilitate the development of Fe- and Zn-enriched maize. We conducted a genome-wide transcriptome assay in maize inbred SKV616, under –Zn, –Fe and –Fe–Zn stresses. The results revealed the differential expression of several genes related to the mugineic acid pathway, metal transporters, photosynthesis, phytohormone and carbohydrate metabolism. We report here Fe and Zn deficiency-mediated changes in the transcriptome, root length, stomatal conductance, transpiration rate and reduced rate of photosynthesis. Furthermore, the presence of multiple regulatory elements and/or the co-factor nature of Fe and Zn in enzymes indicate their association with the differential expression and opposite regulation of several key gene(s). The differentially expressed candidate genes in the present investigation would help in breeding for Fe and Zn efficient and kernel Fe- and Zn-rich maize cultivars through gene editing, transgenics and molecular breeding.

## 1. Introduction

Iron (Fe) and zinc (Zn) are essential elements for all living organisms, including plants and animals. Fe and Zn also act as co-factors of numerous enzymes and in turn play vital roles in various physiological process, *viz*., photosynthesis, respiration, electron transport, protein metabolism, chlorophyll synthesis and hormonal regulations [1,2]. Therefore, any deficiency in Fe and Zn affects the economic yield in crops and thereafter manifests in the form of micronutrient malnutrition in humans [3]. The development of Fe- and Zn-efficient cultivars is one of the effective approaches to sustain crop production and to alleviate widespread micronutrient-malnutrition. Hence, understanding the functional genomics of Fe and Zn homeostasis and identification of target genes and pathways in major staple crops will help in the genetic biofortification of crop plants for Fe and Zn.

Plants adapt a complex network of homeostatic mechanisms to regulate Fe and Zn uptake, transport and accumulation [4]. The uptake of Fe ions in plants occurs through two important strategies *viz*., the reduction-based strategy (strategy-I) and chelation-based strategy (strategy-II). The strategy-I is present in all the plants except those from the Poaceae family. Under Fe deficiency, the strategy-I plants release the protons into the rhizosphere by H^+^-ATPases and makes the Fe more soluble by lowering the soil pH. Subsequently, the NADPH-dependent ferric chelate reductase (FRO2), reduces Fe^3+^ to Fe^2+^ form and which can then be transported into the root epidermis by the divalent metal transporter, iron regulated transporter 1 (IRT1). On the other hand, plants of Poaceae family mostly follow a mugineic acid (MA) pathway-based chelation strategy (Strategy-II) to uptake the Fe from soil [5]. It has been reported that in the mugineic acid pathway, nicotianamine synthase (NAS) [6], nicotianamine aminotranferase (NAAT) [7] and deoxymugineic acid synthase (DMAS) [8] mediate the synthesis of deoxymugineic acid (DMA) from S-adenosyl-methionine via a series of reactions [9]. The grass plants release derivatives of deoxymugineic acid (DMA) called phytosiderophores (PS) which make the complex with the ferric ions (Fe^3+^). The efflux of PS is facilitated by transporters of the mugineic acid family phytosiderophores (TOM1 and TOM2) whereas the influx of PS–Fe^3+^ is mediated through yellow stripe like-1 (YS1) transporters [10,11]. Among Poaceae members, rice possesses both strategy I and II for the uptake of Fe [2,3,4]. Interestingly, in maize, the recent finding showed the presence of strategy-I genes for the uptake of Fe [12].

Plants absorb the Zn, mainly in divalent cationic form (Zn^2+^) [13]. The Zn uptake mechanisms in plants are equipped with a dual-transporter system, *viz*., high-affinity transport system (HATS) and low-affinity transport system (LATS) [14,15,16]. The LATS mechanism operates when the Zn is in high concentration and the HATS operates when the Zn concentration is low [16]. Several transporters *viz*., ZRT-IRT-like protein (ZIP) family [17], HMA (heavy metal ATPase) family [18], MTP (metal tolerance protein) family [19], vacuolar iron transporter (VIT) family, and plant cadmium resistance family (PCR) proteins mediate the Zn transport across the root plasma membrane into root cells [14,20]. Additionally, in plants many researchers also reported the chelation-based Zn uptake mechanism [20,21].

Various transporters and chelation agents *viz*., oligopeptide transporters (OPT) and yellow stripe like (YSL) [22,23], ZIP [24,25,26,27], ferric reductase defective protein (FRD) transporters [28], DMA [29], nicotianamine (NA) [22,30], and citrate [31] have been reported to be involved in the mobilization of Fe and Zn ions. Furthermore, genome-wide transcriptome assays have been employed to understand the expression pattern of genes and pathways associated with Fe and Zn deficiencies in rice [32], *Arabidopsis* [33], maize [34], and several other crops [35]. The deficiency of Fe and Zn is known to activate distinct functional gene modules such as the ‘transportome’ which, encompasses genes encoding metal transporters, root system modifications, primary metabolic pathways and hormonal metabolism [33]. Under Fe deficiency, Li et al. [34] reported the induced regulation of genes associated with plant hormones, protein kinases and phosphatase in maize roots; whereas, Zanin et al. [36] showed the presence of strategy-I components, which is most prominent in dicot plant. Like Fe, Zn deficiency results in the higher expression of *ZIP* and *NAS* genes in maize [37]. The chlorophyll biosynthesis and rate of photosynthesis are severely affected through the altered expression of genes associated with chloroplast biosynthesis and photosynthesis under Fe and Zn deficiency [9,32,38]. Studies by Garnica et al. [38] in wheat and García et al. [39] in *Arabidopsis* revealed the coordinated action of indole acetic acid (IAA), ethylene and nitrate oxide (NO) in the signalling networks of Fe deficiency. Phytohormones *viz*., abscisic acid (ABA), and salicylic acid (SA) regulate the expression pattern of *ZmNAS* in maize seedlings [40]. Similarly, ethylene increases the transcripts abundance of genes *viz*., *bHLH* (*BASIC HELIX-LOOP-HELIX*), *IRO2* (*IRON-RELATED TRANSCRIPTION FACTOR 2*), *NAS1* (*NICOTIANAMINE SYNTHASE 1*), *NAS2* (*NICOTIANAMINE SYNTHASE 2*), *YSL15* (*YELLOW STRIPE LIKE 15*) and *IRT1* (*IRON REGULATED TRANSPORTER 1*) associated with Fe^2+^ and Fe^3+^-phytosiderophore uptake systems in rice [41]. In *Arabidopsis*, NO enhances the expression of genes for Fe-acquisition and ethylene synthesis in roots [42,43] and the interaction between the auxin and NO modulates the root growth under Fe deficiency in rice [44]. Apart from root growth, Fe deficiency induced auxin signalling, which results in photosynthesis inhibition and defective shoot growth in rice seedlings [45].

The comparative analysis of nutrients and their interactions are crucial for the simultaneous improvement of multi-nutrient use efficiency and enrichment in crops. Few of the interaction studies at whole-genome transcriptome strata are available in crops for Fe and P [32] and Zn and P [46]. However, there are hardly any reports on the genome-wide expression studies under Fe and Zn deficiency interactions, in both root and shoot tissues of crops in general and maize in specific. Therefore, the present investigation was designed to study the transcriptome response to Fe, and Zn deficiencies individually and together to understand Fe and Zn homeostasis and metabolism in maize.

## 2. Results

### 2.1. Morpho-Physiological Evidence for Fe and Zn Interaction

**Figure 1:**
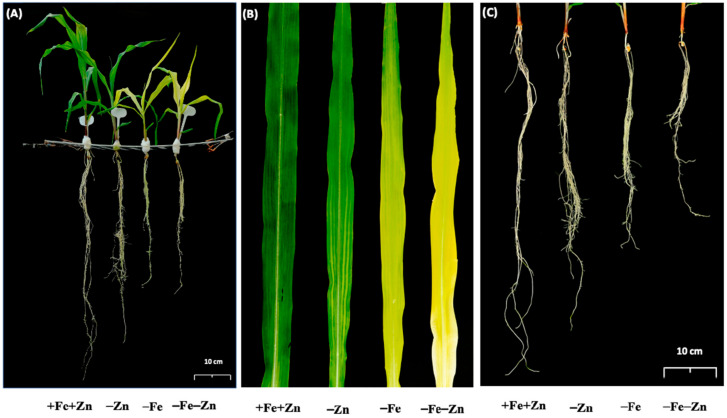
Phenotypic expression of 19 days-old maize SKV616 seedlings in response to 12 days exposure to Zn and Fe deficiencies (–Zn, –Fe and –Fe–Zn): (**A**) whole plant; (**B**) leaves; and (**C**) roots.

Maize seedlings when tested under Fe and Zn deficiencies individually and together (–Zn, –Fe and –Fe–Zn) along with control (+Fe+Zn). The stress treatments started showing Fe and Zn deficiency symptoms from the fourth and fifth days after transplanting (DAT), respectively. However, the typical stress symptoms were prominent among the stress treatments at 10 DAT (Figure 1). Seedlings showed chlorosis under Fe deficiency (–Fe), interveinal chlorosis in the lower half of the top leaves under Zn deficiency (–Zn), and severe chlorosis coupled with slight whitish blotches near the base of top leaves under combined Fe and Zn deficiencies (–Fe–Zn). Furthermore, reduced root length and induced root hair formation were observed under stress treatments (Figure 1).

**Figure 2:**
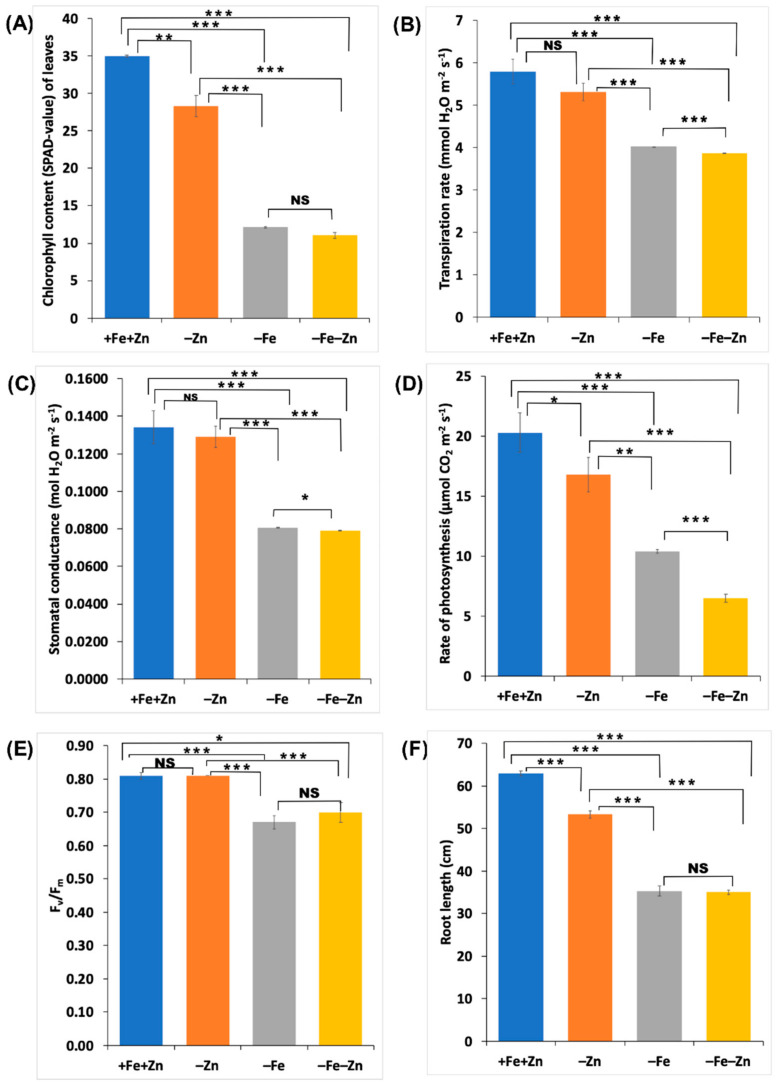
The morpho-physiological response of maize seedlings to –Zn, –Fe and –Fe–Zn deficiencies at 12 days after transplanting (DAT): (**A**) change in the chlorophyll content (soil plant analysis development (SPAD) value) of the leaves in response to the (**B**) transpiration rate; (**C**) stomatal conductance; (**D**) photosynthesis rate; (**E**) quantum efficiency of PS II photochemistry (Fv/Fm); and (**F**) root length (*, **, *** significant at *p* < 0.05, *p* < 0.01 and *p* < 0.001, respectively; NS: non-significant).

Plants in Fe (–Fe), and Fe and Zn (–Fe–Zn) deficiencies started showing chlorosis from the fourth DAT; in contrast, the chlorophyll content of leaves increased significantly in the seedlings of the control treatment (+Fe+Zn) (Figure S1). However, no significant variation in the chlorophyll content was observed between –Fe and –Fe–Zn deficiencies (Figure 2A). Zn starved the seedlings in comparison to the control which did not show a significant reduction in stomatal conductance and transpiration rate. In contrast, as compared to the control and Zn deficiency (–Zn), the Fe (–Fe) and Fe and Zn (–Fe–Zn) deficiencies recorded a significantly reduced transpiration rate and stomatal conductance (Figure 2B,C).

Additionally, the reduced rate of photosynthesis was observed in all the stress treatments as compared to the control (Figure 2D). The –Fe and –Fe–Zn stresses showed a significant reduction in the maximum photochemical efficiency of PSII (F_V_/F_M_), whereas no significant decrease was observed under –Zn stress (Figure 2E). Furthermore, –Fe and –Fe–Zn stressed seedlings showed a similar level of photochemical efficiency of PSII (Figure 2E).

Roots are the absorption points for nutrients and showed variation in the root length under nutrient stresses. The reduction in root lengths was significant for all the stress treatments. However, –Fe and –Fe–Zn treatments did not show any difference in root length. Therefore, Fe deficiency (–Fe and –Fe–Zn) is the major contributor for root length reduction as compared to Zn deficiency (–Zn) (Figure 2F). All the morpho-physiological traits showed a high degree of correlation (*p* < 0.01 and *p* < 0.001) owing to interdependency on each other at physiological level. The stomatal conductance and transpiration rate, and chlorophyll content and root length showed the maximum correlation coefficients (r = 0.99, *p* < 0.001) (Figure S2).

### 2.2. Maize Transcriptome Profiles in Response to Fe and Zn Deficiencies

**Figure 3:**
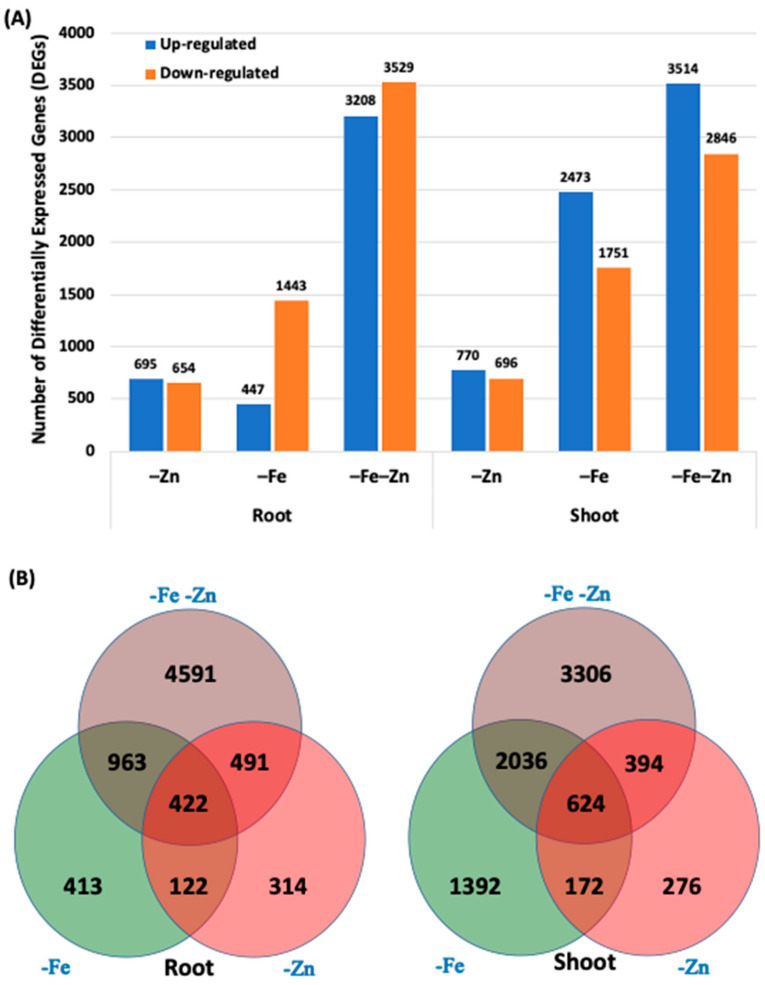
The overview of the spatial transcriptome responses to Fe and Zn stresses in the maize inbred line of SKV616 seedlings. The genes showing > 2-fold expression under stress treatments and *p* < 0.05 are considered as differentially expressed genes (DEGs) in stress treatments as compared to the control: (**A**) total number of upregulated and downregulated genes in response to –Zn, –Fe and –Fe–Zn stresses in the root and shoot; and (**B**) the Venn diagram depicting the stress-specific and common DEGs in response to –Zn, –Fe and –Zn–Fe stresses in the root and shoot tissues.

Root and shoot tissues collected from hydroponically grown maize seedlings under treatments and control were analysed for a genome-wide transcriptome assay using the Affymetrix GeneChip maize genome arrays (Affymetrix Inc., Santa Clara, California, USA). The transcriptome snapshots of stress treatments were compared against control seedlings. Genome-wide transcriptome analysis under –Zn, –Fe and –Fe–Zn stresses identified differential expression of 1349, 1920 and 6467 genes, respectively, in root and 1466, 4224 and 6360 genes in the shoot, respectively (Figure 3). In both the root and shoot, –Fe–Zn resulted in a higher number of differentially expressed genes (DEGs) as compared to individual stress (–Zn or –Fe). The roots of –Fe–Zn treatment showed a relatively higher number of DEGs (6467) as compared to the shoot (6360) (Figure 3). Conversely, the overall transcriptome snapshot showed a greater number of DEGs in the shoot (8200) as compared to the root (7316).

### 2.3. Gene Ontology Assignments and KEGG Enrichment Analyses

**Figure 4:**
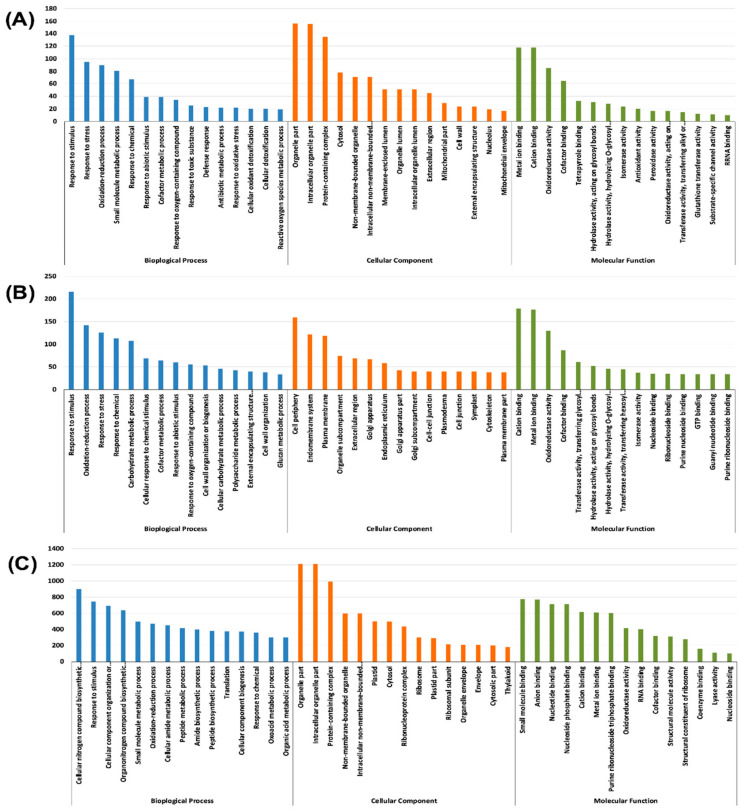
Functional gene ontology annotations of differentially expressed genes (DEGs) under Fe and Zn stresses in the root: (**A**) –Zn; (**B**) –Fe; and (**C**) –Fe–Zn. For graphical representation, we have the top 15 significant terms under each category *viz*., biological process (blue), cellular component (orange), and molecular function (green). The complete list of terms along with significance of false discovery rate (FDR) < 0.05 are mentioned in Table S1.

**Figure 5:**
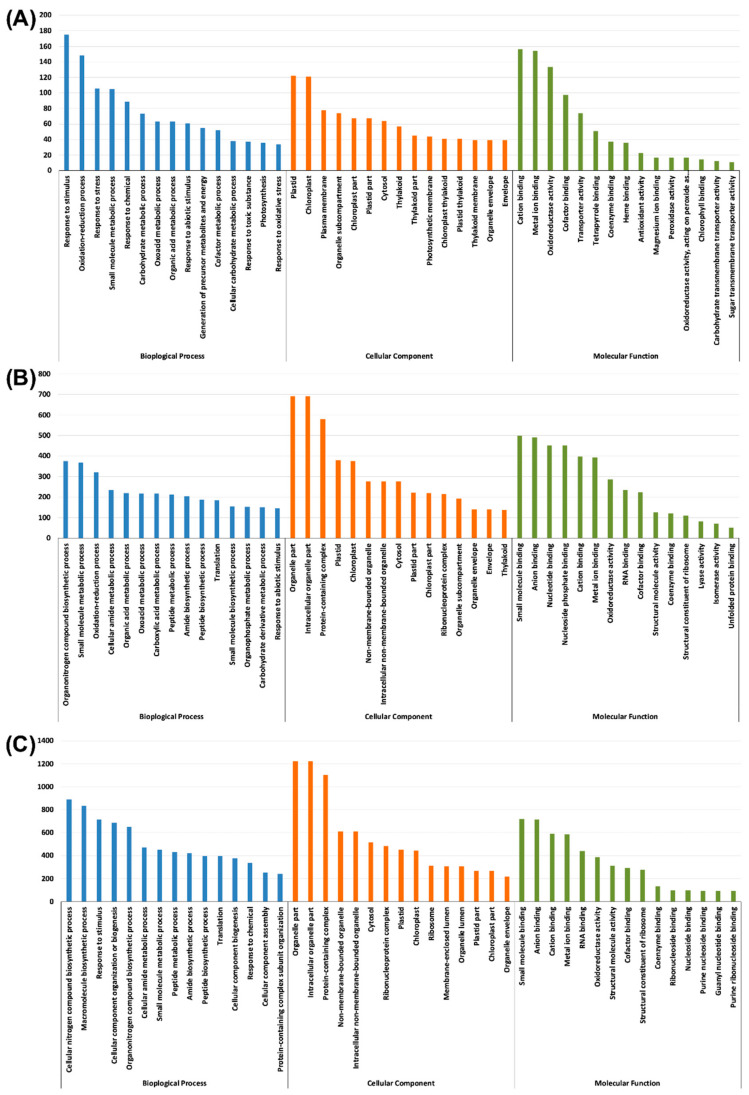
Functional gene ontology annotations of differentially expressed genes (DEGs) under Fe and Zn stresses in the shoot: (**A**)–Zn; (**B**)–Fe; and (**C**)–Fe–Zn. For graphical representation, we have the top 15 significant terms under each category *viz*., biological process (blue), cellular component (orange), and molecular function (green). The complete list of terms along with the significance of FDR < 0.05 are mentioned in the Table S1.

Gene ontology (GO) assignments *viz*., the cellular component, molecular function, and the biological process were used to classify the annotated DEGs based on the biological process, cellular localisation and molecular functions. The top 15 sub-components in each of the categories were depicted as bar graphs (Figure 4 and Figure 5) and all the subcomponents of the categories with a *p*-value for false discovery rate (FDR) enrichment is mentioned in Table S1. Under the biological process category, a co-factor metabolic process and response to abiotic stimulus were common across the stress treatments in both root and shoot tissues (Table S1).

In the cellular component category, Zn and Fe stresses in the root shared the sub-components *viz*., the extracellular region, cell wall, external encapsulating structure, apoplast and protein storage vacuole membrane. Similarly, in the shoot, all the stress treatments showed the cellular components subcategories *viz*., plastid, chloroplast, cytosol, plastid part, organelle envelope, envelope, photosynthetic membrane and chloroplast thylakoid (Table S1).

Furthermore, the molecular function terms *viz*., cation binding, metal ion binding, oxidoreductase activity and the co-factor binding across the stress treatments and tissues (Figure 4 and Figure 5, Table S1). The numbers of GO terms under cation binding, metal ion binding, oxidoreductase activity and co-factor binding were high in –Fe–Zn stress as compared to the individual –Fe and –Zn stresses in both the root and shoot tissues (Table S1). Which confirms that the co-occurrence of Fe and Zn stresses (–Fe–Zn) causes more physiological disturbance than individual stresses. Additionally, a greater number of DEGs in the shoot as compared to the root tissue suggests the presence of diverse physiological and metabolic activities in shoot. Moreover, the significant number of DEGs associated with these mineral homeostasis GO terms indicates the metabolic and physiological demands for Fe and Zn ions under stressed conditions.

**Figure 6:**
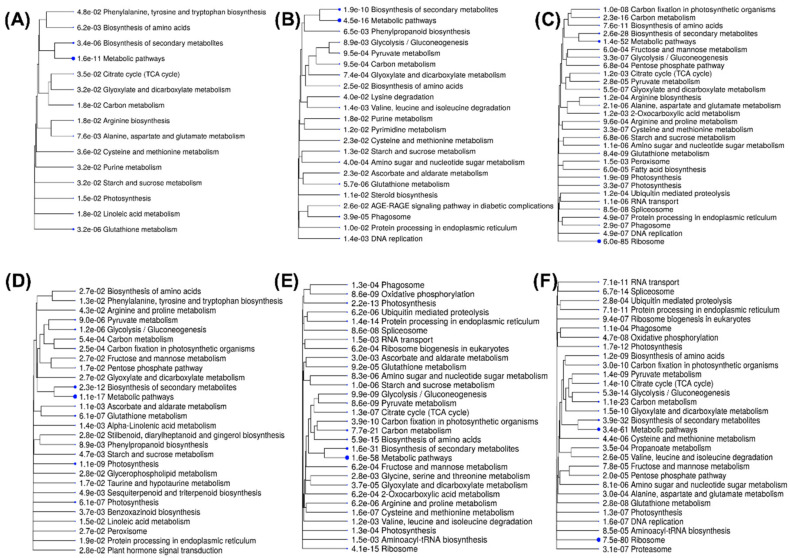
The clustering of KEGG-enriched functional categories of DEGs in the root and shoot under Fe and Zn stresses: (**A**) –Zn in root; (**B**) –Fe in root; (**C**) –Fe–Zn in the root; (**D**). –Zn in the shoot; (**E**) –Fe in shoot; and (**F**) –Fe–Zn in shoot.

The Kyoto Encyclopedia of Genes and Genomes (KEGG) enrichment analysis categorised DEGs into 15, 22 and 30 functional categories in the root under –Zn, –Fe and –Fe–Zn stresses, respectively (Table S2). Similarly, in shoot 28, 30 and 30 functional categories were identified for –Zn, –Fe and –Fe–Zn stress treatments, correspondingly (Table S2). The KEGG enrichment analysis and hierarchical clustering of pathways were undertaken to visualize the relatedness in the enrichment results. In this hierarchical clustering tree, related GO terms were grouped based on the number of common genes. The size of the solid circle corresponds to the enrichment FDR (Figure 6). The KEGG-enriched DEGs in the root are majorly related to themes such as amino acid, carbon and carbohydrate metabolism. Similarly, in the shoot, the major themes fall under amino acid metabolism, carbon and carbohydrate metabolism, photosynthesis and nucleic acid metabolism (Figure 6).

### 2.4. Genes Expression in Response to Fe and Zn Deficiency

Fe and Zn are the vital micronutrients for various metabolisms and the survival of plants. During Fe and Zn deficiency, several genes are known to regulate Fe and Zn homeostasis, phytohormonal regulation and morpho-physiological adaptation. The present investigation recorded the DEGs associated with the mugineic acid pathway, transporters, phytohormone metabolism, photosynthesis and carbohydrate metabolism (Table 1).

#### 2.4.1. Mugineic Acid Pathway and Transporter Genes

The stress treatments (–Zn, –Fe and –Fe–Zn) showed a differential expression of mugineic acid pathway genes in maize seedlings (Figure S3). S-adenosyl-L-homocysteine hydrolysing enzyme *ADENOSYL HOMOCYSTEINASE* (*GRMZM2G015295;* –Fe: 7.51-fold and –Fe–Zn: 30.81-fold) showed the upregulated expression under Fe deficiency in the shoot. O-methyltransferases mediate the conversion of homocysteine to methionine and it serves as a precursor for derivatives of the mugineic acid pathway. *METHYL TRANSFERASES* (*GRMZM2G567452*) showed the upregulation under Fe and Zn deficiency treatments. The *SAMS1* (*GRMZM2G054123*) is known to mediate the conversion of methionine to S-adenosylmethionine (SAM) showed an increased transcript abundance under –Fe (3.92-fold) and –Fe–Zn (–Fe–Zn: 9.42-fold) deficiencies in the shoot. Furthermore, the roots showed a differential expression of *NAS3* (*GRMZM2G054123*) in response to the stress treatments. The upregulation of *YELLOW STRIPE 1* (*ZmYS1*; *GRMZM2G156599*) was observed under Fe deficiency in both roots (8.55-fold) and shoot (2.09-fold). However, in response to the –Fe–Zn treatment, the opposite regulation of *ZmYS1* was observed in the root (35.45-fold) and shoot (–38.26 fold). The expression pattern of *ZmYS1* suggests its higher affinity towards PS-Fe^3+^ and the prominent activity in roots.

In addition to mugineic acid pathway genes, Fe and Zn deficiencies resulted in the differential expression of several transporters. The upregulation of membrane-localised natural resistance associated macrophage protein (NRAMP), *NRAMP TRANSPORTER 1* (GRMZM2G069198) have been observed in both root and shoot. In plants, vacuoles serve as a sequestering site for minerals, including Fe and Zn. Various tonoplast-located transporters mediate Fe and Zn homeostasis in plants. Vacuolar transporters such as V-type ATPases, vacuolar proton pumps (VPPs), tonoplast intrinsic proteins (TIPs) were differentially expressed under the Fe and Zn deficiency. Two TIPs *viz*., *Zm.9197.1.A1_at* and *GRMZM2G027098,* were specifically upregulated in response to Fe deficiency (–Fe and –Fe–Zn) and Zn (–Zn and –Fe–Zn) deficiencies, respectively. Similarly, vacuolar-type H^+^-ATPase (*V-TYPES ATPASE*; *GRMZM2G070360*) showed Fe responsive expression in the shoot. Interestingly, *VPP3* (*GRMZM2G421857*) showed Zn (–Zn and –Fe–Zn) and Fe (–Fe and –Fe–Zn) specific upregulation in the root and shoot, respectively. Ferric chelate reductases (FRO) are the critical component of strategy-I of Fe homeostasis, which showed the upregulated (*GRMZM2G157263*) expression in both roots and shoot under all the stress treatments. Citrate is one of the potent chelators for the mineral transportation in xylem [47]. *CITRATE SYNTHASE 2* (*GRMZM2G064023*) showed a 6.89-fold and 5.41-fold increased expression under –Fe and –Fe–Zn stresses, respectively, in the roots. Among the ABC transporters, the expression of *ABC TRANSPORTER C FAMILY MEMBER 14* (*GRMZM2G142870*) increased the expression by 2.09-fold and 15.46-fold in the root under the –Zn and –Fe–Zn stresses, respectively. The ZIP-like transporter (*ZIP5;* GRMZM2G064382) showed Zn stress-specific upregulation (–Zn: 5.53-fold; –Fe–Zn: 3.85-fold) in the root, whereas the metal ion transmembrane transporter activity (*GRMZM2G178190*) was observed under Fe deficiencies only. Fe deficiency in the shoot (–Fe and –Fe–Zn) resulted in the upregulation of *MITOCHONDRIAL PHOSPHATE TRANSPORTER* (*GRMZM2G015401*; –Fe: 3.46-fold and –Fe–Zn: 10.28-fold).

#### 2.4.2. Phytohormones

Phytohormone metabolism shows the dynamic responses to Fe and Zn availability, which enable the plants to adapt to Fe and Zn deficiencies. In the present investigation, Fe and Zn deficiencies have altered the expression of various genes associated with various phytohormone metabolism *viz*., ethylene, auxin, gibberellins, cytokinin (Figure S4). The roots under Zn (–Zn and –Fe–Zn) and Fe (–Fe and –Fe–Zn) deficiencies upregulated the *CYTOKININ OXIDASE 2* (*GRMZM2G050997*) and *CYTOKININ OXIDASE 3* (*GRMZM2G167220*) expressions, respectively. The *CYTOKININ-O-GLUCOSYLTRANSFERASE 2* (*GRMZM2G041699*) showed downregulation across the stress treatments.

The upregulation of *Aux/IAA* genes *IAA13* (*GRMZM2G077356*) and *IAA24* (*GRMZM2G115354*) in the shoot (–Zn: 2.59-fold; –Fe–Zn: 9.07-fold) and root (–Zn: 2.06-fold; –Fe–Zn: 2.13-fold), respectively, was observed specifically to Zn deficiency. Similarly, *IAA17* (*GRMZM2G147243*) showed Zn-deficiency induced expression in root tissue (–Zn: 41.19-fold; –Fe–Zn: 11.45-fold). The pin-formed (PIN) protein *PIN1b* (*GRMZM2G074267*) crucial for the polar transport of auxins showed the downregulated expression under –Fe (–3.48) and –Fe–Zn (–5.35) in the root. The repression or non-significant expression of AIR12 (GRMZM2G427451) was observed in the root under –Fe and –Fe–Zn stresses.

Ethylene is another important class of hormones mediating plants’ adaptation under Fe and Zn deficiencies. The –Fe and –Fe–Zn stresses resulted in the high expression of *ETHYLENE RECEPTOR* (*GRMZM2G102601*) in the shoot (–Fe: 5.50-fold; –Fe–Zn: 3.43-fold). On the contrary, *ETHYLENE RESPONSE FACTOR* (*GRMZM2G052667*) showed an upregulation in the roots under –Fe (2.86-fold) and –Fe–Zn (3.61-fold) stresses. Ethylene responsive factors (ERF) mediate stress signals. The repression of ERFs has been recorded in response to Fe and Zn deficiencies in a tissue-specific manner. *ERF-LIKE PROTEIN* (*GRMZM2G025062*) was downregulated only in roots, whereas *ETHYLENE-RESPONSIVE FACTOR-LIKE PROTEIN 1* (*GRMZM2G053503*) was downregulated in both the root and shoot. Furthermore, Zn stresses in the shoot (–Zn and –Fe–Zn) upregulated the gibberellin receptor gene *GID1L2* (*GRMZM5G831102*; –Zn: 5.00-fold; –Fe–Zn: 15.97-fold).

#### 2.4.3. Photosynthesis and Carbohydrate Metabolism

Several photosynthesis and carbohydrate metabolism-related genes showed differential expression under Fe and Zn stresses (Figure S5). Under –Fe (2.35-fold) and –Fe–Zn (20.44-fold) stresses, *FERROCHELATASE* (GRMZM2G113325) showed the upregulated expression in roots. In photosynthesis, ferredoxins transfer the electrons from photo-reduced photosystem-I to ferredoxin NADP^+^ reductase in which NADPH is produced for CO_2_ assimilation. *FERREDOXIN 3* (*GRMZM2G053458*) showed the enhanced expression under –Fe (25.82-fold) and –Fe–Zn (17.88-fold) stress in the shoot. Similarly, the cytochrome complexes mediate the electron transfer between PSII and PSI. Fe stresses in the shoot (–Fe, –Fe–Zn) resulted in the increased accumulation of *APOCYTOCHROME F PRECURSOR* transcripts (*GRMZM2G448174*) in the shoot. Carbonic anhydrases (CAs) are Zn metalloenzymes that catalyse the interconversion of CO_2_ and HCO_3_^-^ in plants. The root under –Zn and –Fe–Zn deficiencies and shoot under –Zn and –Fe deficiencies upregulated the *CARBONIC ANHYDRASE* (*GRMZM2G121878*), whereas –Fe–Zn stress in the shoot showed the downregulation of *CARBONIC ANHYDRASE* (*GRMZM2G121878*). The activity of carbonic anhydrase mediates the supply of CO_2_ to Rubisco (Ribulose bisphosphate carboxylase). The –Fe and –Fe–Zn deficiencies resulted in higher *RIBULOSE BISPHOSPHATE CARBOXYLASE SMALL SUBUNIT 2* (*GRMZM2G113033*) transcripts accumulation. However, as compared to combined Fe and Zn deficiency (–Fe–Zn), Fe deficiency (–Fe) showed very high expressions. The chlorophyll *a*/*b*-binding proteins are the apoproteins of the light-harvesting complex of photosystem II. In the present investigation, the *CHLOROPHYLL A-B BINDING PROTEIN 6A* (*ZmAffx.1219.1.S1_s_at*) was upregulated under all the stresses in the shoot. The various proteins associated with photosynthesis co-translationally targeted the chloroplast via signal recognition particles. The transcripts of *CHLOROPLAST SIGNAL RECOGNITION PARTICLE SUBUNIT* (*GRMZM2G145460*; *cpSRP54*) were repressed across the stresses.

Fe stress resulted in a higher expression of DEGs associated with carbohydrate metabolism. The Fe deficiency (–Fe, –Fe–Zn) showed a consistently greater accumulation of transcripts of the glycolysis pathway in the shoot. The majority of glycolytic enzymes *viz*., *PHOSPHOGLYCERATE KINASE* (*GRMZM2G382914*), *ENOLASES* (*GRMZM2G064302*, *GRMZM2G048371*), *PHOSPHOHEXOSE ISOMERASE* (*GRMZM2G065083*), TRIOSEPHOSPHATE *ISOMERASE* (*GRMZM2G030784*), *GLYCERALDEHYDE-3-PHOSPHATE DEHYDROGENASE* (*GRMZM2G051004*) showed an enhanced expression in the shoots under –Fe and –Fe–Zn deficiencies. However, Zn deficiency did not show any consistent expression of glycolysis-associated transcripts. Furthermore, the roots enhanced the transcripts of *CARBOHYDRATE TRANSPORTER* (*GRMZM2G336448*) in response to Fe deficiency (–Fe: 4.22-fold; –Fe–Zn: 2.12-fold). On the other hand, genes associated with polysaccharide synthesis and cell wall biogenesis *viz*., *UDP-GLUCOSE 6-DEHYDROGENASE* (*GRMZM2G328500*; *GRMZM5G862540*), *UDP-GLUCURONIC ACID DECARBOXYLASE 1* (predicted; *GRMZM2G165357*) and *POLYGALACTURONATE 4-ALPHA-GALACTURONOSYLTRANSFERASE* (*GRMZM2G076276*) showed increased transcripts accumulation in the shoot under –Fe and –Fe–Zn stresses. The Zn stresses in the shoot (–Zn and –Fe–Zn) resulted in the higher expression of *CELLULOSE SYNTHASES* in the shoot (*GRMZM2G424832*, *GRMZM2G018241*, *GRMZM2G142898*).

### 2.5. Regulation and Interaction of Fe and Zn Transporters in Maize

**Figure 7:**
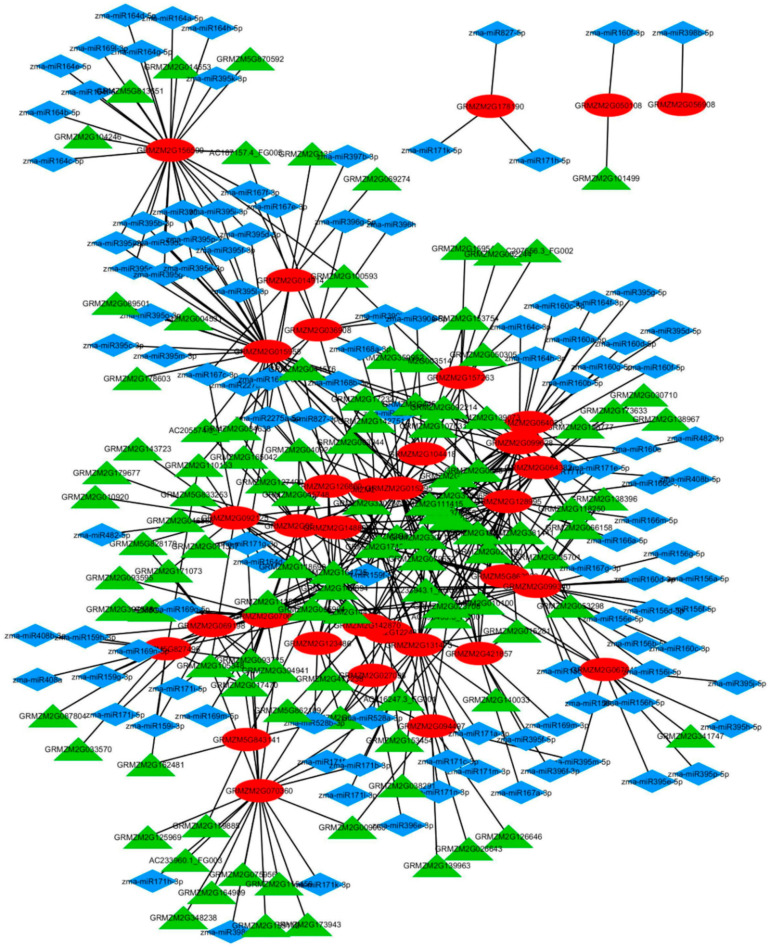
The transcription factors (TFs) and miRNAs mediated the regulation of differentially expressed transporters under Fe and Zn deficiency in maize: the regulatory network is characterized by 148 miRNA–target gene and 306 TF–target genes interactions. The green triangles represent the TFs, the red ovals represent the target transporters and mugineic acid pathway genes, and the blue diamonds represent miRNAs.

The gene-regulatory network (GRN) is constructed to visualize the regulatory relationships of miRNA and the transcription factors with their downstream differentially expressed transporter and mugineic acid pathway genes under Fe and Zn deficiency. GRN revealed a total of 454 interactions of transporters with miRNA and transcription factors (TFs) (Tables S3 and S4; Figure 7). The highest number of total degrees (31) were observed for the *ZINC TRANSPORTER 4* (*GRMZM2G015955*), *METALLOTHEIONINE LIKE PROTEIN* (*GRMZM2G099340*) and *METHIONINE t-RNA LIGASE* (*GRMZM2G099628*). However, the *ZmYS1* (*GRMZM2G156599*) and *VACUOLAR PROTON-TRANSPORTING V-TYPE ATPASE, V1 DOMAIN* (*GRMZM2G128995*) showed the highest degrees (23) with miRNAs and TFs, respectively. Therefore, these genes are the potential hub nodes in the regulation of the Fe and Zn homeostasis in maize. TF and miRNA and their interactions are adding the regulatory complexity to transporters genes. The regulatory network showed the highest gene–miRNA interactions with miRNAs of the zma-miR395 family (36) and the highest gene–TF interactions with ERFs (104). Hence, zma-miR395 and ERFs are the major regulatory elements in transporter and mugineic acid pathway regulatory network of maize. Furthermore, transporters’ regulatory network in the present investigation showed the regulation of genes with a common expression pattern by a common transporter under Fe and Zn deficiency although this may not be a universal phenomenon (Figure 7; Table S3). For instance, six transporter genes (*GRMZM2G015295*, *GRMZM2G067546*, *GRMZM2G070605*, *GRMZM2G099340*, *GRMZM2G099628*, *GRMZM2G128995*) showing Fe specific upregulation showed a common TF-mediated regulation through EREB142 (GRMZM2G010100). Conversely, many of the transporter genes’ expression is regulated by several miRNA and TFs.

## 3. Discussion

### 3.1. Fe and Zn Deficiencies Shifted the Expression Pattern of the Transcriptome in Root and Shoot

The current investigation presents a genome-wide transcriptome response of individual and simultaneous Fe and Zn deficiencies in both the root and shoot tissues. The experiments were performed with the aim of understanding the changes in the global transcripts’ level under –Zn, –Fe and –Fe–Zn stresses, and to decipher the expression of transcripts associated with Fe and Zn homeostasis, phytohormone metabolism, photosynthesis and carbohydrate metabolism. Genome-wide expression assay revealed a higher number of DEGs in the shoot (8200) as compared to root (7316) for –Fe and –Zn stresses indicating greater physiological demand for Fe and Zn minerals in the shoot over the root. The shoot controls most of the essential physiological activities *viz*., photosynthesis, chlorophyll biosynthesis, carbohydrate and cytochrome biosynthesis, and that of several plant pigments. Moreover, several enzymes employed in the shoot physiological activities required Fe and/or Zn as co-factor(s) or their integral component(s) [48,49]. On the other hand, high metabolic and physiological pressure on the root transportome and membrane-bound proteins to meet the Fe and Zn ions under –Fe–Zn stress could have resulted in contrasting trends of a higher number of DEGs in the roots as compared to shoot. The results of the present investigations are in contrast with those of Zheng et al. [32], who reported a higher number of DEGs in the root as compared to the shoot under Fe and P interactions in rice. The functional classification of DEGs in the current investigation revealed many of the GO terms associated with ion uptake and homeostasis *viz*., cation binding, metal ion binding, oxidoreductase activity etc. The deficiency of Fe and Zn alters the expression of genes associated with Fe and Zn homeostasis to regulate the plant physiological and metabolic requirement of Fe and Zn ions [2,3,23,26,27].

### 3.2. Fe and Zn Stresses Alter the Root Length via Differential Expression of Phytohormonal Genes 

The plant root system is a vital component for the uptake and transport of nutrients from the soil and also exhibits considerable plasticity in response to endogenous and environmental signals [50]. The notable changes in the root length of maize inbred line SKV-616 under –Zn, –Fe and –Fe–Zn stresses were associated with the differential expressions of several genes of hormonal metabolism (Figure 1C, Table 1). Owing to Zn stresses (–Zn, –Fe–Zn), the upregulated expression of *IAA17* (*GRMZM2G147243*) and *IAA24* (*GRMZM2G115354*) was observed in the root tissue. *IAA17* prevents the initiation of lateral roots by preventing the binding of AXR3 to *auxin-responsive element* (*ARE*) [51]. Similarly, the *IAA3* gain of function in rice causes growth defects in crown roots [52,53]. Furthermore, *AtIAA8* is an ortholog of *ZmIAA24* in *Arabidopsis* which has also been reported as a negative regulator of lateral root formation [54]. In addition to the regulation of root phenotype, auxins also serve as signalling molecules in mineral deficiency in plants. The enhanced expression of *IAA13* (*GRMZM2G077356*) and *IAA17* (*GRMZM2G147243*) in –Zn and –Fe stress-specific manner in the shoot indicate their probable role in a shoot-to-root auxin signalling under Zn and Fe deficiency, respectively. In wheat, the application of IAA to shoots of Fe-sufficient plants resulted in the enhanced release of PS in roots [38]. Furthermore, the opposite regulation pattern *IAA* genes could be associated with variation in the spatial responses. In contrast to auxins, cytokinins play an inhibitory role in the lateral root initiation and development [55,56]. The maize seedlings showed Zn and Fe stress-specific expression of *CYTOKININ OXIDASES (CKXs)* in root tissues. Remarkably, *CKX3* (*GRMZM2G167220*) showed a higher upregulation in response to Fe stresses (–Fe, –Fe–Zn) as compared to *CKX2* (*GRMZM2G050997*) under Zn stresses (–Zn, –Fe–Zn). In *Arabidopsis,* the overexpression of cytokinin-degrading enzyme encoding gene *CKX* in the lateral root primordia (LRP) results in reduced cytokinin levels and the elimination of cytokinin signalling [57,58], thereafter which enhances the lateral root density by reducing the distance between the adjacent LRP cells [59,60].

The synthesis of ethylene is perceived by membrane-bound ethylene receptors that initiate the ethylene signalling pathway. The higher expression of *ETHYLENE RESPONSE FACTOR* (*GRMZM2G052667*) in –Fe and –Fe–Zn stresses of the root appear to have contributed towards reduced root length. In maize, ethylene-responsive factors (*ERF1*) play a vital role in stress signalling pathways [61]. Similarly, in tomato, the overexpression of ethylene-responsive factors *LeERF2/TERF2* showed the increased synthesis of ethylene [62]. Furthermore, the inverse association between higher ethylene biosynthesis and primary root growth through decreased cell proliferation and elongation in root apical meristem was observed in *Arabidopsis* [63,64].

Gibberellins increase primary root length through the cell elongation and proliferation of root meristem and reduced levels of GAs result in shorter primary roots [65]. GA signalling in plants involves the GA receptor *GIBBERELLIN INSENSITIVE DWARF1* (*GID1*), GA signalling repressor DELLA proteins and ubiquitin ligase complex [66]. The binding of GID1 to bioactive GA, causing the degradation of DELLA proteins, which subsequently initiates the GA signalling in plants. In the absence of bioactive GA, DELLA proteins repress the GA responses [67]. Hence, the downregulation of *GID1L2* (*GRMZM5G831102*) under –Fe and –Fe–Zn stresses in the root indicate a direct association between the reduced root length and of GA signalling in maize. Previous findings also reported an association between the reduced root growth and endogenous GA level under Zn and Fe deficiency in maize [68] and rice [67], respectively.

### 3.3. Fe and Zn Deficiency Affect Photosynthesis and Carbohydrate Metabolism Through Altered Expression of Key Genes

In the present investigation, the enhanced expression of *FERROCHELATASES* (*GRMZM2G113325*), *APOCYTOCHROME F* (*GRMZM2G448174*), *CHLOROPHYLL A-B BINDING PROTEIN 6A* (*ZmAffx.1219.1.S1_s_at*) were observed under Fe deficiency stresses (–Fe and –Fe–Zn). APOCYTOCHROME F (GRMZM2G448174) is the precursor of cytochrome b6-f complex, which facilitates electron transfer between photosystem II (PSII) and PSI [69]. Similarly, ferredoxins involved in electron transport from the photo-reduced photosystem-I to NADP^+^ reductases. The chlorophyll a-b binding protein 6A (ZmAffx.1219.1.S1_s_at) acts as a component of the light-harvesting complex (LHC) which is involved in the formation of a full-size NAD(P)H dehydrogenase-PSI super complex (NDH-PSI) that triggers cyclic and chlororespiratory electron transport in chloroplast thylakoids, especially under stress conditions [70]. Therefore, the Fe and Zn deficiency-induced overexpression of *APOCYTOCHROME F, CHLOROPHYLL A-B BINDING PROTEIN 6A* and *FERREDOXIN 3* affects photosynthesis by disrupting the cellular redox poise and electron transfer mechanisms [69,71].

The increased expression of *CARBONIC ANHYDRASE* (CA; *GRMZM2G121878*) during Zn deficiency (–Zn and –Fe–Zn) requires Zn^2+^ ions as co-factors for its functional activity. CA catalyses the first biochemical step of the carbon fixation in C_4_ plants. As a co-factor in CA, Zn creates a proton H^+^ and a nucleophilic hydroxide ion. The nucleophilic water molecules attack the carbonyl group of carbon dioxide to convert it into bicarbonate. Additionally, the H^+^ ions reduce the pH, and inactivate the enzymes associated with the hydrolysis of starch and glucose, and subsequently reduce the osmotic gradient of cells and stomatal closure [72].

Rubisco is the most prominent protein on the Earth and serves as the primary engine of carbon assimilation through photosynthesis. Fe deficiency resulted in higher accumulation of RuBisCO subunit transcripts (*GRMZM2G113033*, *GRMZM2G095252*) in the shoot. However, previous reports in soybean and sugar beet pointed the reduced as well as increased RuBisCO protein content under Fe deficiency, respectively [73,74]. Fe and Zn deficiency-induced the disruption in the protein synthesis machinery, which could be the reason for reduced RuBisCO proteins’ accumulation. In barley and rice, the accumulation of RuBisCO was higher in the older leaves of Fe-deficient plants than in the older control leaves, which suggests that RuBisCO accumulation is not decreased significantly by Fe deficiency. Therefore, there is a non-significant association between chlorophyll concentration and RuBisCO accumulation [75,76]. Overall, the photosynthetic machinery is known to be highly sensitive to Fe deficiency as compared to Zn owing to the role of Fe ions as co-factors in the key components of the photosynthetic machinery. There are two or three Fe atoms per PSII, 12 Fe atoms per PSI, five Fe atoms per cytochrome complex b6–f (Cyt b6–f) and two Fe atoms per ferredoxin molecule as reported by Briat et al. [77] and Krohling et al. [71].

The upregulation of glycolytic pathways genes *viz*., PHOSPHOGLYCERATE KINASE (GRMZM2G382914), ENOLASES (GRMZM2G064302, GRMZM2G048371), PHOSPHOHEXOSE ISOMERASE (GRMZM2G065083), TRIOSEPHOSPHATE ISOMERASE (GRMZM2G030784), and GLYCERALDEHYDE-3-PHOSPHATE DEHYDROGENASE (GRMZM2G051004) under Fe deficiency is associated with Fe deficiency-associated metabolic pressure. The Fe deficiency increases the demand for reducing power, and ATP owing to the enhanced expression of Fe acquisition mechanisms and increased organic acids content [76]. Fe deficiency enhanced the activity of the glycolysis pathway to meet the reducing ATP and the enhanced carboxylation. However, the uptake and transport of Fe demand the chelators and organic acids from the carbohydrate metabolism as compared to free ionic movement of Zn and which could be one of the reasons for the greater response of the glycolytic pathway to Fe deficiency as compared to Zn deficiency in plants.

The Zn deficiency in the present investigation resulted in the enhanced expression of cellulose synthase enzymes (CESAs) (*GRMZM2G424832*, *GRMZM2G018241*, *GRMZM2G142898*) to meet the Zn requirement for cellulose synthesis. The flexibility of primary cell walls in plants is necessary to adapt the plants under stress conditions to withstand the turgor pressure. Cellulose is the main constituent of the cell wall and is directly synthesized at the plasma membrane [78]. The CESAs carry the terminal Zn-binding domain, which oligomerizes the CESAs under oxidative stress [78,79]. Likewise, Fe deficiency treatments (–Fe, –Fe–Zn) also enhanced the accumulation of the transcripts of other non-cellulosic constituents of cell walls *viz*., pectins (*POLYGALACTURONATE 4-ALPHA-GALACTURONOSYLTRANSFERASE*: *GRMZM2G076276*), xylans (*PREDICTED UDP-GLUCURONIC ACID DECARBOXYLASE 1*: *GRMZM2G165357*), arabinoses (*UDP-ARABINOPYRANOSE MUTASE 3*: *GRMZM2G073725*).

Sucrose-phosphate synthases (*GRMZM5G875238*, *GRMZM2G140107*) involved in sucrose biosynthesis showed higher upregulation under Fe deficiency. The Fe deficiency in *Arabidopsis* increases sucrose accumulation in the roots and subsequently promotes the auxin signalling cascades [80]. Therefore, the enhanced accumulation of sucrose may act as one of the junctions between carbohydrate metabolism and phytohormonal signalling under Fe and Zn deficiency in plants.

### 3.4. Differential Expression of Transporters under Fe and Zn Stresses in Maize

The release of Fe and Zn chelators are crucial in root tissues. The expression pattern of *ADENOSYL HOMOCYSTEINASE* (*GRMZM2G015295*), *O-METHYL TRANSFERASES* (*GRMZM2G567452*), *SAMS* (*GRMZM2G054123*) and *ZmYS1* (*GRMZM2G156599*) suggest higher sensitivity to Fe deficiency. Furthermore, the upregulation of *NAS3* (*GRMZM2G050108*) was observed under –Zn and –Fe–Zn treatments, whereas the *NAS1* (*GRMZM2G034956*) expression was induced under –Fe deficiency only. Interestingly, *NAS3* expression was highly repressed in the shoot under –Fe–Zn treatment suggesting the possible spatial regulation of *NAS3*. The upregulation of the mugineic acid pathway genes is an adaptive strategy in grasses to overcome the Fe and Zn starvation [81,82]. Furthermore, the activity of transporter and chelator genes during Fe and Zn deficiencies occurs in a stress-specific spatiotemporal manner. Nicotianamine is the potent chelator and mediates the uptake and transport of Fe and Zn in plants [8]. The dual regulation of *NAS* genes in response to Fe deficiency has also been reported in maize [83]. In addition to uptake and transport, *NAS* genes also known to be engaged in the accumulation of Fe into the seed [84]. Hence, NA could be one of the significant contributors to the long-distance transport of Fe and Zn in maize. The root tissue showed an enhanced expression of *NRAMP TRANSPORTER 1* (*GRMZM2G069198*) under Zn deficiency. However, for Fe deficiency, the expression was prominent in the shoot. Similarly enhanced upregulation was also observed in maize under Fe deficiency [36]. In *Arabidopsis* and rice, *NRAMP1* is a key transporter for Fe homeostasis [85,86]. Additionally, *NRAMP1* showed to translocate both divalent and trivalent ions in peanut [87] and *Arabidopsits* [88].

Among Fe and Zn chelating complexes, the upregulated expression of *CITRATE SYNTHASE 2* (*GRMZM2G064023*) was observed in response to Zn (–Zn, –Fe–Zn) and Fe (–Fe, –Fe–Zn) deficiencies in the root and shoot, respectively, whereas the expression levels were very high under Fe deficiency in the shoot (–Fe). Both strategy-I and II plants hold Fe–citrate complexes in the xylem sap [89,90]. Fe starvation has been reported to increase the citric acid level in the xylem to enhance Fe mobility in plants [91,92]. In maize, Fe^3+^–citrate and Fe^3+^–phytosiderophore are the Fe form in the xylem sap [93]. Similarly, the xylem of *Thlaspi caerulescen* showed ~21% of total cellular Zn as Zn-citrate complex [94].

The reduction of Fe^3+^ to more soluble Fe^2+^ ions by membrane bound *FERRIC CHELATE REDUCTASE 2* (*GRMZM2G157263*) is crucial for the subsequent Fe transport as Fe^2+^-NA and electron transfer mechanism. Oligopeptide transporter family proteins can transport minerals in the form of metal–NA complexes. *OPT* (*GRMZM2G148800*) showed an upregulated expression response to Fe deficiency (–Fe, –Fe–Zn) in the root. However, the downregulation was observed in the shoot under Zn deficiency (–Zn, –Fe–Zn). The accumulation of *ZINC-TRANSPORTER 4* (*GRMZM2G015955*) transcripts under Zn deficiency in the root and shoot indicates the major function in Zn homeostasis. Similarly, the transcripts of *ZIP5* (*GRMZM2G064382*) were enhanced under Zn deficiency in the roots (–Zn, –Fe–Zn), whereas anti-directional regulation was observed in the shoot. Several authors reported a role of ZIP transporters in the homeostasis of divalent ions *viz*., Fe, Zn, Mg [17,25,26,27,95]. In maize, Li et al. [96] showed that the overexpression of *ZmZIP5* enhances the Zn and Fe accumulation. Similarly, the overexpression of *ZIP5* and *ZIP9* genes in rice showed an increased accumulation of Zn over the control plants [25,26,27].

Vacuoles in the cell help to regulate Fe and Zn flux. Transporters located in the vacuole membrane play an important role in long-distance transport in addition to intracellular metal homeostasis [97]. During Fe and Zn deficiencies, the enhanced expression of vacuole transporters is necessary for the mobilisation of stored Fe and Zn ions to meet the metabolic demands [98]. The enhanced expression of *TIPs* in the shoot showed a possible association of *Zm.9197.1.A1_at* and *GRMZM2G027098* in regulating Fe and Zn homeostasis, respectively. Studies showed that the enhanced expression of *ZmTIP1* facilitates the rapid intracellular osmotic equilibration and permits quick water flow through the vacuoles [99]. The *VACUOLAR PROTON PUMP 3* (*GRMZM2G421857*) transcripts increased in response to Zn and Fe deficiencies in the root and shoot, respectively; and Fe deficiency enhanced the expression of *V-TYPE PROTON ATPASE SUBUNIT E3* (*GRMZM2G070360*), *VACUOLAR ATP SYNTHASE SUBUNIT B* (*GRMZM2G094497*), and *VACUOLAR PROTON-TRANSPORTING V-TYPE ATPASE, V1 DOMAIN* (*GRMZM2G128995*) in the shoot. The specialized functions of vacuoles depend on the tissue and cell type and their developmental stage. All vacuoles seem to hold the majority of the proton pump transporters and differ in their function, depending on the type of vacuole in which they reside [100].

### 3.5. Hormonal Signalling and Homeostasis Networks Involved in the Fe and Zn Deficiency Regulatory Network

Transcriptome response to Fe and Zn interaction helps in understanding the association and crosstalk of Fe and Zn metabolism in maize. Very few attempts have been made to decipher Fe and Zn interactions in crops at the transcriptome strata. At the transcriptome level, the Fe and Zn stresses acted additively as the number of genes differentially expressed were quite high in the combined Fe and Zn stress (–Fe–Zn) as compared to the individual Fe (–Fe) or Zn (–Zn) stress. However, the direction of the individual gene(s) expression pattern varied with the genes. Many of the key genes associated with Fe and Zn metabolisms were highly downregulated and showed an opposite regulation pattern under both Fe and Zn (–Fe–Zn) deficiencies in the shoot (Table 1). It is known that Fe and Zn act as co-factors of enzymes associated with various metabolic processes and the expression of key genes [48,49]. The simultaneous deficiency of both Fe and Zn could hinder the deficiency-responsive enhanced expression of key genes through altering the respective regulatory enzymes expression. Furthermore, the exertion of high regulatory and metabolic pressure during Fe and Zn stresses to sustain the key metabolisms in the shoot could have resulted in the downregulation and opposite regulation of various key genes. As a support to the findings, the inverse regulation of auxin metabolism by Fe and Zn deficiency was reported in bean [49] as well as in rice [101].

The expression of transporter genes under Fe and Zn deficiency is regulated by various regulatory miRNA and TFs. The binding of miRNA to respective transcripts suppresses the expression of target genes. The maximum interactions was observed for zm-miR395 family miRNAs. The zm-miR395, is a regulator of key proteins *viz*., low-affinity sulphate transporter (*AST68*) and ATP sulfurylases (*APS1*, *APS3* and *APS4*) associated with sulphur homeostasis [102] and Fe–S clusters in plants. Fe is present in the centre of Fe–S clusters which act as electron acceptors and donors in several cellular processes including photosynthesis, respiration, sulphate assimilation and ethylene biosynthesis [77]. Furthermore, the binding of Zn ions to scaffold assembly protein ISU1 is more crucial for the stability of Fe–S cluster [103]. Fe deficiency results in the downregulation of miR395 in *Arabidopsis* [104]. The Fe and Zn deficiency-induced downregulation of miR395 is associated with the upregulation of its target genes *viz*., *ZIP4* (*GRMZM2G015955), ZmYS1* (GRMZM2G156599), *VACUOLAR SORTING RECEPTOR HOMOLOG 1* (*GRMZM2G067546*), *VACUOLAR PROTON PUMP 3* (*GRMZM2G421857*), *CITRATE SYNTHASE 2* (*GRMZM2G064023*).

Similarly, among all the transcription factors, ERFs showed the highest interactions (104) with transporters and mugineic acid pathway genes. In the present and previous investigations, Fe and Zn deficiency resulted in differential expression ERFs [34]. Ethylene acts as an important signalling molecule in the regulatory networks of transporter and hormones. The regulatory network showed gene–ERF interactions with *ZINC TRANSPORTER 4* (*GRMZM2G015955*), *ZRT-IRT-LIKE PROTEIN 5* (*GRMZM2G064382*), *METAL ION TRANSPORTER* (*GRMZM2G122437*), *VACUOLAR PROTON-TRANSPORTING V-TYPE ATPASE, V1 DOMAIN* (*GRMZM2G128995), ABC TRANSPORTER C FAMILY MEMBER 14* (*GRMZM2G142870*), *OLIGOPEPTIDE TRANSMEMBRANE TRANSPORTER* (*GRMZM2G148800*), *FERRIC-CHELATE REDUCTASE* (*GRMZM2G157263*), *VACUOLAR PROTON PUMP 3* (*GRMZM2G421857*) and *MITOCHONDRIAL PHOSPHATE TRANSPORTER* (*GRMZM2G015401*). Many of the ERFs are known to acts as repressor proteins for transporters and ethylene biosynthesis through feedback inhibition [105]. Therefore, the downregulation of ERFs (GRMZM2G025062, GRMZM2G053503) could be associated with the enhanced regulation of transporters and ethylene synthesis under Fe and Zn deficiencies. Furthermore, in rice, the upregulated expression of Fe and Zn transporters was observed under enhanced ethylene synthesis [41]. Additionally, the ethylene is known to alter the auxin signalling cascades in plants [106]. The sensitivity and spatiotemporal expression of regulatory elements could also play a role in the dual regulation of transporter genes. Furthermore, the regulation of genes associated with different adaptive physiological pathways by miRNA and TFs enhances the system’s complexity. In addition to regulatory elements, regulatory enzyme systems also increase the interactions in plants. Fe and Zn deficiency has been reported to alter the hormonal signalling through the accumulation of sugars [80]. These complex crosstalks and interactions through various pathways need to be addressed through reverse genetic approaches.

## 4. Materials and Methods

### 4.1. Plant Material and Stress Treatment

Genotype SKV-616, a quality protein maize (QPM with superior protein quality) inbred line was selected to carry out the genome-wide expression assay owing to its ability to accumulate a moderately high level of Fe and Zn concentration [107]. The seeds of a maize inbred SKV-616 were surface sterilised with 1% NaClO solution for 5 min followed by six rinses with Milli-Q water (18.2 MΩ). Seeds were germinated on a Milli-Q water (18.2 MΩ)-soaked filter paper roll. The seedlings were germinated and grown for seven days in the darkroom at 25 °C. The uniform seedlings were transferred to a nutrient solution containing 0.7 mM K_2_SO_4_, 0.1 mM KCl, 0.1 mM KH_2_PO_4_, 2.0 mM Ca(NO_3_)_2_, 0.5 mM MgSO_4_, 10 μM H_3_BO_3_, 0.5 μM MnSO_4_, 0.2 μM CuSO_4_, 0.5 μM ZnSO_4_, 0.05 μM Na_2_MoO_4_, and 0.1 mM Fe^3+^-EDTA. The pH of the nutrient solution was maintained at 5.5 throughout the experiment with 1 M HCl [108]. Fe^3+^-EDTA and ZnSO_4_ were excluded from the media to induce Fe (–Fe +Zn) and Zn (+Fe–Zn) deficiencies, respectively. For induction of simultaneous Fe and Zn stresses (–Fe–Zn), both Fe^3+^-EDTA and ZnSO_4_ were removed from the complete solution. After the seeds germinated, the plants were transferred to the respective treatment solution and grown until the clear expression of Fe and Zn deficiency symptoms (12 days).

### 4.2. Morpho-Physiological Characterisation

All the morphophysiological parameters were recorded on five plants grown in hydroponic culture with three data points per seedling. The chlorophyll content was measured with soil plant analysis development (SPAD) chlorophyll meter (SPAD-502 Plus chlorophyll meter, Konica Minolta Sensing Inc., Osaka, Japan). The readings were taken on the top, middle and base of the fully expanded top leaf in each seedling. The same leaf was used for measuring the photosynthesis rate, transpiration rate and stomatal conductance with a portable photosynthesis system (LI-6400, Nebraska, USA). The maximal quantum efficiency of the photo-system II (PS II) photochemistry (*Fv/Fm*) was recorded on 30 min dark-adapted leaves using a portable photosynthesis system with fluorescence attachment (LI-6400, LI-COR, Nebraska, USA). The root length was measured manually with the measuring scale. The statistical analysis was performed with a two-sample t test with the assumption of unequal variances with all possible comparisons among the control and treatments.

### 4.3. Genome-Wide Expression Assay Using Affymetrix GeneChip Maize Genome Array

The microarray experiment was designed as a two-factor experiment with the four possible treatments among Fe and Zn combination *viz*., 1) +Fe+Zn, 2) –Zn, 3) –Fe and 4) –Fe–Zn. The total RNA was isolated in triplicates separately from the control and stress-treated root and shoot tissues of 19 day-old seedlings with a stress period of 12 days (50 mg each) using the RNeasy Mini Kit (Qiagen, Hilden, North Rhine-Westphalia, Germany) as per manufacturer’s guidelines. The RNase free DNAse treatment with a Thermo Scientific kit (La layette, USA) was given to each RNA sample to eliminate the residual DNA present in RNA samples. Each DNAse-treated RNA sample obtained was examined for quantity using a NanoDrop 1000 spectrophotometer (Thermo Scientific, Wilmington, Delaware, USA). RNA samples with a A260/280 ratio of 1.8 to 2.1 in were considered for further analysis. Affymetrix GeneChip maize genome arrays (Affymetrix Inc., Santa Clara, California, USA) were used in three replications for each treatment to ensure the reproducibility and quality of the chip hybridisation using standard Affymetrix protocols (3’IVT protocol on GeneChip Fluidics Station 450; scanning on Affymetrix GSC3000). For the microarray experiment, ~300 ng of total RNA was biotin-labelled for GeneChip analysis and 10 µg of purified fragmented cRNA was used for hybridisation. All expression data were derived based on the Minimum Information About a Microarray Experiment (MIAME) guidelines [109].

### 4.4. Microarray Data Analyses

The GeneChip Operating Software (GCOS, Affymetrix GeneChip operating software with autoloader ver. 1.4, manual) was used to generate the CEL file for each of the scanned microarray chips. The raw CEL files containing probe hybridisation intensities from 24 chips were imported into the R platform using the *affy* package [110]. The GeneChip Robust Multiarray Average (*GCRMA*) algorithm was used for background correction, normalisation, and probe set summarisation [111]. The *arrayQualityMetrics* package was employed to generate microarray quality metrics reports [112]. The linear modelling of the microarray data and the identification of DEGs was performed with the *limma* package [113]. The *limma* computes t-statistics and log-odds of differential expression by the empirical Bayes shrinkage of the standard errors toward a common value. Probe sets having a *p*-value of < 0.05 and a 2-fold expression change were considered as differentially expressed under mineral stress treatments compared to the control. The gene model IDs for the probes were retrieved from the Gramene database [114] for subsequent analyses. Where the gene model IDs are not available, the Affymetrix probe IDs were used to mention the genes.

### 4.5. Gene Ontology, KEGG Enrichment and Gene-Regulatory Network (GRN) Analyses

Gene ontology (GO) and KEGG enrichment analyses were performed using ShinyGO v 0.61 [115]. The function categories of the genes were selected with the enrichment FDR (false discover rate) of *p* < 0.05. The transcription factors (TFs) regulating the expression of transporters (DEGs) were retrieved from the PlantRegMap database and the miRNAs regulating the expression of stress responsive genes at post-transcriptional level were predicted by the psRNATarget tool [116,117]. The complete Fe and Zn transporters regulatory network was realised using Cytoscape [118] and furthermore, the network was analysed to find important nodes (genes) that played a major role in the flow of information within the biological system and helping plants’ adaptation during Fe and Zn deficiencies.

### 4.6. Validation and Expression Correlation of DEGs

A total of 16 expression data points was validated using four genes through the qRT-PCR assay (Agilent Technologies, Santa Clara, CA, USA) (Table S5; Figure S2). The first-strand cDNA was synthesised from 250 ng of total RNA using an Affinity Script qRT-PCR cDNA synthesis kit (Stratagene, Agilent Technologies, Santa Clara, CA, USA). With the help of the IDT server [119], gene-specific primers were designed (Table S5). The qRT-PCR reaction was performed using Stratagene MX3005P (Agilent Technologies, Santa Clara, CA, USA) with the following PCR conditions: 10 min at 95 °C (preheating), followed by 40 cycles of amplification with denaturation for 30 s at 60 °C, primer annealing for 1 min at 58 °C and primer extension for 30 s at 72 °C [120,121].

## 5. Conclusions

The current investigation uncovered Fe and Zn-deficiency-responsive transcriptome signatures of transporters, phytohormone and carbohydrate metabolisms in maize. The Fe and Zn deficiencies altered the morpho-physiological and molecular responses through the differential expression of genes associated with phytohormonal regulations, transporters and photosynthesis. The result of the present investigation also revealed the dual regulation and anti-directional expression of key transporter genes suggesting something more than a direct/inverse association between Fe and Zn at transcriptome levels through regulatory proteins. Furthermore, the GRN analysis revealed the interactions among genes associated with hormone signalling and Fe and Zn transporters. The various DEGs regulating Fe and Zn homeostasis could be employed as candidate genes for enhancing Fe and Zn efficiency in maize through marker-assisted breeding, genetic engineering and genome editing approaches. Furthermore, this investigation sets the stage for the use of reverse genetic tools to analyse the stress-hormone signalling pathways and the interaction of various metabolic processes in the maize under Fe and Zn deficiencies and their interactions.

## Figures and Tables

**Table 1:** Differential expression of genes associated with the mugineic acid pathway, transporters, phytohormones, photosynthesis and carbohydrate metabolism in response to –Zn, –Fe and –Zn–Fe stresses in the root and shoot tissues. A minimum of a 2-fold change with *p* < 0.05 were considered for further analysis. The grey colour values indicate a non-significant *p-value*. Positive and negative values indicate increases and decreases in gene expression, respectively.

S. No.	Gene Models	Probe ID	Fold Change						Annotation
			Root			Shoot			
			–Zn	–Fe	–Fe–Zn	–Zn	–Fe	–Fe–Zn	
Transporters and Mugineic Acid Pathway									
1	GRMZM2G142870	Zm.17901.1.S1_at	2.09	1.18	15.46	1.93	−0.74	−1.21	ABC transporter C family member 14
2	GRMZM2G015295	Zm.10189.1.A1_at	2.10	−1.23	−9.12	1.25	7.51	30.81	Adenosylhomocysteinase
3	GRMZM5G843141	ZmAffx.1313.1.S1_s_at	1.13	2.65	−1.77	2.06	2.80	2.83	ATP synthase subunit alpha (atp1-a2)
4	GRMZM2G036908	Zm.15922.1.S1_at	−1.13	−2.38	10.63	1.53	−2.48	−6.57	Cation transmembrane transporter
5	GRMZM5G862882	Zm.18006.1.A1_at	2.14	3.71	7.69	2.03	−1.37	−6.30	Cation transmembrane transporter
6	GRMZM2G064023	Zm.5927.2.A1_a_at	3.04	1.03	1.92	−1.26	6.89	5.41	Citrate synthase 2
7	GRMZM2G157263	Zm.3964.1.A1_a_at	2.35	2.45	2.83	1.44	2.71	3.46	Ferric-chelate reductase
8	GRMZM2G123486	Zm.4621.1.A1_at	−4.04	−3.91	−3.58	1.17	−1.07	2.00	Heavy metal transport/detoxification superfamily protein
9	GRMZM2G178190	Zm.13647.1.S1_at	−1.23	5.07	7.22	−3.76	2.10	−3.66	Metal ion transmembrane transporter activity
10	GRMZM2G122437	Zm.6067.2.A1_at	−2.54	−2.33	−14.22	1.59	−1.34	9.14	Metal ion transporter
11	GRMZM2G099340	Zm.1356.2.S1_a_at	−1.40	−3.90	7.20	2.74	2.15	2.99	Metallothionein-like protein type 2
12	GRMZM2G131473	Zm.4989.1.A1_at	1.85	−1.05	1.48	−1.23	6.14	8.11	Methionine aminopeptidase
13	GRMZM2G015401	Zm.198.1.S1_at	2.78	−1.45	−1.93	1.08	3.46	10.28	Mitochondrial phosphate transporter
14	GRMZM2G034956	Zm.8336.1.S1_x_at	1.02	4.98	−1.29	1.06	−1.01	1.97	Nicotianamine synthase 1
15	GRMZM2G050108	Zm.9637.1.A1_at	2.07	−2.31	20.78	1.35	−1.18	−100.67	Nicotianamine synthase 3
16	GRMZM2G069198	Zm.12619.1.A1_at	3.15	1.56	7.28	2.34	3.38	1.67	NRAMP transporter1
17	GRMZM5G827496	Zm.17744.1.A1_at	−2.58	1.07	10.36	1.20	3.93	−14.55	NRT1/ PTR family 3.1
18	GRMZM2G567452	Zm.10825.1.A2_at	1.34	1.14	1.42	2.91	2.54	1.56	O-methyltransferase
19	GRMZM2G148800	Zm.19137.1.A1_at	−1.04	6.72	25.37	−48.72	−0.75	−126.24	Oligopeptide transmembrane transporter
20	GRMZM2G092125	Zm.605.1.S1_a_at	2.07	−1.85	−4.81	−1.02	7.21	3.86	Plasma membrane intrinsic protein 2
21	GRMZM2G014914	Zm.14436.1.A1_at	−1.70	−1.45	−1.61	1.37	18.02	8.85	Plasma membrane intrinsic protein 2
22	GRMZM2G099628	Zm.15800.5.A1_a_at	1.83	−2.40	−2.35	−1.06	15.83	26.41	Probable methionine-tRNA ligase
23	GRMZM2G104418	Zm.12750.2.A1_x_at	1.16	−1.67	−1.25	1.05	19.23	8.65	Proton-transporting V-type ATPase, V0 domain
24	GRMZM2G070605	Zm.14951.4.S1_at	1.99	2.26	2.77	−1.33	6.68	2.82	S-adenosylmethionine decarboxylase proenzyme
25	GRMZM2G054123	Zm.5785.1.S1_at	1.15	−1.17	−1.57	−1.32	3.92	9.42	S-adenosylmethionine synthetase 1
26	−	Zm.9197.1.A1_at	−2.23	−1.61	−2.50	−1.16	6.86	3.34	Tonoplast intrinsic protein 1
27	GRMZM2G027098	Zm.613.1.A1_at	−2.26	−6.54	−4.77	3.91	1.70	4.49	Tonoplast intrinsic protein 2
28	GRMZM2G056908	Zm.614.1.A1_at	−2.27	−9.93	−636.10	1.62	−1.29	120.97	Tonoplast intrinsic protein 2
29	GRMZM2G070360	Zm.3314.2.A1_a_at	1.91	1.44	2.89	1.18	21.50	6.22	V-type proton ATPase subunit E3
30	GRMZM2G094497	Zm.6859.1.A1_at	1.50	1.05	1.00	−1.23	9.84	2.00	Vacuolar ATP synthase subunit B
31	GRMZM2G421857	Zm.6945.1.A1_at	2.01	−1.22	7.50	−2.40	19.02	2.91	Vacuolar proton pump 3
32	GRMZM2G128995	Zm.4630.2.A1_a_at	1.48	−1.32	1.43	1.24	11.89	7.92	Vacuolar proton-transporting V-type ATPase, V1 domain
33	GRMZM2G126860	Zm.12751.1.S1_at	1.12	−1.15	1.93	−1.13	5.05	2.05	Vacuolar sorting protein 4b
34	GRMZM2G067546	Zm.3321.1.A1_a_at	1.33	1.23	4.31	1.89	8.57	4.03	Vacuolar sorting receptor homolog 1
35	GRMZM2G156599	Zm.582.1.S1_at	−2.12	8.55	35.45	−10.90	2.09	−38.26	Yellow stripe 1
36	GRMZM2G015955	Zm.13452.2.A1_at	1.64	1.26	10.59	3.62	1.19	1.39	Zinc transporter 4
37	GRMZM2G064382	Zm.18511.1.S1_at	5.53	1.15	3.85	8.72	-1.82	-4.10	ZRT-IRT-like protein 5
Phytohormonal Metabolism									
1	GRMZM2G074267	Zm.7858.1.A1_at	−1.25	−3.48	−5.35	1.26	−1.50	3.40	Auxin efflux carrier component PIN1bA: J
2	GRMZM2G427451	Zm.16735.1.A1_at	1.57	−1.96	−99.75	−1.30	4.24	112.22	Auxin induced in root cultures 12
3	GRMZM2G035465	Zm.2321.2.A1_a_at	2.08	1.32	9.13	3.87	1.03	−1.35	Auxin-responsive protein
4	GRMZM2G077356	Zm.4896.1.A1_at	1.30	−2.17	−9.12	2.59	1.30	9.07	Auxin-responsive Aux/IAA family member IAA 13
5	GRMZM2G147243	Zm.2321.3.A1_a_at	1.80	1.19	10.43	1.40	41.19	11.45	Auxin-responsive Aux/IAA family member IAA 17
6	GRMZM2G115354	Zm.7611.2.A1_at	2.06	−1.01	2.13	1.40	1.57	−1.17	Auxin-responsive Aux/IAA family member IAA 24
7	GRMZM2G141903	Zm.5181.2.A1_a_at	2.39	−1.23	5.46	1.15	10.64	6.42	CK2 protein kinase alpha 2
8	GRMZM2G050997	Zm.9754.1.A1_at	2.39	−1.12	21.32	−1.10	1.67	−32.91	Cytokinin oxidase 2
9	GRMZM2G167220	Zm.16971.1.A1_at	1.00	2.01	11.57	−3.13	−1.09	−23.68	Cytokinin oxidase 3
10	GRMZM2G167220	Zm.16971.1.A1_s_at	1.87	5.12	23.03	−2.88	−1.12	−35.98	Cytokinin oxidase 3
11	GRMZM2G041699	Zm.15533.1.S1_at	−1.54	−2.01	−1.93	−4.77	−2.15	−5.99	Cytokinin-O-glucosyltransferase 2
12	GRMZM2G065694	Zm.13139.1.A1_a_at	−1.11	−2.27	3.40	1.17	−1.58	−4.02	EREBP-4 like protein
13	GRMZM2G025062	Zm.10181.1.A1_at	−4.27	−8.86	−8.92	1.85	−1.23	−1.07	ERF-like protein
14	GRMZM2G102601	Zm.14806.1.A1_at	1.26	−2.12	1.43	1.46	5.50	3.43	Ethylene receptor
15	GRMZM2G052667	Zm.6775.1.A1_at	1.46	2.86	3.61	2.51	1.26	−2.48	Ethylene response factor
16	GRMZM2G053503	Zm.11441.1.S1_at	−1.43	−2.61	−4.86	−4.02	−4.16	−5.74	Ethylene-responsive factor-like protein 1
17	GRMZM5G831102	Zm.8468.1.A1_at	−1.35	−5.78	−8.20	5.00	−1.96	15.97	Gibberellin receptor GID1L2
18	GRMZM2G165901	Zm.2157.6.S1_x_at	−1.25	−1.50	−17.02	−1.34	13.02	8.67	Responsive to abscisic acid 15
Photosynthesis and Carbohydrate Metabolism									
1	GRMZM2G448174	ZmAffx.1483.1.S1_at	1.81	1.28	−3.47	−1.27	15.00	2.92	Apocytochrome f precursor
2	GRMZM2G385635	ZmAffx.1092.1.A1_at	−1.47	4.19	−2.34	1.29	2.32	4.10	ATPase beta chain
3	GRMZM2G336448	Zm.19239.1.A1_at	1.13	4.22	2.12	−4.79	−1.33	−8.81	Carbohydrate transporter/sugar porter/transporter
4	GRMZM2G029219	Zm.3331.1.A1_at	−1.49	−2.28	24.02	2.81	5.37	−2.63	Carbohydrate transporter/sugar porter/transporter
5	GRMZM2G121878	Zm.1079.1.A1_a_at	6.24	1.16	804.18	3.14	13.05	−129.47	Carbonic anhydrase
6	GRMZM2G424832	Zm.520.1.S1_x_at	−1.49	−2.11	−4.06	3.91	−1.66	2.04	Cellulose synthase 4
7	GRMZM2G018241	Zm.523.1.S1_at	−1.46	−2.08	−9.33	2.24	−1.28	3.77	Cellulose synthase 9
8	GRMZM2G142898	ZmAffx.5.1.S1_at	1.11	−1.77	−7.57	2.07	−1.84	2.85	Cellulose synthase catalytic subunit 12
9	GRMZM2G047513	Zm.7289.1.A1_at	1.00	1.14	5.87	−1.57	−4.78	−32.62	Chloroplast 30S ribosomal protein S10
10	−	ZmAffx.1219.1.S1_s_at	6.83	2.12	−2.13	9.49	7.00	18.67	Chlorophyll a-b binding protein 6A
11	GRMZM2G145460	Zm.15915.1.S1_at	−1.10	−1.42	−2.38	−1.78	−7.22	−5.92	Chloroplast SRP54 receptor 1
12	GRMZM2G064302	Zm.14043.3.S1_a_at	5.18	2.35	−2.88	1.48	46.52	35.20	Enolase 1
13	GRMZM2G048371	Zm.410.1.A1_at	1.39	−1.02	−2.32	−2.00	7.79	24.38	Enolase 2
14	GRMZM2G053458	Zm.4.1.S1_at	1.35	1.71	−2.32	−1.21	25.82	17.88	Ferredoxin 3
15	GRMZM2G106190	Zm.136.1.S1_at	−1.24	−8.66	−2.28	3.47	−1.50	3.73	Ferredoxin 6, chloroplastic
16	GRMZM2G113325	Zm.1691.1.S1_a_at	−1.38	2.35	20.44	1.12	1.11	−9.52	Ferrochelatase
17	GRMZM2G051004	Zm.8992.2.A1_a_at	2.86	−1.56	−4.31	1.06	3.41	34.02	Glyceraldehyde-3-phosphate dehydrogenase
18	GRMZM2G415359	Zm.5727.1.S1_a_at	2.37	−1.01	1.36	2.37	21.14	15.47	Malate dehydrogenase 5
19	GRMZM2G142873	Zm.8962.1.A1_at	2.55	−1.15	3.49	1.40	4.29	1.20	Opaque endosperm 5
20	GRMZM2G065083	Zm.3318.1.S1_at	1.42	−2.86	1.79	−1.06	15.22	2.71	Phosphohexose isomerase 1
21	GRMZM2G382914	Zm.6780.1.A1_a_at	−1.14	−1.79	−1.06	−1.22	104.09	36.76	Phosphoglycerate kinase
22	GRMZM2G382914	Zm.6780.3.A1_x_at	−1.25	−1.41	−1.07	−1.34	25.90	11.82	Phosphoglycerate kinase
23	GRMZM2G076276	Zm.15933.1.A1_at	1.89	−1.29	−1.18	3.89	1.21	7.50	Predicted polygalacturonate 4-alpha-galacturonosyltransferase
24	GRMZM2G165357	Zm.730.3.A1_x_at	1.70	−1.53	−4.90	1.49	3.50	11.60	Predicted UDP-glucuronic acid decarboxylase 1
25	GRMZM2G059151	Zm.2422.1.A1_at	1.17	−1.49	−2.16	2.07	−1.53	3.21	Pyrophosphate--fructose 6-phosphate 1-phosphotransferase subunit beta
26	GRMZM2G113033	Zm.6763.4.S1_a_at	1.56	2.39	−1.09	1.87	520.30	27.36	Ribulose bisphosphate carboxylase small subunit 2
27	GRMZM2G095252	Zm.3461.1.A1_a_at	1.32	−1.19	1.14	−1.15	104.80	8.52	RuBisCO large subunit-binding protein subunit beta
28	GRMZM5G875238	Zm.26.1.A1_at	−1.24	2.96	308.21	9.08	3.65	−31.29	Sucrose phosphate synthase 1
29	GRMZM2G140107	Zm.18317.1.A1_at	1.32	2.44	68.25	−1.20	2.11	−80.91	Sucrose-phosphate synthase-like
30	GRMZM2G134256	Zm.2199.1.A1_a_at	2.15	−1.08	2.35	2.03	5.68	3.10	Transaldolase 2
31	GRMZM2G030784	Zm.3889.1.A1_at	1.91	1.38	−1.22	1.36	6.97	10.31	Triosephosphate isomerase
32	GRMZM2G073725	Zm.752.1.S1_a_at	2.15	−1.47	−2.33	−1.25	25.38	23.42	UDP-arabinopyranose mutase 3
33	GRMZM2G328500	Zm.4986.1.S1_at	1.14	-3.00	−4.23	−1.80	5.47	11.70	UDP-glucose 6-dehydrogenase
34	GRMZM5G862540	Zm.4986.2.S1_at	1.09	−2.44	−5.64	−1.40	3.00	4.10	UDP-glucose 6-dehydrogenase
35	GRMZM2G042179	Zm.12322.1.A1_at	−1.17	−3.17	−1.08	2.18	2.30	3.02	UDP-glucuronic acid 4-epimerase
36	GRMZM2G067707	Zm.4793.1.S1_a_at	2.99	1.35	1.49	1.32	22.03	24.99	Ubiquinol-cytochrome c reductase complex
37	GRMZM5G833389	Zm.5548.1.A1_x_at	2.37	−1.32	−6.95	−1.04	12.38	13.28	2,3-bisphosphoglycerate-independent phosphoglycerate mutase

## Data Availability

The raw microarray expression data files of the present study have been deposited in GEO (Accession No. GSE122581; Sample No. GSM3474993 to GSM3475016). All other supporting data are included as supplementary files.

## Supplementary Materials

The following are available online at https://www.mdpi.com/2223-7747/9/12/1812/s1, Figure S1. Initial and final chlorophyll content in the leaves of SKV616 maize inbred under complete (+Fe+Zn) hydroponic solution (*significant at *p* < 0.5); Figure S2. Correlation coefficients among morpho-physiological parameters under Fe and Zn stresses in maize. (CC: chlorophyll content (SPAD-value); PR: photosynthesis rate; TR: transpiration rate; SC: stomatal conductance; Fv/Fm: quantum efficiency of PS II photochemistry; and RL: Root length; **, *** significant at *p* < 0.01 and *p* < 0.001, respectively); Figure S3. Heatmap of differentially expressed transporters and mugineic acid pathways genes. The z-scores are computed for all genes that are differentially expressed with *p* < 0.05 and > 2-fold expression; Figure S4. Heatmap of differentially expressed phytohormonal metabolism genes. The z-scores are computed for all genes that are differentially expressed with *p* < 0.05 and > 2-fold expression; Figure S5. Heatmap of differentially expressed photosynthesis and carbohydrate metabolism genes. The z-scores are computed for all genes that are differentially expressed with *p* < 0.05 and > 2-fold expression; Table S1. Gene ontology terms for differentially expressed genes in the root and shoot under the –Zn, –Fe and –Fe–Zn treatments; Table S2. KEGG-enriched functional categories of differentially expressed genes in the root and shoot under –Zn, –Fe and –Fe–Zn stress treatments; Table S3. The predicted miRNA–gene and TF–gene interactions used to construct the gene regulatory network for the transporter and mugineic acid pathway genes; Table S4. The GRN features of the transporter and mugineic acid pathway genes; Table S5. Primer* sequences of DEGs selected from microarray analyses for qRT-PCR validation; Figure S6. Validation of DEGs from microarray analysis through qRT-PCR. The x axis represents the probes and the y axis represents the fold change expression values of genes. Error bars in the column represent the standard error. The letters ‘S’ and ‘R’ in the stress name on the x axis refer to the shoot and root, respectively. The reactions were performed using the Stratagene MX3005P (Agilent Technologies, Santa Clara, California, USA) Real-Time PCR system with the following PCR conditions: 10 min, at 95 °C (preheating), followed by 40 cycles of amplification with denaturation for 30 s at 60 °C, primer annealing for 1 min at 58 °C and primer extension for 30 s at 72 °C.
