# Supplementary material for: Comparative Transcriptome Analysis of Iron and Zinc Deficiency in Maize (Zea mays L.)
Source: Plants (Basel). 2020 Dec 21;9(12):1812. doi: 10.3390/plants9121812 (PMC7767415; doi:10.3390/plants9121812)
Supplement: Supplementary file 1 [file plants-09-01812-s001.pdf]

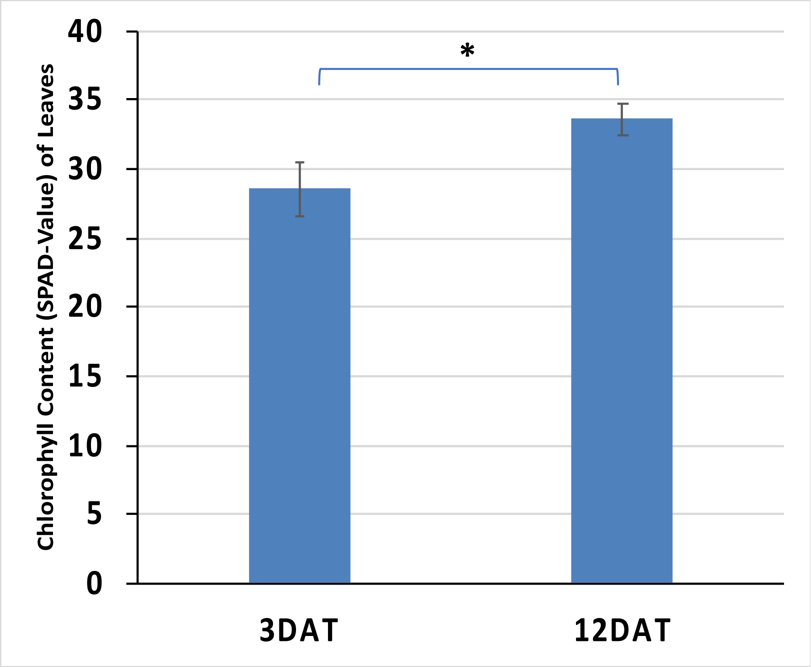

Figure S1. Initial and final chlorophyll content in the leaves of SKV616 maize inbred under complete (+Fe+Zn) hydroponic solution (*significant at $p < 0.5$).

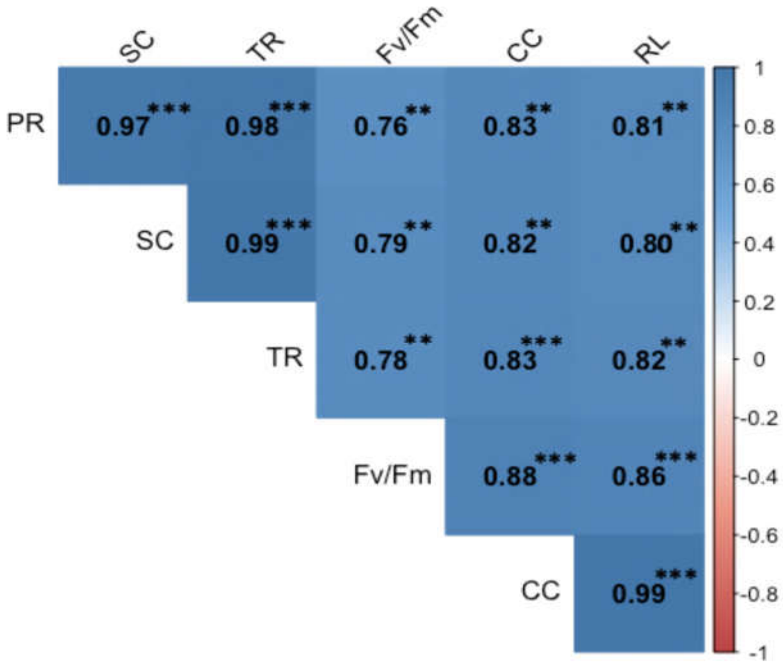

Figure S2. Correlation coefficients among morpho-physiological parameters under Fe and Zn stresses in maize. (CC: chlorophyll content (SPAD-value); PR: photosynthesis rate; TR: transpiration rate; SC: stomatal conductance; Fv/Fm: quantum efficiency of PS II photochemistry; and RL: Root length; **, *** significant at $p < 0.01$ and $p < 0.001$, respectively).

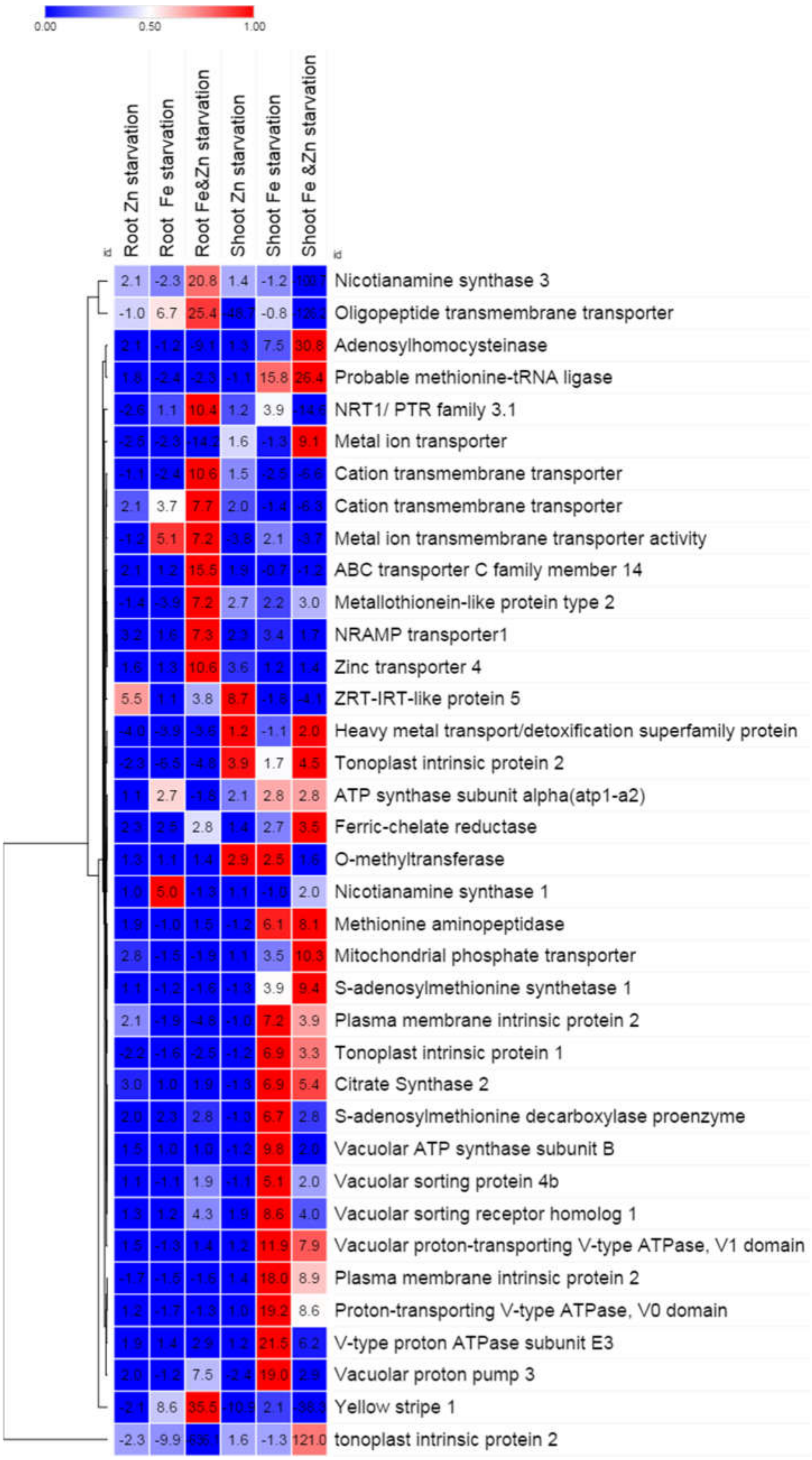

Figure S3. Heatmap of differentially expressed transporters and mugineic acid pathways genes. The z-scores are computed for all genes that are differentially expressed with $p < 0.05$ and > 2-fold expression.

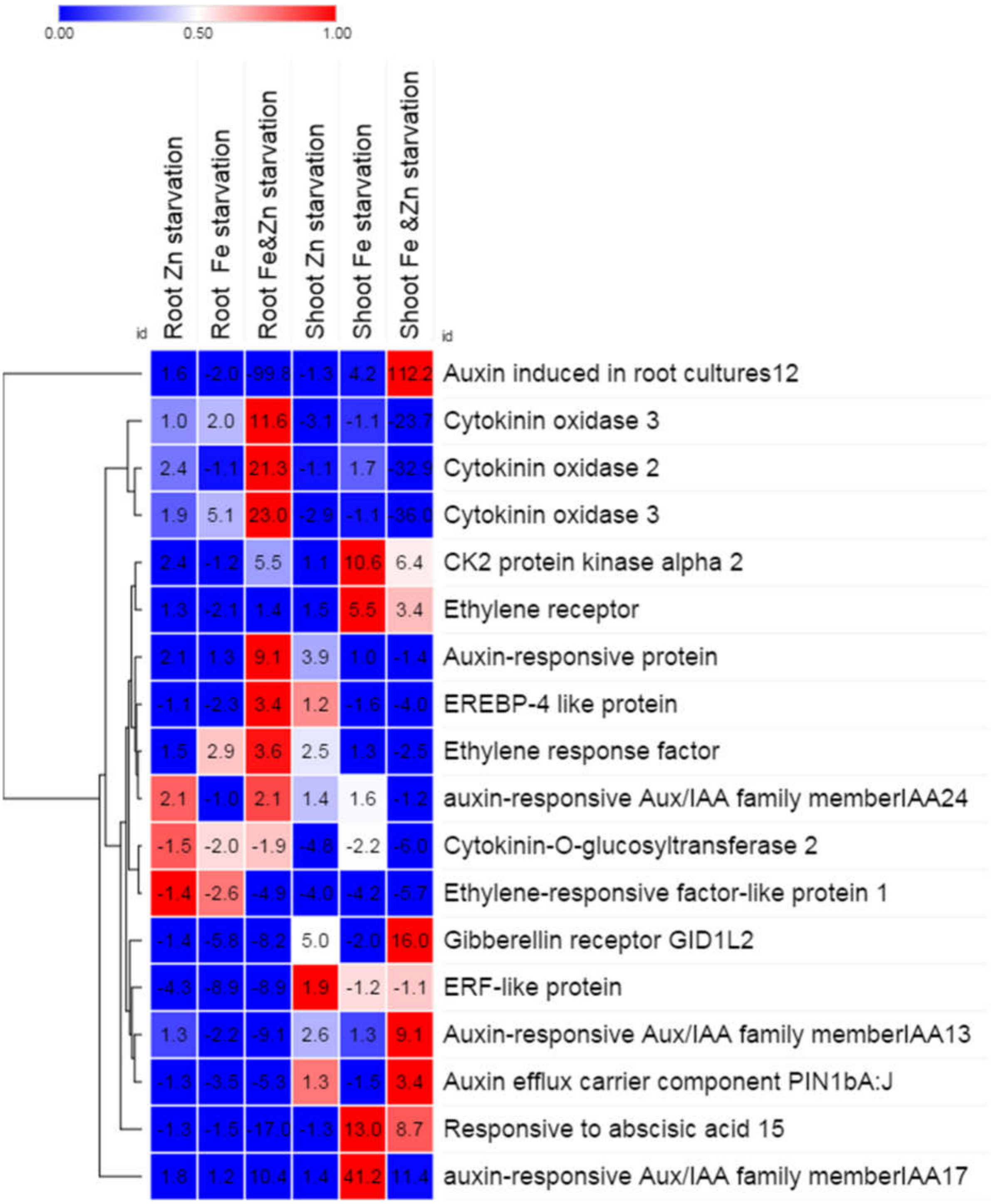

Figure S4. Heatmap of differentially expressed phytohormonal metabolism genes. The z-scores are computed for all genes that are differentially expressed with $p < 0.05$ and > 2-fold expression.

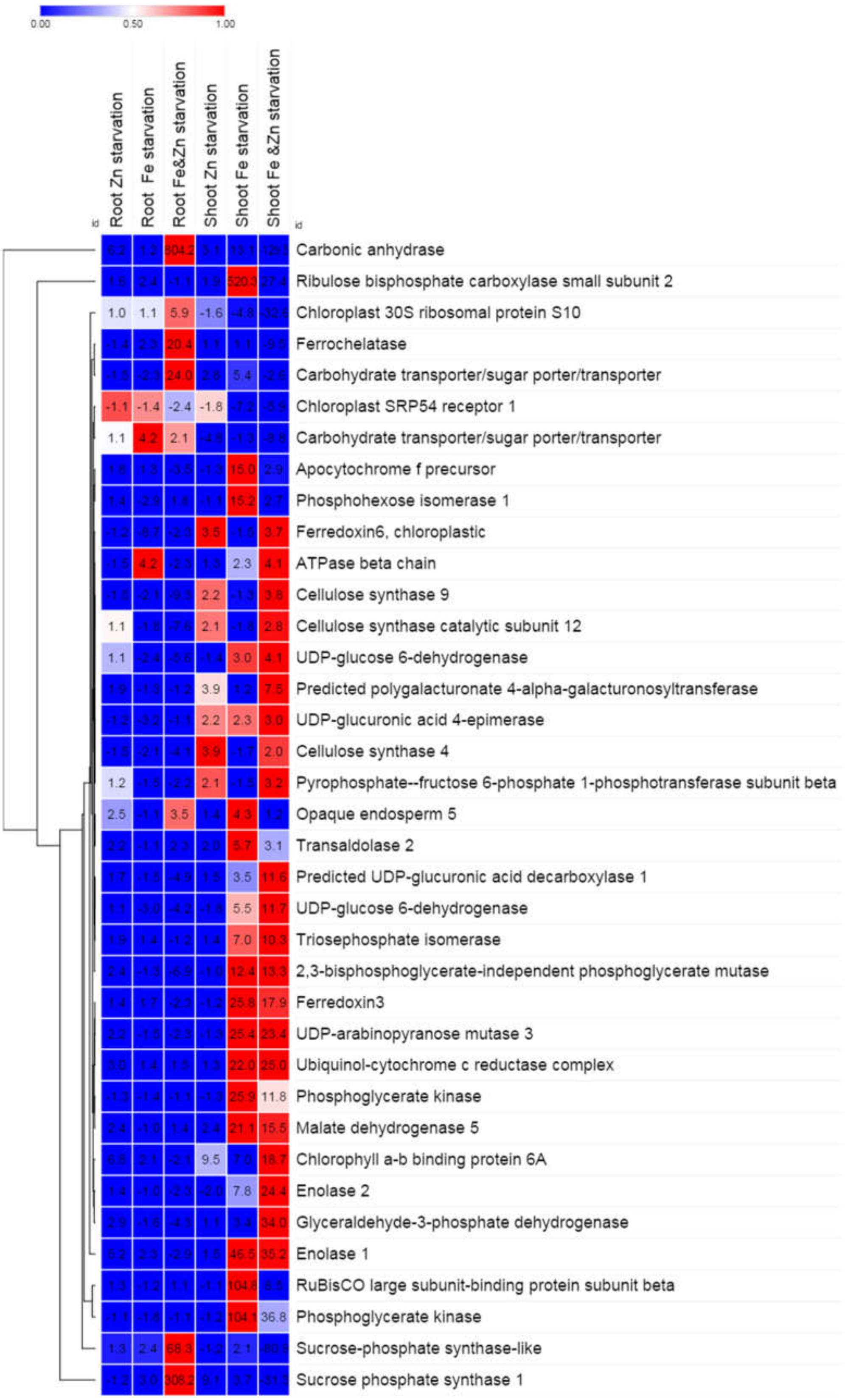

Figure S5. Heatmap of differentially expressed photosynthesis and carbohydrate metabolism genes. The z-scores are computed for all genes that are differentially expressed with $p < 0.05$ and > 2-fold expression.

Table S1. Gene ontology terms for differentially expressed genes in the root and shoot under the –Zn, –Fe and –Fe–Zn treatments.

Tissue	Stress	Enrichment FDR	Genes in List	Total Genes	GO Terms Category	GO category
Root	–Zn	2.80E-04	138	3894	Response to stimulus	Biological process
Root	–Zn	3.30E-06	95	2098	Response to stress	Biological process
Root	–Zn	5.20E-04	90	2324	Oxidation–reduction process	Biological process
Root	–Zn	5.00E-04	81	2021	Small molecule metabolic process	Biological process
Root	–Zn	2.80E-04	67	1534	Response to chemical	Biological process
Root	–Zn	5.00E-04	39	743	Response to abiotic stimulus	Biological process
Root	–Zn	7.20E-04	39	766	Co-factor metabolic process	Biological process
Root	–Zn	2.00E-03	34	675	Response to oxygen-containing compound	Biological process
Root	–Zn	2.80E-04	25	359	Response to toxic substance	Biological process
Root	–Zn	2.00E-03	23	376	Defence response	Biological process
Root	–Zn	4.10E-04	22	299	Antibiotic metabolic process	Biological process
Root	–Zn	2.00E-03	22	351	Response to oxidative stress	Biological process
Root	–Zn	8.90E-04	20	279	Cellular oxidant detoxification	Biological process
Root	–Zn	1.60E-03	20	292	Cellular detoxification	Biological process
Root	–Zn	2.10E-04	19	208	Reactive oxygen species metabolic process	Biological process
Root	–Zn	6.80E-04	16	184	Antibiotic catabolic process	Biological process
Root	–Zn	6.80E-04	15	163	Hydrogen peroxide catabolic process	Biological process
Root	–Zn	7.20E-04	15	166	Hydrogen peroxide metabolic process	Biological process
Root	–Zn	2.00E-03	14	166	Cellular modified amino acid metabolic process	Biological process
Root	–Zn	2.80E-04	12	91	Glutathione metabolic process	Biological process
Root	–Zn	2.30E-03	11	109	Response to water deprivation	Biological process
Root	–Zn	2.30E-03	11	109	Response to water	Biological process
Root	–Zn	2.50E-04	8	35	Fluid transport	Biological process
Root	–Zn	2.50E-04	8	35	Water transport	Biological process
Root	–Zn	6.80E-04	4	7	AMP biosynthetic process	Biological process

Root	−Zn	1.70E-03	4	9	AMP metabolic process	Biological process
Root	−Zn	1.40E-03	156	4812	Organelle part	Cellular component
Root	−Zn	1.80E-03	155	4810	Intracellular organelle part	Cellular component
Root	−Zn	4.70E-03	135	4205	Protein-containing complex	Cellular component
Root	−Zn	3.30E-06	78	1675	Cytosol	Cellular component
Root	−Zn	1.20E-02	71	2052	Non-membrane-bounded organelle	Cellular component
Root	−Zn	1.20E-02	71	2052	Intracellular non-membrane-bounded organelle	Cellular component
Root	−Zn	1.40E-03	51	1191	Membrane-enclosed lumen	Cellular component
Root	−Zn	1.40E-03	51	1191	Organelle lumen	Cellular component
Root	−Zn	1.40E-03	51	1191	Intracellular organelle lumen	Cellular component
Root	−Zn	3.60E-07	45	680	Extracellular region	Cellular component
Root	−Zn	2.60E-04	29	468	Mitochondrial part	Cellular component
Root	−Zn	1.40E-03	24	398	Cell wall	Cellular component
Root	−Zn	1.40E-03	24	399	External encapsulating structure	Cellular component
Root	−Zn	6.80E-03	19	333	Nucleolus	Cellular component
Root	−Zn	1.20E-02	17	311	Mitochondrial envelope	Cellular component
Root	−Zn	1.50E-04	16	161	Apoplast	Cellular component
Root	−Zn	8.20E-03	16	267	Mitochondrial protein complex	Cellular component
Root	−Zn	3.40E-03	13	167	Protein–DNA complex	Cellular component
Root	−Zn	6.90E-03	13	184	Plant-type cell wall	Cellular component
Root	−Zn	1.30E-03	12	117	Photosystem	Cellular component
Root	−Zn	1.00E-02	11	151	Mitochondrial matrix	Cellular component
Root	−Zn	9.70E-03	10	127	DNA packaging complex	Cellular component
Root	−Zn	1.40E-03	8	58	Photosystem I	Cellular component
Root	−Zn	1.20E-02	8	90	Photosystem II	Cellular component
Root	−Zn	2.90E-03	6	35	Preribosome, large subunit precursor	Cellular component
Root	−Zn	6.90E-03	4	17	Mitochondrial intermembrane space	Cellular component
Root	−Zn	6.90E-03	4	17	Organelle envelope lumen	Cellular component
Root	−Zn	3.80E-03	3	6	Protein storage vacuole membrane	Cellular component
Root	−Zn	7.20E-03	3	8	Storage vacuole	Cellular component

Root	−Zn	7.20E-03	3	8	Protein storage vacuole	Cellular component
Root	−Zn	3.60E-04	118	3261	Metal ion binding	Molecular function
Root	−Zn	4.60E-04	118	3288	Cation binding	Molecular function
Root	−Zn	3.60E-04	85	2134	Oxidoreductase activity	Molecular function
Root	−Zn	3.60E-04	65	1495	Co-factor binding	Molecular function
Root	−Zn	1.40E-03	33	634	Tetrapyrrole binding	Molecular function
Root	−Zn	3.60E-04	31	524	Hydrolase activity, acting on glycosyl bonds	Molecular function
Root	−Zn	5.30E-04	28	469	Hydrolase activity, hydrolysing O-glycosyl compounds	Molecular function
Root	−Zn	1.50E-02	24	483	Isomerase activity	Molecular function
Root	−Zn	5.30E-04	20	272	Antioxidant activity	Molecular function
Root	−Zn	1.60E-03	17	231	Peroxidase activity	Molecular function
Root	−Zn	1.60E-03	17	231	Oxidoreductase activity, acting on peroxide as acceptor	Molecular function
Root	−Zn	2.50E-03	15	195	Transferase activity, transferring alkyl or aryl (other than methyl) groups	Molecular function
Root	−Zn	9.50E-05	12	75	Glutathione transferase activity	Molecular function
Root	−Zn	2.20E-02	11	157	Substrate-specific channel activity	Molecular function
Root	−Zn	1.30E-02	10	119	RRNA binding	Molecular function
Root	−Zn	1.20E-04	8	33	Water transmembrane transporter activity	Molecular function
Root	−Zn	1.20E-04	8	33	Water channel activity	Molecular function
Root	−Zn	3.90E-03	7	48	Chlorophyll binding	Molecular function
Root	−Zn	6.20E-03	7	53	Sulphur compound binding	Molecular function
Root	−Zn	4.40E-03	6	35	Xyloglucan:xyloglucosyl transferase activity	Molecular function
Root	−Zn	4.90E-03	6	36	Chitinase activity	Molecular function
Root	−Zn	2.00E-02	6	50	Carbohydrate phosphatase activity	Molecular function
Root	−Zn	1.80E-02	5	32	Modified amino acid binding	Molecular function
Root	−Zn	2.00E-02	5	34	Trehalose-phosphatase activity	Molecular function
Root	−Zn	2.00E-02	4	20	Glutathione binding	Molecular function
Root	−Zn	2.00E-02	4	20	Oligopeptide binding	Molecular function

Root	−Zn	1.30E-02	2	2	Arsenite transmembrane transporter activity	Molecular function
Root	−Zn	2.20E-02	2	3	Adenosine kinase activity	Molecular function
Root	−Zn	2.20E-02	2	3	Pyruvate decarboxylase activity	Molecular function
Root	−Zn	2.20E-02	2	3	Linoleate 13S-lipoxygenase activity	Molecular function
Root	−Fe	8.40E-08	216	3894	Response to stimulus	Biological process
Root	−Fe	2.80E-07	142	2324	Oxidation–reduction process	Biological process
Root	−Fe	3.90E-06	125	2098	Response to stress	Biological process
Root	−Fe	1.10E-09	113	1534	Response to chemical	Biological process
Root	−Fe	1.80E-11	107	1316	Carbohydrate metabolic process	Biological process
Root	−Fe	6.90E-07	69	893	Cellular response to chemical stimulus	Biological process
Root	−Fe	2.40E-07	64	766	Co-factor metabolic process	Biological process
Root	−Fe	1.20E-06	60	743	Response to abiotic stimulus	Biological process
Root	−Fe	2.60E-06	55	675	Response to oxygen-containing compound	Biological process
Root	−Fe	2.00E-08	53	527	Cell wall organization or biogenesis	Biological process
Root	−Fe	3.60E-06	46	523	Cellular carbohydrate metabolic process	Biological process
Root	−Fe	1.10E-05	43	499	Polysaccharide metabolic process	Biological process
Root	−Fe	3.30E-07	40	375	External encapsulating structure organization	Biological process
Root	−Fe	6.90E-07	38	359	Cell wall organization	Biological process
Root	−Fe	1.50E-06	37	359	Response to toxic substance	Biological process
Root	−Fe	9.80E-07	33	289	Glucan metabolic process	Biological process
Root	−Fe	2.40E-06	33	304	Cytoskeleton organization	Biological process
Root	−Fe	9.50E-06	33	327	Microtubule-based process	Biological process
Root	−Fe	1.10E-05	33	331	Cellular polysaccharide metabolic process	Biological process
Root	−Fe	1.40E-06	32	281	Cellular glucan metabolic process	Biological process
Root	−Fe	4.70E-06	31	285	Drug catabolic process	Biological process
Root	−Fe	6.90E-07	27	200	Cell wall biogenesis	Biological process
Root	−Fe	1.30E-06	27	208	Reactive oxygen species metabolic process	Biological process
Root	−Fe	6.50E-06	27	230	Plant-type cell wall organization or biogenesis	Biological process
Root	−Fe	8.10E-06	22	163	Hydrogen peroxide catabolic process	Biological process
Root	−Fe	1.00E-05	22	166	Hydrogen peroxide metabolic process	Biological process

Root	–Fe	9.70E-06	16	91	Glutathione metabolic process	Biological process
Root	–Fe	5.50E-07	14	52	Sterol biosynthetic process	Biological process
Root	–Fe	9.50E-09	159	2582	Cell periphery	Cellular component
Root	–Fe	7.30E-08	122	1914	Endomembrane system	Cellular component
Root	–Fe	2.40E-05	119	2096	Plasma membrane	Cellular component
Root	–Fe	4.20E-05	74	1162	Organelle subcompartment	Cellular component
Root	–Fe	8.20E-12	69	680	Extracellular region	Cellular component
Root	–Fe	5.30E-06	67	947	Golgi apparatus	Cellular component
Root	–Fe	4.60E-05	58	843	Endoplasmic reticulum	Cellular component
Root	–Fe	2.80E-05	43	540	Golgi apparatus part	Cellular component
Root	–Fe	1.10E-05	40	464	Golgi subcompartment	Cellular component
Root	–Fe	1.30E-04	40	527	Cell-cell junction	Cellular component
Root	–Fe	1.30E-04	40	527	Plasmodesma	Cellular component
Root	–Fe	1.30E-04	40	527	Cell junction	Cellular component
Root	–Fe	1.30E-04	40	527	Symplast	Cellular component
Root	–Fe	2.00E-07	38	363	Cytoskeleton	Cellular component
Root	–Fe	4.60E-05	38	464	Plasma membrane part	Cellular component
Root	–Fe	5.30E-06	37	398	Cell wall	Cellular component
Root	–Fe	5.30E-06	37	399	External encapsulating structure	Cellular component
Root	–Fe	2.00E-07	35	318	Cytoskeletal part	Cellular component
Root	–Fe	2.00E-07	31	259	Microtubule cytoskeleton	Cellular component
Root	–Fe	3.50E-04	31	385	Intrinsic component of plasma membrane	Cellular component
Root	–Fe	5.00E-08	30	225	Supramolecular complex	Cellular component
Root	–Fe	5.00E-08	30	225	Supramolecular polymer	Cellular component
Root	–Fe	5.00E-08	30	225	Supramolecular fibre	Cellular component
Root	–Fe	5.00E-08	30	225	Polymeric cytoskeletal fibre	Cellular component
Root	–Fe	1.80E-07	28	213	Microtubule	Cellular component
Root	–Fe	2.80E-05	22	189	Anchored component of plasma membrane	Cellular component
Root	–Fe	3.50E-05	22	193	Anchored component of membrane	Cellular component
Root	–Fe	3.00E-04	22	226	Trans-Golgi network	Cellular component

Root	−Fe	2.80E-05	20	161	Apoplast	Cellular component
Root	−Fe	2.40E-04	4	6	Protein storage vacuole membrane	Cellular component
Root	−Fe	9.80E-06	178	3288	Cation binding	Molecular function
Root	−Fe	1.20E-05	176	3261	Metal ion binding	Molecular function
Root	−Fe	2.70E-06	129	2134	Oxidoreductase activity	Molecular function
Root	−Fe	3.10E-04	87	1495	Co-factor binding	Molecular function
Root	−Fe	2.00E-04	61	928	Transferase activity, transferring glycosyl groups	Molecular function
Root	−Fe	5.30E-08	52	524	Hydrolase activity, acting on glycosyl bonds	Molecular function
Root	−Fe	4.60E-07	46	469	Hydrolase activity, hydrolysing O-glycosyl compounds	Molecular function
Root	−Fe	3.40E-04	45	629	Transferase activity, transferring hexosyl groups	Molecular function
Root	−Fe	4.20E-04	37	483	Isomerase activity	Molecular function
Root	−Fe	1.70E-05	35	370	Nucleoside binding	Molecular function
Root	−Fe	1.70E-05	35	369	Ribonucleoside binding	Molecular function
Root	−Fe	1.70E-05	34	355	Purine nucleoside binding	Molecular function
Root	−Fe	1.70E-05	34	353	GTP binding	Molecular function
Root	−Fe	1.70E-05	34	355	Guanyl nucleotide binding	Molecular function
Root	−Fe	1.70E-05	34	355	Purine ribonucleoside binding	Molecular function
Root	−Fe	1.70E-05	34	355	Guanyl ribonucleotide binding	Molecular function
Root	−Fe	2.10E-05	29	280	GTPase activity	Molecular function
Root	−Fe	2.20E-05	28	266	Glucosyltransferase activity	Molecular function
Root	−Fe	7.90E-05	27	272	Antioxidant activity	Molecular function
Root	−Fe	4.50E-05	25	231	Peroxidase activity	Molecular function
Root	−Fe	4.50E-05	25	231	Oxidoreductase activity, acting on peroxide as acceptor	Molecular function
Root	−Fe	6.00E-04	20	195	Transferase activity, transferring alkyl or aryl (other than methyl) groups	Molecular function
Root	−Fe	4.90E-04	16	132	Amide binding	Molecular function
Root	−Fe	2.00E-05	14	75	Glutathione transferase activity	Molecular function
Root	−Fe	1.20E-04	11	56	Structural constituent of cytoskeleton	Molecular function

Root	−Fe	2.00E-04	10	49	Cellulose synthase (UDP-forming) activity	Molecular function
Root	−Fe	2.30E-04	10	50	Cellulose synthase activity	Molecular function
Root	−Fe	5.40E-05	9	33	Water transmembrane transporter activity	Molecular function
Root	−Fe	5.40E-05	9	33	Water channel activity	Molecular function
Root	−Fe	8.40E-05	9	35	Xyloglucan:xyloglucosyl transferase activity	Molecular function
Root	−Fe−Zn	3.80E-25	903	4736	Cellular nitrogen compound biosynthetic process	Biological process
Root	−Fe−Zn	3.70E-20	742	3894	Response to stimulus	Biological process
Root	−Fe−Zn	7.00E-18	689	3642	Cellular component organization or biogenesis	Biological process
Root	−Fe−Zn	2.10E-65	636	2354	Organonitrogen compound biosynthetic process	Biological process
Root	−Fe−Zn	1.10E-38	498	2021	Small molecule metabolic process	Biological process
Root	−Fe−Zn	6.40E-17	470	2324	Oxidation–reduction process	Biological process
Root	−Fe−Zn	2.40E-60	452	1496	Cellular amide metabolic process	Biological process
Root	−Fe−Zn	6.00E-59	414	1331	Peptide metabolic process	Biological process
Root	−Fe−Zn	6.90E-55	399	1304	Amide biosynthetic process	Biological process
Root	−Fe−Zn	8.80E-56	382	1214	Peptide biosynthetic process	Biological process
Root	−Fe−Zn	1.40E-56	380	1196	Translation	Biological process
Root	−Fe−Zn	1.40E-25	370	1545	Cellular component biogenesis	Biological process
Root	−Fe−Zn	5.70E-24	362	1534	Response to chemical	Biological process
Root	−Fe−Zn	1.70E-24	300	1188	Oxoacid metabolic process	Biological process
Root	−Fe−Zn	2.10E-24	300	1190	Organic acid metabolic process	Biological process
Root	−Fe−Zn	4.40E-25	295	1152	Carboxylic acid metabolic process	Biological process
Root	−Fe−Zn	3.40E-19	244	977	Cellular component assembly	Biological process
Root	−Fe−Zn	3.40E-16	225	928	Protein-containing complex subunit organization	Biological process
Root	−Fe−Zn	1.50E-25	216	743	Response to abiotic stimulus	Biological process
Root	−Fe−Zn	7.20E-19	207	788	Protein-containing complex assembly	Biological process
Root	−Fe−Zn	6.40E-18	200	766	Co-factor metabolic process	Biological process
Root	−Fe−Zn	6.00E-18	183	678	Generation of precursor metabolites and energy	Biological process
Root	−Fe−Zn	7.40E-16	183	710	Cellular protein-containing complex assembly	Biological process
Root	−Fe−Zn	4.30E-21	181	627	Ribonucleoprotein complex biogenesis	Biological process

Root	–Fe–Zn	1.40E-16	173	647	Nucleobase-containing small molecule metabolic process	Biological process
Root	–Fe–Zn	2.50E-19	149	493	Ribosome biogenesis	Biological process
Root	–Fe–Zn	3.30E-16	92	264	Ribonucleoprotein complex subunit organization	Biological process
Root	–Fe–Zn	2.40E-17	57	118	Ribosome assembly	Biological process
Root	–Fe–Zn	4.10E-111	1210	4812	Organelle part	Cellular component
Root	–Fe–Zn	4.10E-111	1209	4810	Intracellular organelle part	Cellular component
Root	–Fe–Zn	2.60E-72	991	4205	Protein-containing complex	Cellular component
Root	–Fe–Zn	2.20E-76	599	2052	Non-membrane-bounded organelle	Cellular component
Root	–Fe–Zn	2.20E-76	599	2052	Intracellular non-membrane-bounded organelle	Cellular component
Root	–Fe–Zn	2.90E-77	501	1570	Plastid	Cellular component
Root	–Fe–Zn	9.20E-66	498	1675	Cytosol	Cellular component
Root	–Fe–Zn	1.30E-65	436	1381	Ribonucleoprotein complex	Cellular component
Root	–Fe–Zn	4.90E-68	303	776	Ribosome	Cellular component
Root	–Fe–Zn	1.20E-54	289	815	Plastid part	Cellular component
Root	–Fe–Zn	2.10E-64	214	456	Ribosomal subunit	Cellular component
Root	–Fe–Zn	3.80E-27	211	714	Organelle envelope	Cellular component
Root	–Fe–Zn	3.80E-27	211	714	Envelope	Cellular component
Root	–Fe–Zn	2.70E-56	201	449	Cytosolic part	Cellular component
Root	–Fe–Zn	6.30E-49	185	429	Thylakoid	Cellular component
Root	–Fe–Zn	5.50E-55	184	394	Cytosolic ribosome	Cellular component
Root	–Fe–Zn	6.80E-35	145	356	Thylakoid part	Cellular component
Root	–Fe–Zn	6.40E-60	143	240	Large ribosomal subunit	Cellular component
Root	–Fe–Zn	2.60E-34	141	344	Photosynthetic membrane	Cellular component
Root	–Fe–Zn	3.80E-27	139	385	Plastid stroma	Cellular component
Root	–Fe–Zn	2.90E-34	137	329	Plastid thylakoid	Cellular component
Root	–Fe–Zn	1.20E-32	134	327	Thylakoid membrane	Cellular component
Root	–Fe–Zn	7.40E-55	123	197	Cytosolic large ribosomal subunit	Cellular component
Root	–Fe–Zn	4.30E-30	120	288	Plastid thylakoid membrane	Cellular component
Root	–Fe–Zn	7.10E-21	106	293	Plastid envelope	Cellular component

Root	−Fe−Zn	8.50E-10	775	4542	Small molecule binding	Molecular function
Root	−Fe−Zn	8.50E-10	773	4529	Anion binding	Molecular function
Root	−Fe−Zn	4.30E-07	710	4264	Nucleotide binding	Molecular function
Root	−Fe−Zn	4.30E-07	710	4264	Nucleoside phosphate binding	Molecular function
Root	−Fe−Zn	2.80E-14	615	3288	Cation binding	Molecular function
Root	−Fe−Zn	6.20E-14	608	3261	Metal ion binding	Molecular function
Root	−Fe−Zn	4.30E-05	606	3699	Purine ribonucleoside triphosphate binding	Molecular function
Root	−Fe−Zn	2.40E-12	417	2134	Oxidoreductase activity	Molecular function
Root	−Fe−Zn	1.00E-33	403	1589	RNA binding	Molecular function
Root	−Fe−Zn	6.70E-14	318	1495	Co-factor binding	Molecular function
Root	−Fe−Zn	1.00E-61	313	855	Structural molecule activity	Molecular function
Root	−Fe−Zn	4.70E-69	279	667	Structural constituent of ribosome	Molecular function
Root	−Fe−Zn	6.70E-12	160	649	Coenzyme binding	Molecular function
Root	−Fe−Zn	1.40E-05	115	529	Lyase activity	Molecular function
Root	−Fe−Zn	1.40E-10	102	370	Nucleoside binding	Molecular function
Root	−Fe−Zn	1.40E-10	102	369	Ribonucleoside binding	Molecular function
Root	−Fe−Zn	1.40E-10	99	355	Purine nucleoside binding	Molecular function
Root	−Fe−Zn	1.40E-10	99	355	Guanyl nucleotide binding	Molecular function
Root	−Fe−Zn	1.40E-10	99	355	Purine ribonucleoside binding	Molecular function
Root	−Fe−Zn	1.40E-10	99	355	Guanyl ribonucleotide binding	Molecular function
Root	−Fe−Zn	2.30E-10	98	353	GTP binding	Molecular function
Root	−Fe−Zn	6.80E-07	74	280	GTPase activity	Molecular function
Root	−Fe−Zn	4.20E-07	68	246	Unfolded protein binding	Molecular function
Root	−Fe−Zn	1.90E-07	63	217	MRNA binding	Molecular function
Root	−Fe−Zn	2.80E-05	63	248	Oxidoreductase activity, acting on the CH–OH group of donors, NAD or NADP as acceptor	Molecular function
Root	−Fe−Zn	2.40E-12	62	165	Protein heterodimerization activity	Molecular function
Root	−Fe−Zn	6.20E-14	53	119	RRNA binding	Molecular function
Root	−Fe−Zn	2.70E-05	40	132	Identical protein binding	Molecular function
Root	−Fe−Zn	2.90E-06	38	113	NAD binding	Molecular function

Shoot	–Zn	2.30E-09	175	3894	Response to stimulus	Biological process
Shoot	–Zn	2.30E-19	148	2324	Oxidation–reduction process	Biological process
Shoot	–Zn	5.70E-08	106	2098	Response to stress	Biological process
Shoot	–Zn	1.90E-08	105	2021	Small molecule metabolic process	Biological process
Shoot	–Zn	2.40E-09	89	1534	Response to chemical	Biological process
Shoot	–Zn	5.70E-07	73	1316	Carbohydrate metabolic process	Biological process
Shoot	–Zn	1.60E-05	63	1188	Oxoacid metabolic process	Biological process
Shoot	–Zn	1.60E-05	63	1190	Organic acid metabolic process	Biological process
Shoot	–Zn	8.20E-12	61	743	Response to abiotic stimulus	Biological process
Shoot	–Zn	1.90E-10	55	678	Generation of precursor metabolites and energy	Biological process
Shoot	–Zn	1.60E-07	52	766	Co-factor metabolic process	Biological process
Shoot	–Zn	3.10E-06	38	523	Cellular carbohydrate metabolic process	Biological process
Shoot	–Zn	1.00E-09	37	359	Response to toxic substance	Biological process
Shoot	–Zn	2.00E-13	36	251	Photosynthesis	Biological process
Shoot	–Zn	2.20E-08	34	351	Response to oxidative stress	Biological process
Shoot	–Zn	5.70E-07	29	307	Response to inorganic substance	Biological process
Shoot	–Zn	7.50E-06	26	292	Cellular response to toxic substance	Biological process
Shoot	–Zn	7.50E-06	26	292	Cellular detoxification	Biological process
Shoot	–Zn	1.10E-05	26	301	Detoxification	Biological process
Shoot	–Zn	1.10E-06	25	245	Response to temperature stimulus	Biological process
Shoot	–Zn	1.00E-05	25	279	Cellular oxidant detoxification	Biological process
Shoot	–Zn	1.80E-05	22	235	Response to external biotic stimulus	Biological process
Shoot	–Zn	1.80E-05	22	235	Response to other organism	Biological process
Shoot	–Zn	1.10E-05	21	208	Reactive oxygen species metabolic process	Biological process
Shoot	–Zn	1.10E-07	17	98	Response to reactive oxygen species	Biological process
Shoot	–Zn	1.70E-05	16	129	Photosynthesis, light reaction	Biological process
Shoot	–Zn	7.50E-06	13	77	Response to antibiotic	Biological process
Shoot	–Zn	6.10E-07	12	51	Protein-chromophore linkage	Biological process
Shoot	–Zn	1.10E-05	12	68	Cellular response to oxidative stress	Biological process
Shoot	–Zn	1.00E-05	10	44	Response to hydrogen peroxide	Biological process

Shoot	−Zn	1.10E-23	122	1570	Plastid	Cellular component
Shoot	−Zn	4.60E-24	121	1521	Chloroplast	Cellular component
Shoot	−Zn	2.00E-02	78	2096	Plasma membrane	Cellular component
Shoot	−Zn	1.70E-10	74	1162	Organelle subcompartment	Cellular component
Shoot	−Zn	1.20E-14	67	801	Chloroplast part	Cellular component
Shoot	−Zn	2.40E-14	67	815	Plastid part	Cellular component
Shoot	−Zn	2.50E-02	64	1675	Cytosol	Cellular component
Shoot	−Zn	2.60E-21	57	429	Thylakoid	Cellular component
Shoot	−Zn	5.40E-16	45	356	Thylakoid part	Cellular component
Shoot	−Zn	6.30E-16	44	344	Photosynthetic membrane	Cellular component
Shoot	−Zn	1.20E-14	41	329	Chloroplast thylakoid	Cellular component
Shoot	−Zn	1.20E-14	41	329	Plastid thylakoid	Cellular component
Shoot	−Zn	1.90E-13	39	327	Thylakoid membrane	Cellular component
Shoot	−Zn	2.80E-04	39	714	Organelle envelope	Cellular component
Shoot	−Zn	2.80E-04	39	714	Envelope	Cellular component
Shoot	−Zn	9.20E-14	37	288	Chloroplast thylakoid membrane	Cellular component
Shoot	−Zn	9.20E-14	37	288	Plastid thylakoid membrane	Cellular component
Shoot	−Zn	1.40E-02	32	680	Extracellular region	Cellular component
Shoot	−Zn	2.00E-05	28	379	Chloroplast stroma	Cellular component
Shoot	−Zn	2.60E-05	28	385	Plastid stroma	Cellular component
Shoot	−Zn	2.60E-07	27	285	Chloroplast envelope	Cellular component
Shoot	−Zn	4.40E-07	27	293	Plastid envelope	Cellular component
Shoot	−Zn	9.00E-11	21	117	Photosystem	Cellular component
Shoot	−Zn	2.90E-02	20	385	Intrinsic component of plasma membrane	Cellular component
Shoot	−Zn	2.00E-12	17	58	Photosystem I	Cellular component
Shoot	−Zn	1.00E-02	15	226	Trans-Golgi network	Cellular component
Shoot	−Zn	5.20E-05	12	90	Photosystem II	Cellular component
Shoot	−Zn	1.40E-05	10	53	Plastoglobule	Cellular component
Shoot	−Zn	4.20E-03	10	104	Extracellular space	Cellular component
Shoot	−Zn	5.90E-03	10	109	Extracellular region part	Cellular component

Shoot	−Zn	5.40E-10	156	3288	Cation binding	Molecular function
Shoot	−Zn	6.50E-10	154	3261	Metal ion binding	Molecular function
Shoot	−Zn	1.10E-16	133	2134	Oxidoreductase activity	Molecular function
Shoot	−Zn	5.10E-13	97	1495	Co-factor binding	Molecular function
Shoot	−Zn	4.00E-03	74	1799	Transporter activity	Molecular function
Shoot	−Zn	6.50E-10	51	634	Tetrapyrrole binding	Molecular function
Shoot	−Zn	5.90E-04	37	649	Coenzyme binding	Molecular function
Shoot	−Zn	2.30E-04	36	585	Heme binding	Molecular function
Shoot	−Zn	1.10E-04	23	272	Antioxidant activity	Molecular function
Shoot	−Zn	2.30E-04	17	173	Magnesium ion binding	Molecular function
Shoot	−Zn	3.60E-03	17	231	Peroxidase activity	Molecular function
Shoot	−Zn	3.60E-03	17	231	Oxidoreductase activity, acting on peroxide as acceptor	Molecular function
Shoot	−Zn	1.50E-09	14	48	Chlorophyll binding	Molecular function
Shoot	−Zn	2.90E-03	12	124	Carbohydrate transmembrane transporter activity	Molecular function
Shoot	−Zn	1.40E-03	11	96	Sugar transmembrane transporter activity	Molecular function
Shoot	−Zn	4.70E-03	10	98	Oxidoreductase activity, acting on the aldehyde or oxo group of donors	Molecular function
Shoot	−Zn	3.60E-03	9	75	Glutathione transferase activity	Molecular function
Shoot	−Zn	4.00E-03	9	78	Oxidoreductase activity, acting on the aldehyde or oxo group of donors, NAD or NADP as acceptor	Molecular function
Shoot	−Zn	1.00E-03	8	48	Molecular carrier activity	Molecular function
Shoot	−Zn	4.70E-03	7	49	Cellulose synthase (UDP-forming) activity	Molecular function
Shoot	−Zn	1.80E-04	5	10	Ferric iron binding	Molecular function
Shoot	−Zn	2.30E-04	5	11	Metallochaperone activity	Molecular function
Shoot	−Zn	4.00E-03	5	22	Ferrous iron binding	Molecular function
Shoot	−Zn	2.80E-04	4	6	Superoxide dismutase copper chaperone activity	Molecular function
Shoot	−Zn	1.40E-03	4	9	Copper chaperone activity	Molecular function
Shoot	−Zn	5.80E-04	3	3	Oxygen carrier activity	Molecular function
Shoot	−Zn	5.80E-04	3	3	Linoleate 13S-lipoxygenase activity	Molecular function

Shoot	−Zn	5.80E-04	3	3	Oxygen binding	Molecular function
Shoot	−Zn	1.60E-03	3	4	Ferroxidase activity	Molecular function
Shoot	−Zn	1.60E-03	3	4	Oxidoreductase activity, oxidizing metal ions, oxygen as acceptor	Molecular function
Shoot	−Fe	1.20E-30	376	2354	Organonitrogen compound biosynthetic process	Biological process
Shoot	−Fe	2.60E-42	368	2021	Small molecule metabolic process	Biological process
Shoot	−Fe	1.70E-16	322	2324	Oxidation–reduction process	Biological process
Shoot	−Fe	5.60E-18	235	1496	Cellular amide metabolic process	Biological process
Shoot	−Fe	4.20E-25	219	1190	Organic acid metabolic process	Biological process
Shoot	−Fe	6.70E-25	218	1188	Oxoacid metabolic process	Biological process
Shoot	−Fe	9.70E-26	216	1152	Carboxylic acid metabolic process	Biological process
Shoot	−Fe	1.60E-16	211	1331	Peptide metabolic process	Biological process
Shoot	−Fe	2.00E-15	204	1304	Amide biosynthetic process	Biological process
Shoot	−Fe	1.60E-13	187	1214	Peptide biosynthetic process	Biological process
Shoot	−Fe	1.60E-13	185	1196	Translation	Biological process
Shoot	−Fe	9.40E-17	154	855	Small molecule biosynthetic process	Biological process
Shoot	−Fe	1.50E-15	153	875	Organophosphate metabolic process	Biological process
Shoot	−Fe	4.30E-14	151	893	Carbohydrate derivative metabolic process	Biological process
Shoot	−Fe	1.70E-18	144	743	Response to abiotic stimulus	Biological process
Shoot	−Fe	3.50E-16	141	766	Co-factor metabolic process	Biological process
Shoot	−Fe	5.60E-21	137	647	Nucleobase-containing small molecule metabolic process	Biological process
Shoot	−Fe	3.50E-19	118	540	Nucleoside phosphate metabolic process	Biological process
Shoot	−Fe	5.10E-19	116	529	Nucleotide metabolic process	Biological process
Shoot	−Fe	6.40E-13	105	555	Cellular amino acid metabolic process	Biological process
Shoot	−Fe	5.10E-14	89	416	Ribose phosphate metabolic process	Biological process
Shoot	−Fe	4.70E-14	86	394	Coenzyme metabolic process	Biological process
Shoot	−Fe	4.10E-14	79	344	Nucleotide biosynthetic process	Biological process
Shoot	−Fe	7.90E-14	79	349	Nucleoside phosphate biosynthetic process	Biological process
Shoot	−Fe	9.10E-21	76	251	Photosynthesis	Biological process

Shoot	–Fe	1.40E-12	76	347	Co-factor biosynthetic process	Biological process
Shoot	–Fe	5.50E-13	55	203	Oxidoreduction coenzyme metabolic process	Biological process
Shoot	–Fe	1.80E-13	54	192	Pyridine-containing compound metabolic process	Biological process
Shoot	–Fe	4.30E-45	692	4812	Organelle part	Cellular component
Shoot	–Fe	4.30E-45	692	4810	Intracellular organelle part	Cellular component
Shoot	–Fe	5.40E-32	581	4205	Protein-containing complex	Cellular component
Shoot	–Fe	5.10E-79	380	1570	Plastid	Cellular component
Shoot	–Fe	1.30E-80	376	1521	Chloroplast	Cellular component
Shoot	–Fe	1.70E-13	277	2052	Non-membrane-bounded organelle	Cellular component
Shoot	–Fe	1.70E-13	277	2052	Intracellular non-membrane-bounded organelle	Cellular component
Shoot	–Fe	1.80E-25	276	1675	Cytosol	Cellular component
Shoot	–Fe	1.50E-54	222	815	Plastid part	Cellular component
Shoot	–Fe	1.40E-54	220	801	Chloroplast part	Cellular component
Shoot	–Fe	7.10E-17	215	1381	Ribonucleoprotein complex	Cellular component
Shoot	–Fe	8.90E-18	192	1162	Organelle subcompartment	Cellular component
Shoot	–Fe	2.10E-19	140	714	Organelle envelope	Cellular component
Shoot	–Fe	2.10E-19	140	714	Envelope	Cellular component
Shoot	–Fe	7.40E-42	137	429	Thylakoid	Cellular component
Shoot	–Fe	4.30E-12	131	795	Membrane protein complex	Cellular component
Shoot	–Fe	1.40E-10	124	776	Ribosome	Cellular component
Shoot	–Fe	2.60E-28	109	385	Plastid stroma	Cellular component
Shoot	–Fe	7.60E-31	108	356	Thylakoid part	Cellular component
Shoot	–Fe	9.90E-28	107	379	Chloroplast stroma	Cellular component
Shoot	–Fe	2.80E-30	105	344	Photosynthetic membrane	Cellular component
Shoot	–Fe	2.40E-31	104	329	Chloroplast thylakoid	Cellular component
Shoot	–Fe	2.40E-31	104	329	Plastid thylakoid	Cellular component
Shoot	–Fe	2.80E-28	99	327	Thylakoid membrane	Cellular component
Shoot	–Fe	3.20E-26	89	288	Chloroplast thylakoid membrane	Cellular component
Shoot	–Fe	3.20E-26	89	288	Plastid thylakoid membrane	Cellular component
Shoot	–Fe	1.80E-20	81	293	Plastid envelope	Cellular component

Shoot	–Fe	1.20E-20	80	285	Chloroplast envelope	Cellular component
Shoot	–Fe	1.80E-11	37	117	Photosystem	Cellular component
Shoot	–Fe	1.20E-08	22	58	Photosystem I	Cellular component
Shoot	–Fe	9.70E-08	499	4542	Small molecule binding	Molecular function
Shoot	–Fe	4.50E-07	492	4529	Anion binding	Molecular function
Shoot	–Fe	1.90E-05	453	4264	Nucleotide binding	Molecular function
Shoot	–Fe	1.90E-05	453	4264	Nucleoside phosphate binding	Molecular function
Shoot	–Fe	5.00E-11	398	3288	Cation binding	Molecular function
Shoot	–Fe	1.10E-10	393	3261	Metal ion binding	Molecular function
Shoot	–Fe	2.00E-12	286	2134	Oxidoreductase activity	Molecular function
Shoot	–Fe	2.90E-14	235	1589	RNA binding	Molecular function
Shoot	–Fe	2.90E-14	224	1495	Co-factor binding	Molecular function
Shoot	–Fe	1.20E-07	126	855	Structural molecule activity	Molecular function
Shoot	–Fe	7.30E-14	121	649	Coenzyme binding	Molecular function
Shoot	–Fe	2.50E-09	110	667	Structural constituent of ribosome	Molecular function
Shoot	–Fe	2.10E-05	80	529	Lyase activity	Molecular function
Shoot	–Fe	3.40E-04	70	483	Isomerase activity	Molecular function
Shoot	–Fe	2.10E-07	51	246	Unfolded protein binding	Molecular function
Shoot	–Fe	1.10E-04	44	248	Oxidoreductase activity, acting on the CH–OH group of donors, NAD or NADP as acceptor	Molecular function
Shoot	–Fe	9.50E-07	38	165	Protein heterodimerization activity	Molecular function
Shoot	–Fe	3.40E-04	36	195	Transferase activity, transferring alkyl or aryl (other than methyl) groups	Molecular function
Shoot	–Fe	3.60E-08	35	127	Oxidoreductase activity, acting on NAD(P)H	Molecular function
Shoot	–Fe	3.40E-04	33	173	Magnesium ion binding	Molecular function
Shoot	–Fe	9.50E-07	30	113	NAD binding	Molecular function
Shoot	–Fe	6.80E-04	23	105	Hydro-lyase activity	Molecular function
Shoot	–Fe	2.10E-05	21	73	Ubiquitin-like protein conjugating enzyme activity	Molecular function
Shoot	–Fe	2.10E-05	20	67	Ubiquitin conjugating enzyme activity	Molecular function
Shoot	–Fe	3.70E-04	20	81	Intramolecular oxidoreductase activity	Molecular function

Shoot	−Fe	3.40E-04	19	74	Heat shock protein binding	Molecular function
Shoot	−Fe	6.20E-06	18	51	Oxidoreductase activity, acting on NAD(P)H, quinone or similar compound as acceptor	Molecular function
Shoot	−Fe	1.20E-05	17	48	Chlorophyll binding	Molecular function
Shoot	−Fe	5.80E-05	15	43	NADH dehydrogenase activity	Molecular function
Shoot	−Fe	3.40E-04	11	28	Misfolded protein binding	Molecular function
Shoot	−Fe−Zn	7.10E-31	893	4736	Cellular nitrogen compound biosynthetic process	Biological process
Shoot	−Fe−Zn	6.60E-19	835	4772	Macromolecule biosynthetic process	Biological process
Shoot	−Fe−Zn	4.80E-21	715	3894	Response to stimulus	Biological process
Shoot	−Fe−Zn	1.50E-23	689	3642	Cellular component organization or biogenesis	Biological process
Shoot	−Fe−Zn	2.80E-82	654	2354	Organonitrogen compound biosynthetic process	Biological process
Shoot	−Fe−Zn	1.00E-75	469	1496	Cellular amide metabolic process	Biological process
Shoot	−Fe−Zn	2.00E-30	454	2021	Small molecule metabolic process	Biological process
Shoot	−Fe−Zn	1.10E-74	432	1331	Peptide metabolic process	Biological process
Shoot	−Fe−Zn	1.50E-72	422	1304	Amide biosynthetic process	Biological process
Shoot	−Fe−Zn	3.70E-70	398	1214	Peptide biosynthetic process	Biological process
Shoot	−Fe−Zn	1.50E-70	395	1196	Translation	Biological process
Shoot	−Fe−Zn	9.80E-33	378	1545	Cellular component biogenesis	Biological process
Shoot	−Fe−Zn	1.00E-20	337	1534	Response to chemical	Biological process
Shoot	−Fe−Zn	8.20E-26	254	977	Cellular component assembly	Biological process
Shoot	−Fe−Zn	2.20E-24	241	928	Protein-containing complex subunit organization	Biological process
Shoot	−Fe−Zn	5.90E-20	232	949	RNA processing	Biological process
Shoot	−Fe−Zn	4.70E-27	221	788	Protein-containing complex assembly	Biological process
Shoot	−Fe−Zn	4.20E-30	219	743	Response to abiotic stimulus	Biological process
Shoot	−Fe−Zn	7.30E-20	198	766	Co-factor metabolic process	Biological process
Shoot	−Fe−Zn	1.90E-30	196	627	Ribonucleoprotein complex biogenesis	Biological process
Shoot	−Fe−Zn	2.60E-22	194	710	Cellular protein-containing complex assembly	Biological process
Shoot	−Fe−Zn	8.80E-23	189	678	Generation of precursor metabolites and energy	Biological process
Shoot	−Fe−Zn	2.00E-22	182	647	Nucleobase-containing small molecule metabolic process	Biological process

Shoot	−Fe−Zn	8.20E-26	158	493	Ribosome biogenesis	Biological process
Shoot	−Fe−Zn	4.40E-20	155	540	Nucleoside phosphate metabolic process	Biological process
Shoot	−Fe−Zn	1.40E-20	154	529	Nucleotide metabolic process	Biological process
Shoot	−Fe−Zn	8.10E-20	129	416	Ribose phosphate metabolic process	Biological process
Shoot	−Fe−Zn	1.20E-21	99	264	Ribonucleoprotein complex subunit organization	Biological process
Shoot	−Fe−Zn	1.10E-21	96	251	Photosynthesis	Biological process
Shoot	−Fe−Zn	1.40E-20	95	255	Ribonucleoprotein complex assembly	Biological process
Shoot	−Fe−Zn	1.90E-134	1224	4812	Organelle part	Cellular component
Shoot	−Fe−Zn	4.80E-134	1222	4810	Intracellular organelle part	Cellular component
Shoot	−Fe−Zn	5.70E-130	1104	4205	Protein-containing complex	Cellular component
Shoot	−Fe−Zn	3.60E-92	613	2052	Non-membrane-bounded organelle	Cellular component
Shoot	−Fe−Zn	3.60E-92	613	2052	Intracellular non-membrane-bounded organelle	Cellular component
Shoot	−Fe−Zn	1.60E-81	515	1675	Cytosol	Cellular component
Shoot	−Fe−Zn	7.00E-98	483	1381	Ribonucleoprotein complex	Cellular component
Shoot	−Fe−Zn	1.10E-61	452	1570	Plastid	Cellular component
Shoot	−Fe−Zn	2.10E-62	444	1521	Chloroplast	Cellular component
Shoot	−Fe−Zn	5.80E-78	310	776	Ribosome	Cellular component
Shoot	−Fe−Zn	8.60E-32	307	1191	Membrane-enclosed lumen	Cellular component
Shoot	−Fe−Zn	8.60E-32	307	1191	Organelle lumen	Cellular component
Shoot	−Fe−Zn	2.70E-48	269	815	Plastid part	Cellular component
Shoot	−Fe−Zn	2.70E-48	266	801	Chloroplast part	Cellular component
Shoot	−Fe−Zn	5.30E-33	217	714	Organelle envelope	Cellular component
Shoot	−Fe−Zn	5.30E-33	217	714	Envelope	Cellular component
Shoot	−Fe−Zn	5.20E-66	211	456	Ribosomal subunit	Cellular component
Shoot	−Fe−Zn	3.50E-62	204	449	Cytosolic part	Cellular component
Shoot	−Fe−Zn	2.10E-58	184	394	Cytosolic ribosome	Cellular component
Shoot	−Fe−Zn	2.30E-47	178	429	Thylakoid	Cellular component
Shoot	−Fe−Zn	2.10E-32	163	468	Mitochondrial part	Cellular component
Shoot	−Fe−Zn	8.50E-37	144	356	Thylakoid part	Cellular component
Shoot	−Fe−Zn	1.90E-37	142	344	Photosynthetic membrane	Cellular component

Shoot	–Fe–Zn	7.00E-60	140	240	Large ribosomal subunit	Cellular component
Shoot	–Fe–Zn	1.20E-35	135	327	Thylakoid membrane	Cellular component
Shoot	–Fe–Zn	4.80E-34	133	329	Chloroplast thylakoid	Cellular component
Shoot	–Fe–Zn	4.80E-34	133	329	Plastid thylakoid	Cellular component
Shoot	–Fe–Zn	2.80E-34	123	288	Chloroplast thylakoid membrane	Cellular component
Shoot	–Fe–Zn	2.80E-34	123	288	Plastid thylakoid membrane	Cellular component
Shoot	–Fe–Zn	3.10E-54	120	197	Cytosolic large ribosomal subunit	Cellular component
Shoot	–Fe–Zn	5.10E-07	717	4542	Small molecule binding	Molecular function
Shoot	–Fe–Zn	5.10E-07	715	4529	Anion binding	Molecular function
Shoot	–Fe–Zn	1.50E-14	590	3288	Cation binding	Molecular function
Shoot	–Fe–Zn	1.50E-14	586	3261	Metal ion binding	Molecular function
Shoot	–Fe–Zn	1.10E-54	442	1589	RNA binding	Molecular function
Shoot	–Fe–Zn	3.60E-10	387	2134	Oxidoreductase activity	Molecular function
Shoot	–Fe–Zn	5.00E-67	313	855	Structural molecule activity	Molecular function
Shoot	–Fe–Zn	4.10E-11	293	1495	Co-factor binding	Molecular function
Shoot	–Fe–Zn	2.10E-73	278	667	Structural constituent of ribosome	Molecular function
Shoot	–Fe–Zn	3.70E-07	137	649	Coenzyme binding	Molecular function
Shoot	–Fe–Zn	4.60E-11	100	369	Ribonucleoside binding	Molecular function
Shoot	–Fe–Zn	5.10E-11	100	370	Nucleoside binding	Molecular function
Shoot	–Fe–Zn	1.10E-10	96	355	Purine nucleoside binding	Molecular function
Shoot	–Fe–Zn	1.10E-10	96	355	Guanyl nucleotide binding	Molecular function
Shoot	–Fe–Zn	1.10E-10	96	355	Purine ribonucleoside binding	Molecular function
Shoot	–Fe–Zn	1.10E-10	96	355	Guanyl ribonucleotide binding	Molecular function
Shoot	–Fe–Zn	1.80E-10	95	353	GTP binding	Molecular function
Shoot	–Fe–Zn	1.30E-13	81	246	Unfolded protein binding	Molecular function
Shoot	–Fe–Zn	2.00E-07	73	280	GTPase activity	Molecular function
Shoot	–Fe–Zn	7.60E-12	71	217	MRNA binding	Molecular function
Shoot	–Fe–Zn	7.70E-06	70	290	Translation factor activity, RNA binding	Molecular function
Shoot	–Fe–Zn	2.60E-06	67	266	Protein-containing complex binding	Molecular function
Shoot	–Fe–Zn	9.60E-13	61	165	Protein heterodimerization activity	Molecular function

Shoot	−Fe−Zn	7.80E-13	50	119	RRNA binding	Molecular function
Shoot	−Fe−Zn	6.80E-09	45	127	Oxidoreductase activity, acting on NAD(P)H	Molecular function
Shoot	−Fe−Zn	2.30E-06	37	113	NAD binding	Molecular function
Shoot	−Fe−Zn	4.30E-08	24	48	Chlorophyll binding	Molecular function
Shoot	−Fe−Zn	2.60E-06	21	46	Single-stranded RNA binding	Molecular function
Shoot	−Fe−Zn	7.40E-07	18	33	Water transmembrane transporter activity	Molecular function
Shoot	−Fe−Zn	7.40E-07	18	33	Water channel activity	Molecular function

Table S2. KEGG-enriched functional categories of differentially expressed genes in the root and shoot under –Zn, –Fe and –Fe–Zn stress treatments.

Tissue	Stress	Enrichment FDR	Genes in List	Total Genes	Functional Category
Root	–Zn	1.56E-11	85	1489	Metabolic pathways
Root	–Zn	3.22E-06	12	70	Glutathione metabolism
Root	–Zn	3.43E-06	46	824	Biosynthesis of secondary metabolites
Root	–Zn	6.24E-03	13	178	Biosynthesis of amino acids
Root	–Zn	7.60E-03	6	44	Alanine, aspartate and glutamate metabolism
Root	–Zn	1.47E-02	4	21	Photosynthesis
Root	–Zn	1.77E-02	4	24	Arginine biosynthesis
Root	–Zn	1.77E-02	3	12	Linoleic acid metabolism
Root	–Zn	1.77E-02	12	191	Carbon metabolism
Root	–Zn	3.24E-02	6	69	Purine metabolism
Root	–Zn	3.24E-02	8	111	Starch and sucrose metabolism
Root	–Zn	3.24E-02	5	48	Glyoxylate and dicarboxylate metabolism
Root	–Zn	3.55E-02	5	51	Citrate cycle (TCA cycle)
Root	–Zn	3.64E-02	6	73	Cysteine and methionine metabolism
Root	–Zn	4.79E-02	4	37	Phenylalanine, tyrosine and tryptophan biosynthesis
Root	–Fe	4.47E-16	125	1489	Metabolic pathways
Root	–Fe	1.86E-10	73	824	Biosynthesis of secondary metabolites
Root	–Fe	5.68E-06	14	70	Glutathione metabolism
Root	–Fe	3.94E-05	13	72	Phagosome
Root	–Fe	3.95E-04	14	103	Amino sugar and nucleotide sugar metabolism
Root	–Fe	7.36E-04	9	48	Glyoxylate and dicarboxylate metabolism
Root	–Fe	9.48E-04	10	63	Pyruvate metabolism
Root	–Fe	9.48E-04	19	191	Carbon metabolism
Root	–Fe	1.44E-03	7	33	Valine, leucine and isoleucine degradation

Root	–Fe	1.44E-03	8	44	DNA replication
Root	–Fe	6.45E-03	14	144	Phenylpropanoid biosynthesis
Root	–Fe	8.92E-03	11	102	Glycolysis/gluconeogenesis
Root	–Fe	1.01E-02	16	188	Protein processing in endoplasmic reticulum
Root	–Fe	1.12E-02	5	25	Steroid biosynthesis
Root	–Fe	1.22E-02	7	50	Pyrimidine metabolism
Root	–Fe	1.32E-02	11	111	Starch and sucrose metabolism
Root	–Fe	1.79E-02	8	69	Purine metabolism
Root	–Fe	2.26E-02	5	31	Ascorbate and aldarate metabolism
Root	–Fe	2.26E-02	8	73	Cysteine and methionine metabolism
Root	–Fe	2.52E-02	14	178	Biosynthesis of amino acids
Root	–Fe	2.58E-02	3	11	AGE-RAGE signalling pathway in diabetic complications
Root	–Fe	4.02E-02	4	24	Lysine degradation
Root	–Fe–Zn	6.01E-85	177	267	Ribosome
Root	–Fe–Zn	1.38E-52	430	1489	Metabolic pathways
Root	–Fe–Zn	2.64E-28	237	824	Biosynthesis of secondary metabolites
Root	–Fe–Zn	2.32E-16	74	191	Carbon metabolism
Root	–Fe–Zn	7.55E-11	61	178	Biosynthesis of amino acids
Root	–Fe–Zn	1.88E-09	32	70	Photosynthesis
Root	–Fe–Zn	8.44E-09	31	70	Glutathione metabolism
Root	–Fe–Zn	1.03E-08	24	46	Carbon fixation in photosynthetic organisms
Root	–Fe–Zn	8.53E-08	51	163	Spliceosome
Root	–Fe–Zn	2.91E-07	29	72	Phagosome
Root	–Fe–Zn	3.33E-07	36	102	Glycolysis/gluconeogenesis
Root	–Fe–Zn	3.33E-07	14	21	Photosynthesis
Root	–Fe–Zn	3.33E-07	29	73	Cysteine and methionine metabolism
Root	–Fe–Zn	4.94E-07	21	44	DNA replication
Root	–Fe–Zn	4.94E-07	54	188	Protein processing in endoplasmic reticulum

Root	−Fe−Zn	5.49E-07	22	48	Glyoxylate and dicarboxylate metabolism
Root	−Fe−Zn	1.06E-06	35	103	Amino sugar and nucleotide sugar metabolism
Root	−Fe−Zn	1.11E-06	40	126	RNA transport
Root	−Fe−Zn	2.12E-06	20	44	Alanine, aspartate and glutamate metabolism
Root	−Fe−Zn	6.81E-06	35	111	Starch and sucrose metabolism
Root	−Fe−Zn	2.77E-05	23	63	Pyruvate metabolism
Root	−Fe−Zn	5.96E-05	16	37	Fatty acid biosynthesis
Root	−Fe−Zn	1.17E-04	12	24	Arginine biosynthesis
Root	−Fe−Zn	1.17E-04	30	101	Ubiquitin mediated proteolysis
Root	−Fe−Zn	5.97E-04	17	48	Fructose and mannose metabolism
Root	−Fe−Zn	6.76E-04	15	40	Pentose phosphate pathway
Root	−Fe−Zn	9.61E-04	14	37	Arginine and proline metabolism
Root	−Fe−Zn	1.18E-03	15	42	2-Oxocarboxylic acid metabolism
Root	−Fe−Zn	1.19E-03	17	51	Citrate cycle (TCA cycle)
Root	−Fe−Zn	1.49E-03	20	66	Peroxisome
Shoot	−Zn	1.07E-17	106	1489	Metabolic pathways
Shoot	−Zn	2.35E-12	64	824	Biosynthesis of secondary metabolites
Shoot	−Zn	1.07E-09	16	70	Photosynthesis
Shoot	−Zn	6.08E-07	8	21	Photosynthesis
Shoot	−Zn	6.08E-07	13	70	Glutathione metabolism
Shoot	−Zn	1.23E-06	15	102	Glycolysis/gluconeogenesis
Shoot	−Zn	9.05E-06	11	63	Pyruvate metabolism
Shoot	−Zn	2.52E-04	8	46	Carbon fixation in photosynthetic organisms
Shoot	−Zn	5.37E-04	16	191	Carbon metabolism
Shoot	−Zn	1.10E-03	6	31	Ascorbate and aldarate metabolism
Shoot	−Zn	1.44E-03	6	33	Alpha-linolenic acid metabolism
Shoot	−Zn	3.66E-03	3	7	Benzoxazinoid biosynthesis
Shoot	−Zn	4.68E-03	10	111	Starch and sucrose metabolism

Shoot	–Zn	4.92E-03	3	8	Sesquiterpenoid and triterpenoid biosynthesis
Shoot	–Zn	8.87E-03	11	144	Phenylpropanoid biosynthesis
Shoot	–Zn	1.32E-02	5	37	Phenylalanine, tyrosine and tryptophan biosynthesis
Shoot	–Zn	1.47E-02	3	12	Linoleic acid metabolism
Shoot	–Zn	1.65E-02	5	40	Pentose phosphate pathway
Shoot	–Zn	1.67E-02	3	13	Taurine and hypotaurine metabolism
Shoot	–Zn	1.85E-02	12	188	Protein processing in endoplasmic reticulum
Shoot	–Zn	2.72E-02	5	48	Fructose and mannose metabolism
Shoot	–Zn	2.72E-02	5	48	Glyoxylate and dicarboxylate metabolism
Shoot	–Zn	2.72E-02	11	178	Biosynthesis of amino acids
Shoot	–Zn	2.72E-02	6	66	Peroxisome
Shoot	–Zn	2.79E-02	3	17	Stilbenoid, diarylheptanoid and gingerol biosynthesis
Shoot	–Zn	2.85E-02	7	90	Glycerophospholipid metabolism
Shoot	–Zn	2.85E-02	12	207	Plant hormone signal transduction
Shoot	–Zn	4.27E-02	4	37	Arginine and proline metabolism
Shoot	–Fe	1.57E-58	330	1489	Metabolic pathways
Shoot	–Fe	1.55E-31	182	824	Biosynthesis of secondary metabolites
Shoot	–Fe	7.66E-21	64	191	Carbon metabolism
Shoot	–Fe	4.12E-15	68	267	Ribosome
Shoot	–Fe	5.87E-15	53	178	Biosynthesis of amino acids
Shoot	–Fe	1.43E-14	54	188	Protein processing in endoplasmic reticulum
Shoot	–Fe	2.23E-13	30	70	Photosynthesis
Shoot	–Fe	3.94E-10	21	46	Carbon fixation in photosynthetic organisms
Shoot	–Fe	8.61E-09	30	101	Oxidative phosphorylation
Shoot	–Fe	8.61E-09	23	63	Pyruvate metabolism
Shoot	–Fe	9.94E-09	30	102	Glycolysis/gluconeogenesis
Shoot	–Fe	8.57E-08	38	163	Spliceosome
Shoot	–Fe	1.32E-07	19	51	Citrate cycle (TCA cycle)

Shoot	–Fe	1.60E-07	23	73	Cysteine and methionine metabolism
Shoot	–Fe	1.00E-06	28	111	Starch and sucrose metabolism
Shoot	–Fe	6.24E-06	14	37	Arginine and proline metabolism
Shoot	–Fe	6.24E-06	25	101	Ubiquitin mediated proteolysis
Shoot	–Fe	8.26E-06	25	103	Amino sugar and nucleotide sugar metabolism
Shoot	–Fe	3.70E-05	15	48	Glyoxylate and dicarboxylate metabolism
Shoot	–Fe	9.20E-05	18	70	Glutathione metabolism
Shoot	–Fe	1.32E-04	9	21	Photosynthesis
Shoot	–Fe	1.32E-04	18	72	Phagosome
Shoot	–Fe	6.16E-04	13	48	Fructose and mannose metabolism
Shoot	–Fe	6.16E-04	12	42	2-Oxocarboxylic acid metabolism
Shoot	–Fe	6.16E-04	13	48	Ribosome biogenesis in eukaryotes
Shoot	–Fe	1.24E-03	10	33	Valine, leucine and isoleucine degradation
Shoot	–Fe	1.54E-03	11	40	Aminoacyl-tRNA biosynthesis
Shoot	–Fe	1.54E-03	23	126	RNA transport
Shoot	–Fe	2.82E-03	11	43	Glycine, serine and threonine metabolism
Shoot	–Fe	3.03E-03	9	31	Ascorbate and aldarate metabolism
Shoot	–Fe–Zn	7.48E-80	169	267	Ribosome
Shoot	–Fe–Zn	3.43E-61	435	1489	Metabolic pathways
Shoot	–Fe–Zn	3.94E-32	238	824	Biosynthesis of secondary metabolites
Shoot	–Fe–Zn	1.06E-23	83	191	Carbon metabolism
Shoot	–Fe–Zn	5.30E-14	46	102	Glycolysis/gluconeogenesis
Shoot	–Fe–Zn	6.75E-14	61	163	Spliceosome
Shoot	–Fe–Zn	1.72E-12	35	70	Photosynthesis
Shoot	–Fe–Zn	7.07E-11	47	126	RNA transport
Shoot	–Fe–Zn	7.07E-11	61	188	Protein processing in endoplasmic reticulum
Shoot	–Fe–Zn	1.36E-10	27	51	Citrate cycle (TCA cycle)
Shoot	–Fe–Zn	1.48E-10	26	48	Glyoxylate and dicarboxylate metabolism

Shoot	–Fe–Zn	3.04E-10	25	46	Carbon fixation in photosynthetic organisms
Shoot	–Fe–Zn	1.25E-09	56	178	Biosynthesis of amino acids
Shoot	–Fe–Zn	1.41E-09	29	63	Pyruvate metabolism
Shoot	–Fe–Zn	2.76E-08	29	70	Glutathione metabolism
Shoot	–Fe–Zn	4.73E-08	36	101	Oxidative phosphorylation
Shoot	–Fe–Zn	1.29E-07	14	21	Photosynthesis
Shoot	–Fe–Zn	1.60E-07	21	44	DNA replication
Shoot	–Fe–Zn	3.11E-07	23	53	Proteasome
Shoot	–Fe–Zn	9.42E-07	21	48	Ribosome biogenesis in eukaryotes
Shoot	–Fe–Zn	4.38E-06	26	73	Cysteine and methionine metabolism
Shoot	–Fe–Zn	8.06E-06	32	103	Amino sugar and nucleotide sugar metabolism
Shoot	–Fe–Zn	1.98E-05	17	40	Pentose phosphate pathway
Shoot	–Fe–Zn	2.55E-05	15	33	Valine, leucine and isoleucine degradation
Shoot	–Fe–Zn	7.79E-05	18	48	Fructose and mannose metabolism
Shoot	–Fe–Zn	8.48E-05	16	40	Aminoacyl-tRNA biosynthesis
Shoot	–Fe–Zn	1.12E-04	23	72	Phagosome
Shoot	–Fe–Zn	2.80E-04	28	101	Ubiquitin mediated proteolysis
Shoot	–Fe–Zn	3.01E-04	16	44	Alanine, aspartate and glutamate metabolism
Shoot	–Fe–Zn	3.51E-04	12	28	Propanoate metabolism

Table S3. The predicted miRNA–gene and TF–gene interactions used to construct the gene regulatory network for the transporter and mugineic acid pathway genes.

S. No	Target	Target Description	Type	Source	Source Description
1	GRMZM2G014914	Plasma membrane intrinsic protein 2	miRNA–DEG	zma-miR397b-3p	zma-miR397b-3p
2	GRMZM2G014914	Plasma membrane intrinsic protein 2	miRNA–DEG	zma-miR167d-3p	zma-miR167d-3p
3	GRMZM2G014914	Plasma membrane intrinsic protein 2	miRNA–DEG	zma-miR171g-3p	zma-miR171g-3p
4	GRMZM2G014914	Plasma membrane intrinsic protein 2	miRNA–DEG	zma-miR167c-3p	zma-miR167c-3p
5	GRMZM2G014914	Plasma membrane intrinsic protein 2	TF–DEG	AC187157.4_FG005	HD-ZIP
6	GRMZM2G014914	Plasma membrane intrinsic protein 2	TF–DEG	GRMZM2G069274	WOX
7	GRMZM2G014914	Plasma membrane intrinsic protein 2	TF–DEG	GRMZM2G135381	GATA
8	GRMZM2G015295	Adenosylhomocysteinase	miRNA–DEG	zma-miR168a-3p	zma-miR168a-3p
9	GRMZM2G015295	Adenosylhomocysteinase	miRNA–DEG	zma-miR159f-5p	zma-miR159f-5p
10	GRMZM2G015295	Adenosylhomocysteinase	miRNA–DEG	zma-miR827-3p	zma-miR827-3p
11	GRMZM2G015295	Adenosylhomocysteinase	miRNA–DEG	zma-miR168b-3p	zma-miR168b-3p
12	GRMZM2G015295	Adenosylhomocysteinase	TF–DEG	AC198403.3_FG001	HSF
13	GRMZM2G015295	Adenosylhomocysteinase	TF–DEG	AC233943.1_FG002	LBD
14	GRMZM2G015295	Adenosylhomocysteinase	TF–DEG	GRMZM2G003514	MIKC_MADS
15	GRMZM2G015295	Adenosylhomocysteinase	TF–DEG	GRMZM2G010100	ERF
16	GRMZM2G015295	Adenosylhomocysteinase	TF–DEG	GRMZM2G018398	ERF
17	GRMZM2G015295	Adenosylhomocysteinase	TF–DEG	GRMZM2G021790	ERF
18	GRMZM2G015295	Adenosylhomocysteinase	TF–DEG	GRMZM2G023708	ERF
19	GRMZM2G015295	Adenosylhomocysteinase	TF–DEG	GRMZM2G040924	MYB
20	GRMZM2G015295	Adenosylhomocysteinase	TF–DEG	GRMZM2G045748	MYB
21	GRMZM2G015295	Adenosylhomocysteinase	TF–DEG	GRMZM2G057386	ERF
22	GRMZM2G015295	Adenosylhomocysteinase	TF–DEG	GRMZM2G060517	ERF
23	GRMZM2G015295	Adenosylhomocysteinase	TF–DEG	GRMZM2G079653	ERF
24	GRMZM2G015295	Adenosylhomocysteinase	TF–DEG	GRMZM2G111415	ERF
25	GRMZM2G015295	Adenosylhomocysteinase	TF–DEG	GRMZM2G127490	MYB

26	GRMZM2G015295	Adenosylhomocysteinase	TF–DEG	GRMZM2G139073	MIKC_MADS
27	GRMZM2G015295	Adenosylhomocysteinase	TF–DEG	GRMZM2G142179	ERF
28	GRMZM2G015295	Adenosylhomocysteinase	TF–DEG	GRMZM2G164735	BBR-BPC
29	GRMZM2G015295	Adenosylhomocysteinase	TF–DEG	GRMZM2G307119	ERF
30	GRMZM2G015295	Adenosylhomocysteinase	TF–DEG	GRMZM2G310368	ERF
31	GRMZM2G015295	Adenosylhomocysteinase	TF–DEG	GRMZM2G359952	MIKC_MADS
32	GRMZM2G015295	Adenosylhomocysteinase	TF–DEG	GRMZM2G376255	ERF
33	GRMZM2G015295	Adenosylhomocysteinase	TF–DEG	GRMZM2G381441	ERF
34	GRMZM2G015401	Mitochondrial phosphate transporter	miRNA–DEG	zma-miR164d-3p	zma-miR164d-3p
35	GRMZM2G015401	Mitochondrial phosphate transporter	miRNA–DEG	zma-miR159f-5p	zma-miR159f-5p
36	GRMZM2G015401	Mitochondrial phosphate transporter	TF–DEG	AC233943.1_FG002	LBD
37	GRMZM2G015401	Mitochondrial phosphate transporter	TF–DEG	GRMZM2G003944	TCP
38	GRMZM2G015401	Mitochondrial phosphate transporter	TF–DEG	GRMZM2G018398	ERF
39	GRMZM2G015401	Mitochondrial phosphate transporter	TF–DEG	GRMZM2G092214	TCP
40	GRMZM2G015401	Mitochondrial phosphate transporter	TF–DEG	GRMZM2G111415	ERF
41	GRMZM2G015401	Mitochondrial phosphate transporter	TF–DEG	GRMZM2G142751	TCP
42	GRMZM2G015401	Mitochondrial phosphate transporter	TF–DEG	GRMZM2G310368	ERF
43	GRMZM2G015401	Mitochondrial phosphate transporter	TF–DEG	GRMZM2G445944	TCP
44	GRMZM2G015955	Zinc transporter 4	miRNA–DEG	zma-miR395c-3p	zma-miR395c-3p
45	GRMZM2G015955	Zinc transporter 4	miRNA–DEG	zma-miR395l-3p	zma-miR395l-3p
46	GRMZM2G015955	Zinc transporter 4	miRNA–DEG	zma-miR395m-3p	zma-miR395m-3p
47	GRMZM2G015955	Zinc transporter 4	miRNA–DEG	zma-miR395o-3p	zma-miR395o-3p
48	GRMZM2G015955	Zinc transporter 4	miRNA–DEG	zma-miR395a-3p	zma-miR395a-3p
49	GRMZM2G015955	Zinc transporter 4	miRNA–DEG	zma-miR395b-3p	zma-miR395b-3p
50	GRMZM2G015955	Zinc transporter 4	miRNA–DEG	zma-miR395d-3p	zma-miR395d-3p
51	GRMZM2G015955	Zinc transporter 4	miRNA–DEG	zma-miR395e-3p	zma-miR395e-3p
52	GRMZM2G015955	Zinc transporter 4	miRNA–DEG	zma-miR395f-3p	zma-miR395f-3p
53	GRMZM2G015955	Zinc transporter 4	miRNA–DEG	zma-miR395g-3p	zma-miR395g-3p

54	GRMZM2G015955	Zinc transporter 4	miRNA–DEG	zma-miR395h-3p	zma-miR395h-3p
55	GRMZM2G015955	Zinc transporter 4	miRNA–DEG	zma-miR395i-3p	zma-miR395i-3p
56	GRMZM2G015955	Zinc transporter 4	miRNA–DEG	zma-miR395j-3p	zma-miR395j-3p
57	GRMZM2G015955	Zinc transporter 4	miRNA–DEG	zma-miR395n-3p	zma-miR395n-3p
58	GRMZM2G015955	Zinc transporter 4	miRNA–DEG	zma-miR395p-3p	zma-miR395p-3p
59	GRMZM2G015955	Zinc transporter 4	miRNA–DEG	zma-miR2275a-5p	zma-miR2275a-5p
60	GRMZM2G015955	Zinc transporter 4	TF–DEG	AC205574.3_FG006	TCP
61	GRMZM2G015955	Zinc transporter 4	TF–DEG	GRMZM2G003944	TCP
62	GRMZM2G015955	Zinc transporter 4	TF–DEG	GRMZM2G004531	NAC
63	GRMZM2G015955	Zinc transporter 4	TF–DEG	GRMZM2G034638	TCP
64	GRMZM2G015955	Zinc transporter 4	TF–DEG	GRMZM2G089501	bHLH
65	GRMZM2G015955	Zinc transporter 4	TF–DEG	GRMZM2G092214	TCP
66	GRMZM2G015955	Zinc transporter 4	TF–DEG	GRMZM2G107031	TCP
67	GRMZM2G015955	Zinc transporter 4	TF–DEG	GRMZM2G111415	ERF
68	GRMZM2G015955	Zinc transporter 4	TF–DEG	GRMZM2G142751	TCP
69	GRMZM2G015955	Zinc transporter 4	TF–DEG	GRMZM2G164735	BBR-BPC
70	GRMZM2G015955	Zinc transporter 4	TF–DEG	GRMZM2G165042	bHLH
71	GRMZM2G015955	Zinc transporter 4	TF–DEG	GRMZM2G171365	MIKC_MADS
72	GRMZM2G015955	Zinc transporter 4	TF–DEG	GRMZM2G178603	TCP
73	GRMZM2G015955	Zinc transporter 4	TF–DEG	GRMZM2G376255	ERF
74	GRMZM2G015955	Zinc transporter 4	TF–DEG	GRMZM2G445944	TCP
75	GRMZM2G027098	Tonoplast intrinsic protein 2	miRNA–DEG	zma-miR396e-3p	zma-miR396e-3p
76	GRMZM2G027098	Tonoplast intrinsic protein 2	TF–DEG	GRMZM2G050939	C2H2
77	GRMZM2G027098	Tonoplast intrinsic protein 2	TF–DEG	GRMZM2G140694	Dof
78	GRMZM2G027098	Tonoplast intrinsic protein 2	TF–DEG	GRMZM2G144744	GRAS
79	GRMZM2G027098	Tonoplast intrinsic protein 2	TF–DEG	GRMZM2G171365	MIKC_MADS
80	GRMZM2G036908	Cation transmembrane transporter	miRNA–DEG	zma-miR167f-3p	zma-miR167f-3p
81	GRMZM2G036908	Cation transmembrane transporter	miRNA–DEG	zma-miR390a-5p	zma-miR390a-5p

82	GRMZM2G036908	Cation transmembrane transporter	miRNA–DEG	zma-miR390b-5p	zma-miR390b-5p
83	GRMZM2G036908	Cation transmembrane transporter	miRNA–DEG	zma-miR396g-5p	zma-miR396g-5p
84	GRMZM2G036908	Cation transmembrane transporter	miRNA–DEG	zma-miR396h	zma-miR396h
85	GRMZM2G036908	Cation transmembrane transporter	miRNA–DEG	zma-miR2275d-5p	zma-miR2275d-5p
86	GRMZM2G036908	Cation transmembrane transporter	miRNA–DEG	zma-miR167e-3p	zma-miR167e-3p
87	GRMZM2G036908	Cation transmembrane transporter	TF–DEG	GRMZM2G110153	MIKC_MADS
88	GRMZM2G036908	Cation transmembrane transporter	TF–DEG	GRMZM2G171365	MIKC_MADS
89	GRMZM2G036908	Cation transmembrane transporter	TF–DEG	GRMZM2G359952	MIKC_MADS
90	GRMZM2G050108	Nicotianamine synthase 3	miRNA–DEG	zma-miR160f-3p	zma-miR160f-3p
91	GRMZM2G050108	Nicotianamine synthase 3	TF–DEG	GRMZM2G101499	SBP
92	GRMZM2G054123	S-adenosylmethionine synthetase 1	TF–DEG	AC205574.3_FG006	TCP
93	GRMZM2G054123	S-adenosylmethionine synthetase 1	TF–DEG	GRMZM2G003944	TCP
94	GRMZM2G054123	S-adenosylmethionine synthetase 1	TF–DEG	GRMZM2G011357	C2H2
95	GRMZM2G054123	S-adenosylmethionine synthetase 1	TF–DEG	GRMZM2G017470	Dof
96	GRMZM2G054123	S-adenosylmethionine synthetase 1	TF–DEG	GRMZM2G018398	ERF
97	GRMZM2G054123	S-adenosylmethionine synthetase 1	TF–DEG	GRMZM2G023708	ERF
98	GRMZM2G054123	S-adenosylmethionine synthetase 1	TF–DEG	GRMZM2G034638	TCP
99	GRMZM2G054123	S-adenosylmethionine synthetase 1	TF–DEG	GRMZM2G040924	MYB
100	GRMZM2G054123	S-adenosylmethionine synthetase 1	TF–DEG	GRMZM2G046885	MIKC_MADS
101	GRMZM2G054123	S-adenosylmethionine synthetase 1	TF–DEG	GRMZM2G057386	ERF
102	GRMZM2G054123	S-adenosylmethionine synthetase 1	TF–DEG	GRMZM2G093725	Dof
103	GRMZM2G054123	S-adenosylmethionine synthetase 1	TF–DEG	GRMZM2G095904	MYB
104	GRMZM2G054123	S-adenosylmethionine synthetase 1	TF–DEG	GRMZM2G127490	MYB
105	GRMZM2G054123	S-adenosylmethionine synthetase 1	TF–DEG	GRMZM2G140694	Dof
106	GRMZM2G054123	S-adenosylmethionine synthetase 1	TF–DEG	GRMZM2G142179	ERF
107	GRMZM2G054123	S-adenosylmethionine synthetase 1	TF–DEG	GRMZM2G165042	bHLH
108	GRMZM2G054123	S-adenosylmethionine synthetase 1	TF–DEG	GRMZM2G171365	MIKC_MADS
109	GRMZM2G054123	S-adenosylmethionine synthetase 1	TF–DEG	GRMZM2G172327	MYB

110	GRMZM2G054123	S-adenosylmethionine synthetase 1	TF–DEG	GRMZM2G310368	ERF
111	GRMZM2G054123	S-adenosylmethionine synthetase 1	TF–DEG	GRMZM2G394941	Dof
112	GRMZM2G054123	S-adenosylmethionine synthetase 1	TF–DEG	GRMZM5G833253	MYB
113	GRMZM2G056908	Tonoplast intrinsic protein 2	miRNA–DEG	zma-miR398b-5p	zma-miR398b-5p
114	GRMZM2G064023	Citrate Synthase 2	miRNA–DEG	zma-miR171d-5p	zma-miR171d-5p
115	GRMZM2G064023	Citrate Synthase 2	miRNA–DEG	zma-miR171e-5p	zma-miR171e-5p
116	GRMZM2G064023	Citrate Synthase 2	miRNA–DEG	zma-miR395d-5p	zma-miR395d-5p
117	GRMZM2G064023	Citrate Synthase 2	miRNA–DEG	zma-miR395g-5p	zma-miR395g-5p
118	GRMZM2G064023	Citrate Synthase 2	TF–DEG	GRMZM2G017087	TALE
119	GRMZM2G064023	Citrate Synthase 2	TF–DEG	GRMZM2G171365	MIKC_MADS
120	GRMZM2G064382	ZRT-IRT-like protein 5	miRNA–DEG	zma-miR408b-5p	zma-miR408b-5p
121	GRMZM2G064382	ZRT-IRT-like protein 5	miRNA–DEG	zma-miR482-3p	zma-miR482-3p
122	GRMZM2G064382	ZRT-IRT-like protein 5	TF–DEG	GRMZM2G018398	ERF
123	GRMZM2G064382	ZRT-IRT-like protein 5	TF–DEG	GRMZM2G021790	ERF
124	GRMZM2G064382	ZRT-IRT-like protein 5	TF–DEG	GRMZM2G030710	ARF
125	GRMZM2G064382	ZRT-IRT-like protein 5	TF–DEG	GRMZM2G060517	ERF
126	GRMZM2G064382	ZRT-IRT-like protein 5	TF–DEG	GRMZM2G066158	ERF
127	GRMZM2G064382	ZRT-IRT-like protein 5	TF–DEG	GRMZM2G079653	ERF
128	GRMZM2G064382	ZRT-IRT-like protein 5	TF–DEG	GRMZM2G092214	TCP
129	GRMZM2G064382	ZRT-IRT-like protein 5	TF–DEG	GRMZM2G107031	TCP
130	GRMZM2G064382	ZRT-IRT-like protein 5	TF–DEG	GRMZM2G142179	ERF
131	GRMZM2G064382	ZRT-IRT-like protein 5	TF–DEG	GRMZM2G173633	MYB
132	GRMZM2G064382	ZRT-IRT-like protein 5	TF–DEG	GRMZM2G307119	ERF
133	GRMZM2G064382	ZRT-IRT-like protein 5	TF–DEG	GRMZM2G310368	ERF
134	GRMZM2G067546	Vacuolar sorting receptor homolog 1	miRNA–DEG	zma-miR395e-5p	zma-miR395e-5p
135	GRMZM2G067546	Vacuolar sorting receptor homolog 1	miRNA–DEG	zma-miR395h-5p	zma-miR395h-5p
136	GRMZM2G067546	Vacuolar sorting receptor homolog 1	miRNA–DEG	zma-miR395j-5p	zma-miR395j-5p
137	GRMZM2G067546	Vacuolar sorting receptor homolog 1	miRNA–DEG	zma-miR395m-5p	zma-miR395m-5p

138	GRMZM2G067546	Vacuolar sorting receptor homolog 1	miRNA–DEG	zma-miR395p-5p	zma-miR395p-5p
139	GRMZM2G067546	Vacuolar sorting receptor homolog 1	TF–DEG	GRMZM2G010100	ERF
140	GRMZM2G067546	Vacuolar sorting receptor homolog 1	TF–DEG	GRMZM2G017087	TALE
141	GRMZM2G067546	Vacuolar sorting receptor homolog 1	TF–DEG	GRMZM2G035701	B3
142	GRMZM2G067546	Vacuolar sorting receptor homolog 1	TF–DEG	GRMZM2G341747	CAMTA
143	GRMZM2G069198	NRAMP transporter1	miRNA–DEG	zma-miR159g-3p	zma-miR159g-3p
144	GRMZM2G069198	NRAMP transporter1	miRNA–DEG	zma-miR159h-3p	zma-miR159h-3p
145	GRMZM2G069198	NRAMP transporter1	miRNA–DEG	zma-miR159i-3p	zma-miR159i-3p
146	GRMZM2G069198	NRAMP transporter1	miRNA–DEG	zma-miR171j-5p	zma-miR171j-5p
147	GRMZM2G069198	NRAMP transporter1	TF–DEG	GRMZM2G017470	Dof
148	GRMZM2G069198	NRAMP transporter1	TF–DEG	GRMZM2G093725	Dof
149	GRMZM2G069198	NRAMP transporter1	TF–DEG	GRMZM2G112548	NAC
150	GRMZM2G069198	NRAMP transporter1	TF–DEG	GRMZM2G140694	Dof
151	GRMZM2G069198	NRAMP transporter1	TF–DEG	GRMZM2G164735	BBR-BPC
152	GRMZM2G069198	NRAMP transporter1	TF–DEG	GRMZM2G171073	C2H2
153	GRMZM2G069198	NRAMP transporter1	TF–DEG	GRMZM2G171365	MIKC_MADS
154	GRMZM2G069198	NRAMP transporter1	TF–DEG	GRMZM2G394941	Dof
155	GRMZM2G069198	NRAMP transporter1	TF–DEG	GRMZM5G828179	C2H2
156	GRMZM2G070360	V-type proton ATPase subunit E3	miRNA–DEG	zma-miR398a-5p	zma-miR398a-5p
157	GRMZM2G070360	V-type proton ATPase subunit E3	miRNA–DEG	zma-miR171b-3p	zma-miR171b-3p
158	GRMZM2G070360	V-type proton ATPase subunit E3	miRNA–DEG	zma-miR171f-3p	zma-miR171f-3p
159	GRMZM2G070360	V-type proton ATPase subunit E3	miRNA–DEG	zma-miR171g-3p	zma-miR171g-3p
160	GRMZM2G070360	V-type proton ATPase subunit E3	miRNA–DEG	zma-miR171h-3p	zma-miR171h-3p
161	GRMZM2G070360	V-type proton ATPase subunit E3	miRNA–DEG	zma-miR171k-3p	zma-miR171k-3p
162	GRMZM2G070360	V-type proton ATPase subunit E3	TF–DEG	AC216247.3_FG001	HSF
163	GRMZM2G070360	V-type proton ATPase subunit E3	TF–DEG	AC233960.1_FG003	G2-like
164	GRMZM2G070360	V-type proton ATPase subunit E3	TF–DEG	GRMZM2G009060	G2-like
165	GRMZM2G070360	V-type proton ATPase subunit E3	TF–DEG	GRMZM2G017470	Dof

166	GRMZM2G070360	V-type proton ATPase subunit E3	TF–DEG	GRMZM2G075956	C2H2
167	GRMZM2G070360	V-type proton ATPase subunit E3	TF–DEG	GRMZM2G093725	Dof
168	GRMZM2G070360	V-type proton ATPase subunit E3	TF–DEG	GRMZM2G105348	HSF
169	GRMZM2G070360	V-type proton ATPase subunit E3	TF–DEG	GRMZM2G115456	HSF
170	GRMZM2G070360	V-type proton ATPase subunit E3	TF–DEG	GRMZM2G125969	HSF
171	GRMZM2G070360	V-type proton ATPase subunit E3	TF–DEG	GRMZM2G159119	G2-like
172	GRMZM2G070360	V-type proton ATPase subunit E3	TF–DEG	GRMZM2G164909	HSF
173	GRMZM2G070360	V-type proton ATPase subunit E3	TF–DEG	GRMZM2G173943	G2-like
174	GRMZM2G070360	V-type proton ATPase subunit E3	TF–DEG	GRMZM2G348238	G2-like
175	GRMZM2G070360	V-type proton ATPase subunit E3	TF–DEG	GRMZM2G394941	Dof
176	GRMZM2G070360	V-type proton ATPase subunit E3	TF–DEG	GRMZM2G477238	G2-like
177	GRMZM2G070605	S-adenosylmethionine decarboxylase proenzyme	miRNA–DEG	zma-miR169m-5p	zma-miR169m-5p
178	GRMZM2G070605	S-adenosylmethionine decarboxylase proenzyme	miRNA–DEG	zma-miR169n-5p	zma-miR169n-5p
179	GRMZM2G070605	S-adenosylmethionine decarboxylase proenzyme	miRNA–DEG	zma-miR169q-5p	zma-miR169q-5p
180	GRMZM2G070605	S-adenosylmethionine decarboxylase proenzyme	miRNA–DEG	zma-miR171i-5p	zma-miR171i-5p
181	GRMZM2G070605	S-adenosylmethionine decarboxylase proenzyme	TF–DEG	GRMZM2G010100	ERF
182	GRMZM2G070605	S-adenosylmethionine decarboxylase proenzyme	TF–DEG	GRMZM2G017087	TALE
183	GRMZM2G070605	S-adenosylmethionine decarboxylase proenzyme	TF–DEG	GRMZM2G017470	Dof
184	GRMZM2G070605	S-adenosylmethionine decarboxylase proenzyme	TF–DEG	GRMZM2G079653	ERF
185	GRMZM2G070605	S-adenosylmethionine decarboxylase proenzyme	TF–DEG	GRMZM2G093595	ERF
186	GRMZM2G070605	S-adenosylmethionine decarboxylase proenzyme	TF–DEG	GRMZM2G093725	Dof
187	GRMZM2G070605	S-adenosylmethionine decarboxylase proenzyme	TF–DEG	GRMZM2G144744	GRAS
188	GRMZM2G070605	S-adenosylmethionine decarboxylase proenzyme	TF–DEG	GRMZM2G307119	ERF
189	GRMZM2G070605	S-adenosylmethionine decarboxylase proenzyme	TF–DEG	GRMZM2G307588	SBP
190	GRMZM2G070605	S-adenosylmethionine decarboxylase proenzyme	TF–DEG	GRMZM2G394941	Dof
191	GRMZM2G070605	S-adenosylmethionine decarboxylase proenzyme	TF–DEG	GRMZM5G833032	MYB_related
192	GRMZM2G092125	Plasma membrane intrinsic protein 2	miRNA–DEG	zma-miR167d-3p	zma-miR167d-3p
193	GRMZM2G092125	Plasma membrane intrinsic protein 2	miRNA–DEG	zma-miR164d-3p	zma-miR164d-3p

194	GRMZM2G092125	Plasma membrane intrinsic protein 2	miRNA–DEG	zma-miR167c-3p	zma-miR167c-3p
195	GRMZM2G092125	Plasma membrane intrinsic protein 2	miRNA–DEG	zma-miR482-5p	zma-miR482-5p
196	GRMZM2G092125	Plasma membrane intrinsic protein 2	TF–DEG	GRMZM2G010920	G2-like
197	GRMZM2G092125	Plasma membrane intrinsic protein 2	TF–DEG	GRMZM2G017087	TALE
198	GRMZM2G092125	Plasma membrane intrinsic protein 2	TF–DEG	GRMZM2G105348	HSF
199	GRMZM2G092125	Plasma membrane intrinsic protein 2	TF–DEG	GRMZM2G110153	MIKC_MADS
200	GRMZM2G092125	Plasma membrane intrinsic protein 2	TF–DEG	GRMZM2G118690	BBR-BPC
201	GRMZM2G092125	Plasma membrane intrinsic protein 2	TF–DEG	GRMZM2G140694	Dof
202	GRMZM2G092125	Plasma membrane intrinsic protein 2	TF–DEG	GRMZM2G143723	C2H2
203	GRMZM2G092125	Plasma membrane intrinsic protein 2	TF–DEG	GRMZM2G144744	GRAS
204	GRMZM2G092125	Plasma membrane intrinsic protein 2	TF–DEG	GRMZM2G164735	BBR-BPC
205	GRMZM2G092125	Plasma membrane intrinsic protein 2	TF–DEG	GRMZM2G171073	C2H2
206	GRMZM2G092125	Plasma membrane intrinsic protein 2	TF–DEG	GRMZM2G171365	MIKC_MADS
207	GRMZM2G092125	Plasma membrane intrinsic protein 2	TF–DEG	GRMZM2G179677	C2H2
208	GRMZM2G092125	Plasma membrane intrinsic protein 2	TF–DEG	GRMZM2G320287	C2H2
209	GRMZM2G092125	Plasma membrane intrinsic protein 2	TF–DEG	GRMZM5G828179	C2H2
210	GRMZM2G094497	Vacuolar ATP synthase subunit B	miRNA–DEG	zma-miR395m-5p	zma-miR395m-5p
211	GRMZM2G094497	Vacuolar ATP synthase subunit B	TF–DEG	AC198403.3_FG001	HSF
212	GRMZM2G094497	Vacuolar ATP synthase subunit B	TF–DEG	GRMZM2G009060	G2-like
213	GRMZM2G094497	Vacuolar ATP synthase subunit B	TF–DEG	GRMZM2G026643	HD-ZIP
214	GRMZM2G094497	Vacuolar ATP synthase subunit B	TF–DEG	GRMZM2G126646	HD-ZIP
215	GRMZM2G094497	Vacuolar ATP synthase subunit B	TF–DEG	GRMZM2G139963	HD-ZIP
216	GRMZM2G094497	Vacuolar ATP synthase subunit B	TF–DEG	GRMZM2G164735	BBR-BPC
217	GRMZM2G094497	Vacuolar ATP synthase subunit B	TF–DEG	GRMZM2G171365	MIKC_MADS
218	GRMZM2G094497	Vacuolar ATP synthase subunit B	TF–DEG	GRMZM2G477238	G2-like
219	GRMZM2G099340	Metallothionein-like protein type 2	miRNA–DEG	zma-miR160c-3p	zma-miR160c-3p
220	GRMZM2G099340	Metallothionein-like protein type 2	miRNA–DEG	zma-miR160d-3p	zma-miR160d-3p
221	GRMZM2G099340	Metallothionein-like protein type 2	miRNA–DEG	zma-miR156a-5p	zma-miR156a-5p

222	GRMZM2G099340	Metallothionein-like protein type 2	miRNA–DEG	zma-miR156b-5p	zma-miR156b-5p
223	GRMZM2G099340	Metallothionein-like protein type 2	miRNA–DEG	zma-miR156c	zma-miR156c
224	GRMZM2G099340	Metallothionein-like protein type 2	miRNA–DEG	zma-miR156d-5p	zma-miR156d-5p
225	GRMZM2G099340	Metallothionein-like protein type 2	miRNA–DEG	zma-miR156e-5p	zma-miR156e-5p
226	GRMZM2G099340	Metallothionein-like protein type 2	miRNA–DEG	zma-miR156f-5p	zma-miR156f-5p
227	GRMZM2G099340	Metallothionein-like protein type 2	miRNA–DEG	zma-miR156g-5p	zma-miR156g-5p
228	GRMZM2G099340	Metallothionein-like protein type 2	miRNA–DEG	zma-miR156h-5p	zma-miR156h-5p
229	GRMZM2G099340	Metallothionein-like protein type 2	miRNA–DEG	zma-miR156i-5p	zma-miR156i-5p
230	GRMZM2G099340	Metallothionein-like protein type 2	miRNA–DEG	zma-miR156l-5p	zma-miR156l-5p
231	GRMZM2G099340	Metallothionein-like protein type 2	TF–DEG	AC233943.1_FG002	LBD
232	GRMZM2G099340	Metallothionein-like protein type 2	TF–DEG	GRMZM2G010100	ERF
233	GRMZM2G099340	Metallothionein-like protein type 2	TF–DEG	GRMZM2G018398	ERF
234	GRMZM2G099340	Metallothionein-like protein type 2	TF–DEG	GRMZM2G021790	ERF
235	GRMZM2G099340	Metallothionein-like protein type 2	TF–DEG	GRMZM2G023708	ERF
236	GRMZM2G099340	Metallothionein-like protein type 2	TF–DEG	GRMZM2G057386	ERF
237	GRMZM2G099340	Metallothionein-like protein type 2	TF–DEG	GRMZM2G066158	ERF
238	GRMZM2G099340	Metallothionein-like protein type 2	TF–DEG	GRMZM2G079653	ERF
239	GRMZM2G099340	Metallothionein-like protein type 2	TF–DEG	GRMZM2G111415	ERF
240	GRMZM2G099340	Metallothionein-like protein type 2	TF–DEG	GRMZM2G118250	LBD
241	GRMZM2G099340	Metallothionein-like protein type 2	TF–DEG	GRMZM2G138396	ERF
242	GRMZM2G099340	Metallothionein-like protein type 2	TF–DEG	GRMZM2G140694	Dof
243	GRMZM2G099340	Metallothionein-like protein type 2	TF–DEG	GRMZM2G142179	ERF
244	GRMZM2G099340	Metallothionein-like protein type 2	TF–DEG	GRMZM2G144744	GRAS
245	GRMZM2G099340	Metallothionein-like protein type 2	TF–DEG	GRMZM2G171365	MIKC_MADS
246	GRMZM2G099340	Metallothionein-like protein type 2	TF–DEG	GRMZM2G307119	ERF
247	GRMZM2G099340	Metallothionein-like protein type 2	TF–DEG	GRMZM2G310368	ERF
248	GRMZM2G099340	Metallothionein-like protein type 2	TF–DEG	GRMZM2G376255	ERF
249	GRMZM2G099340	Metallothionein-like protein type 2	TF–DEG	GRMZM2G381441	ERF

250	GRMZM2G099628	Probable methionine-tRNA ligase	miRNA–DEG	zma-miR160a-5p	zma-miR160a-5p
251	GRMZM2G099628	Probable methionine-tRNA ligase	miRNA–DEG	zma-miR160b-5p	zma-miR160b-5p
252	GRMZM2G099628	Probable methionine-tRNA ligase	miRNA–DEG	zma-miR160c-5p	zma-miR160c-5p
253	GRMZM2G099628	Probable methionine-tRNA ligase	miRNA–DEG	zma-miR160d-5p	zma-miR160d-5p
254	GRMZM2G099628	Probable methionine-tRNA ligase	miRNA–DEG	zma-miR160e	zma-miR160e
255	GRMZM2G099628	Probable methionine-tRNA ligase	miRNA–DEG	zma-miR160f-5p	zma-miR160f-5p
256	GRMZM2G099628	Probable methionine-tRNA ligase	miRNA–DEG	zma-miR160g-5p	zma-miR160g-5p
257	GRMZM2G099628	Probable methionine-tRNA ligase	miRNA–DEG	zma-miR164c-3p	zma-miR164c-3p
258	GRMZM2G099628	Probable methionine-tRNA ligase	miRNA–DEG	zma-miR164f-3p	zma-miR164f-3p
259	GRMZM2G099628	Probable methionine-tRNA ligase	miRNA–DEG	zma-miR164h-3p	zma-miR164h-3p
260	GRMZM2G099628	Probable methionine-tRNA ligase	TF–DEG	GRMZM2G010100	ERF
261	GRMZM2G099628	Probable methionine-tRNA ligase	TF–DEG	GRMZM2G018398	ERF
262	GRMZM2G099628	Probable methionine-tRNA ligase	TF–DEG	GRMZM2G021790	ERF
263	GRMZM2G099628	Probable methionine-tRNA ligase	TF–DEG	GRMZM2G023708	ERF
264	GRMZM2G099628	Probable methionine-tRNA ligase	TF–DEG	GRMZM2G057386	ERF
265	GRMZM2G099628	Probable methionine-tRNA ligase	TF–DEG	GRMZM2G060517	ERF
266	GRMZM2G099628	Probable methionine-tRNA ligase	TF–DEG	GRMZM2G066158	ERF
267	GRMZM2G099628	Probable methionine-tRNA ligase	TF–DEG	GRMZM2G079653	ERF
268	GRMZM2G099628	Probable methionine-tRNA ligase	TF–DEG	GRMZM2G092214	TCP
269	GRMZM2G099628	Probable methionine-tRNA ligase	TF–DEG	GRMZM2G100593	NAC
270	GRMZM2G099628	Probable methionine-tRNA ligase	TF–DEG	GRMZM2G107031	TCP
271	GRMZM2G099628	Probable methionine-tRNA ligase	TF–DEG	GRMZM2G111415	ERF
272	GRMZM2G099628	Probable methionine-tRNA ligase	TF–DEG	GRMZM2G118250	LBD
273	GRMZM2G099628	Probable methionine-tRNA ligase	TF–DEG	GRMZM2G138396	ERF
274	GRMZM2G099628	Probable methionine-tRNA ligase	TF–DEG	GRMZM2G138967	GATA
275	GRMZM2G099628	Probable methionine-tRNA ligase	TF–DEG	GRMZM2G142179	ERF
276	GRMZM2G099628	Probable methionine-tRNA ligase	TF–DEG	GRMZM2G307119	ERF
277	GRMZM2G099628	Probable methionine-tRNA ligase	TF–DEG	GRMZM2G310368	ERF

278	GRMZM2G099628	Probable methionine-tRNA ligase	TF–DEG	GRMZM2G376255	ERF
279	GRMZM2G099628	Probable methionine-tRNA ligase	TF–DEG	GRMZM2G381441	ERF
280	GRMZM2G099628	Probable methionine-tRNA ligase	TF–DEG	GRMZM2G445944	TCP
281	GRMZM2G104418	Proton-transporting V-type ATPase, V0 domain	miRNA–DEG	zma-miR2275d-3p	zma-miR2275d-3p
282	GRMZM2G104418	Proton-transporting V-type ATPase, V0 domain	miRNA–DEG	zma-miR390a-5p	zma-miR390a-5p
283	GRMZM2G104418	Proton-transporting V-type ATPase, V0 domain	miRNA–DEG	zma-miR390b-5p	zma-miR390b-5p
284	GRMZM2G104418	Proton-transporting V-type ATPase, V0 domain	TF–DEG	GRMZM2G035701	B3
285	GRMZM2G104418	Proton-transporting V-type ATPase, V0 domain	TF–DEG	GRMZM2G050305	MYB
286	GRMZM2G104418	Proton-transporting V-type ATPase, V0 domain	TF–DEG	GRMZM2G095904	MYB
287	GRMZM2G104418	Proton-transporting V-type ATPase, V0 domain	TF–DEG	GRMZM2G140694	Dof
288	GRMZM2G104418	Proton-transporting V-type ATPase, V0 domain	TF–DEG	GRMZM2G153754	CPP
289	GRMZM2G122437	Metal ion transporter	miRNA–DEG	zma-miR528a-3p	zma-miR528a-3p
290	GRMZM2G122437	Metal ion transporter	miRNA–DEG	zma-miR528b-3p	zma-miR528b-3p
291	GRMZM2G122437	Metal ion transporter	TF–DEG	AC233943.1_FG002	LBD
292	GRMZM2G122437	Metal ion transporter	TF–DEG	GRMZM2G017087	TALE
293	GRMZM2G122437	Metal ion transporter	TF–DEG	GRMZM2G050939	C2H2
294	GRMZM2G122437	Metal ion transporter	TF–DEG	GRMZM2G060517	ERF
295	GRMZM2G122437	Metal ion transporter	TF–DEG	GRMZM2G118690	BBR-BPC
296	GRMZM2G122437	Metal ion transporter	TF–DEG	GRMZM2G142179	ERF
297	GRMZM2G122437	Metal ion transporter	TF–DEG	GRMZM2G153454	bHLH
298	GRMZM2G122437	Metal ion transporter	TF–DEG	GRMZM2G164735	BBR-BPC
299	GRMZM2G123486	Heavy metal transport/detoxification superfamily protein	miRNA–DEG	zma-miR528a-3p	zma-miR528a-3p
300	GRMZM2G123486	Heavy metal transport/detoxification superfamily protein	miRNA–DEG	zma-miR528b-3p	zma-miR528b-3p
301	GRMZM2G123486	Heavy metal transport/detoxification superfamily protein	TF–DEG	GRMZM2G050939	C2H2
302	GRMZM2G123486	Heavy metal transport/detoxification superfamily protein	TF–DEG	GRMZM2G118690	BBR-BPC
303	GRMZM2G123486	Heavy metal transport/detoxification superfamily protein	TF–DEG	GRMZM2G140694	Dof
304	GRMZM2G123486	Heavy metal transport/detoxification superfamily protein	TF–DEG	GRMZM2G144744	GRAS
305	GRMZM2G123486	Heavy metal transport/detoxification superfamily protein	TF–DEG	GRMZM2G164735	BBR-BPC

306	GRMZM2G123486	Heavy metal transport/detoxification superfamily protein	TF–DEG	GRMZM2G171365	MIKC_MADS
307	GRMZM2G126860	Vacuolar sorting protein 4b	miRNA–DEG	zma-miR2275a-5p	zma-miR2275a-5p
308	GRMZM2G126860	Vacuolar sorting protein 4b	miRNA–DEG	zma-miR2275d-5p	zma-miR2275d-5p
309	GRMZM2G126860	Vacuolar sorting protein 4b	TF–DEG	GRMZM2G017087	TALE
310	GRMZM2G126860	Vacuolar sorting protein 4b	TF–DEG	GRMZM2G044576	GATA
311	GRMZM2G126860	Vacuolar sorting protein 4b	TF–DEG	GRMZM2G164735	BBR-BPC
312	GRMZM2G126860	Vacuolar sorting protein 4b	TF–DEG	GRMZM2G171365	MIKC_MADS
313	GRMZM2G126860	Vacuolar sorting protein 4b	TF–DEG	GRMZM2G477238	G2-like
314	GRMZM2G128995	Vacuolar proton-transporting V-type ATPase, V1 domain	miRNA–DEG	zma-miR166a-5p	zma-miR166a-5p
315	GRMZM2G128995	Vacuolar proton-transporting V-type ATPase, V1 domain	miRNA–DEG	zma-miR166c-5p	zma-miR166c-5p
316	GRMZM2G128995	Vacuolar proton-transporting V-type ATPase, V1 domain	miRNA–DEG	zma-miR166m-5p	zma-miR166m-5p
317	GRMZM2G128995	Vacuolar proton-transporting V-type ATPase, V1 domain	miRNA–DEG	zma-miR167g-3p	zma-miR167g-3p
318	GRMZM2G128995	Vacuolar proton-transporting V-type ATPase, V1 domain	TF–DEG	AC198403.3_FG001	HSF
319	GRMZM2G128995	Vacuolar proton-transporting V-type ATPase, V1 domain	TF–DEG	GRMZM2G003944	TCP
320	GRMZM2G128995	Vacuolar proton-transporting V-type ATPase, V1 domain	TF–DEG	GRMZM2G010100	ERF
321	GRMZM2G128995	Vacuolar proton-transporting V-type ATPase, V1 domain	TF–DEG	GRMZM2G015281	ERF
322	GRMZM2G128995	Vacuolar proton-transporting V-type ATPase, V1 domain	TF–DEG	GRMZM2G018398	ERF
323	GRMZM2G128995	Vacuolar proton-transporting V-type ATPase, V1 domain	TF–DEG	GRMZM2G021790	ERF
324	GRMZM2G128995	Vacuolar proton-transporting V-type ATPase, V1 domain	TF–DEG	GRMZM2G023708	ERF
325	GRMZM2G128995	Vacuolar proton-transporting V-type ATPase, V1 domain	TF–DEG	GRMZM2G053298	Nin-like
326	GRMZM2G128995	Vacuolar proton-transporting V-type ATPase, V1 domain	TF–DEG	GRMZM2G057386	ERF
327	GRMZM2G128995	Vacuolar proton-transporting V-type ATPase, V1 domain	TF–DEG	GRMZM2G060517	ERF
328	GRMZM2G128995	Vacuolar proton-transporting V-type ATPase, V1 domain	TF–DEG	GRMZM2G066158	ERF
329	GRMZM2G128995	Vacuolar proton-transporting V-type ATPase, V1 domain	TF–DEG	GRMZM2G079653	ERF
330	GRMZM2G128995	Vacuolar proton-transporting V-type ATPase, V1 domain	TF–DEG	GRMZM2G092214	TCP
331	GRMZM2G128995	Vacuolar proton-transporting V-type ATPase, V1 domain	TF–DEG	GRMZM2G107031	TCP
332	GRMZM2G128995	Vacuolar proton-transporting V-type ATPase, V1 domain	TF–DEG	GRMZM2G111415	ERF
333	GRMZM2G128995	Vacuolar proton-transporting V-type ATPase, V1 domain	TF–DEG	GRMZM2G125777	NAC

334	GRMZM2G128995	Vacuolar proton-transporting V-type ATPase, V1 domain	TF–DEG	GRMZM2G142179	ERF
335	GRMZM2G128995	Vacuolar proton-transporting V-type ATPase, V1 domain	TF–DEG	GRMZM2G142751	TCP
336	GRMZM2G128995	Vacuolar proton-transporting V-type ATPase, V1 domain	TF–DEG	GRMZM2G307119	ERF
337	GRMZM2G128995	Vacuolar proton-transporting V-type ATPase, V1 domain	TF–DEG	GRMZM2G310368	ERF
338	GRMZM2G128995	Vacuolar proton-transporting V-type ATPase, V1 domain	TF–DEG	GRMZM2G376255	ERF
339	GRMZM2G128995	Vacuolar proton-transporting V-type ATPase, V1 domain	TF–DEG	GRMZM2G381441	ERF
340	GRMZM2G128995	Vacuolar proton-transporting V-type ATPase, V1 domain	TF–DEG	GRMZM2G445944	TCP
341	GRMZM2G131473	Methionine aminopeptidase	miRNA–DEG	zma-miR171l-3p	zma-miR171l-3p
342	GRMZM2G131473	Methionine aminopeptidase	miRNA–DEG	zma-miR171m-3p	zma-miR171m-3p
343	GRMZM2G131473	Methionine aminopeptidase	miRNA–DEG	zma-miR171a-3p	zma-miR171a-3p
344	GRMZM2G131473	Methionine aminopeptidase	miRNA–DEG	zma-miR171b-3p	zma-miR171b-3p
345	GRMZM2G131473	Methionine aminopeptidase	miRNA–DEG	zma-miR171c-3p	zma-miR171c-3p
346	GRMZM2G131473	Methionine aminopeptidase	miRNA–DEG	zma-miR171f-3p	zma-miR171f-3p
347	GRMZM2G131473	Methionine aminopeptidase	miRNA–DEG	zma-miR171n-3p	zma-miR171n-3p
348	GRMZM2G131473	Methionine aminopeptidase	TF–DEG	GRMZM2G015281	ERF
349	GRMZM2G131473	Methionine aminopeptidase	TF–DEG	GRMZM2G021790	ERF
350	GRMZM2G131473	Methionine aminopeptidase	TF–DEG	GRMZM2G023708	ERF
351	GRMZM2G131473	Methionine aminopeptidase	TF–DEG	GRMZM2G038291	C2H2
352	GRMZM2G131473	Methionine aminopeptidase	TF–DEG	GRMZM2G050939	C2H2
353	GRMZM2G131473	Methionine aminopeptidase	TF–DEG	GRMZM2G057386	ERF
354	GRMZM2G131473	Methionine aminopeptidase	TF–DEG	GRMZM2G079653	ERF
355	GRMZM2G131473	Methionine aminopeptidase	TF–DEG	GRMZM2G111415	ERF
356	GRMZM2G131473	Methionine aminopeptidase	TF–DEG	GRMZM2G140033	C2H2
357	GRMZM2G131473	Methionine aminopeptidase	TF–DEG	GRMZM2G140694	Dof
358	GRMZM2G131473	Methionine aminopeptidase	TF–DEG	GRMZM2G142179	ERF
359	GRMZM2G131473	Methionine aminopeptidase	TF–DEG	GRMZM2G144744	GRAS
360	GRMZM2G131473	Methionine aminopeptidase	TF–DEG	GRMZM2G164735	BBR-BPC
361	GRMZM2G131473	Methionine aminopeptidase	TF–DEG	GRMZM2G171365	MIKC_MADS

362	GRMZM2G131473	Methionine aminopeptidase	TF–DEG	GRMZM2G307119	ERF
363	GRMZM2G131473	Methionine aminopeptidase	TF–DEG	GRMZM2G320287	C2H2
364	GRMZM2G131473	Methionine aminopeptidase	TF–DEG	GRMZM2G376255	ERF
365	GRMZM2G142870	ABC transporter C family member 14	TF–DEG	GRMZM2G023708	ERF
366	GRMZM2G142870	ABC transporter C family member 14	TF–DEG	GRMZM2G079653	ERF
367	GRMZM2G142870	ABC transporter C family member 14	TF–DEG	GRMZM2G111415	ERF
368	GRMZM2G142870	ABC transporter C family member 14	TF–DEG	GRMZM2G112548	NAC
369	GRMZM2G142870	ABC transporter C family member 14	TF–DEG	GRMZM2G307119	ERF
370	GRMZM2G142870	ABC transporter C family member 14	TF–DEG	GRMZM5G862109	AP2
371	GRMZM2G148800	Oligopeptide transmembrane transporter	miRNA–DEG	zma-miR159f-5p	zma-miR159f-5p
372	GRMZM2G148800	Oligopeptide transmembrane transporter	TF–DEG	GRMZM2G017087	TALE
373	GRMZM2G148800	Oligopeptide transmembrane transporter	TF–DEG	GRMZM2G023708	ERF
374	GRMZM2G148800	Oligopeptide transmembrane transporter	TF–DEG	GRMZM2G118690	BBR-BPC
375	GRMZM2G148800	Oligopeptide transmembrane transporter	TF–DEG	GRMZM2G144744	GRAS
376	GRMZM2G148800	Oligopeptide transmembrane transporter	TF–DEG	GRMZM2G164735	BBR-BPC
377	GRMZM2G148800	Oligopeptide transmembrane transporter	TF–DEG	GRMZM2G171365	MIKC_MADS
378	GRMZM2G156599	Yellow stripe 1	miRNA–DEG	zma-miR395k-3p	zma-miR395k-3p
379	GRMZM2G156599	Yellow stripe 1	miRNA–DEG	zma-miR164e-5p	zma-miR164e-5p
380	GRMZM2G156599	Yellow stripe 1	miRNA–DEG	zma-miR167e-3p	zma-miR167e-3p
381	GRMZM2G156599	Yellow stripe 1	miRNA–DEG	zma-miR167f-3p	zma-miR167f-3p
382	GRMZM2G156599	Yellow stripe 1	miRNA–DEG	zma-miR169l-3p	zma-miR169l-3p
383	GRMZM2G156599	Yellow stripe 1	miRNA–DEG	zma-miR164a-5p	zma-miR164a-5p
384	GRMZM2G156599	Yellow stripe 1	miRNA–DEG	zma-miR164b-5p	zma-miR164b-5p
385	GRMZM2G156599	Yellow stripe 1	miRNA–DEG	zma-miR164c-5p	zma-miR164c-5p
386	GRMZM2G156599	Yellow stripe 1	miRNA–DEG	zma-miR164d-5p	zma-miR164d-5p
387	GRMZM2G156599	Yellow stripe 1	miRNA–DEG	zma-miR164f-5p	zma-miR164f-5p
388	GRMZM2G156599	Yellow stripe 1	miRNA–DEG	zma-miR164g-5p	zma-miR164g-5p
389	GRMZM2G156599	Yellow stripe 1	miRNA–DEG	zma-miR164h-5p	zma-miR164h-5p

390	GRMZM2G156599	Yellow stripe 1	miRNA–DEG	zma-miR395a-3p	zma-miR395a-3p
391	GRMZM2G156599	Yellow stripe 1	miRNA–DEG	zma-miR395b-3p	zma-miR395b-3p
392	GRMZM2G156599	Yellow stripe 1	miRNA–DEG	zma-miR395d-3p	zma-miR395d-3p
393	GRMZM2G156599	Yellow stripe 1	miRNA–DEG	zma-miR395e-3p	zma-miR395e-3p
394	GRMZM2G156599	Yellow stripe 1	miRNA–DEG	zma-miR395f-3p	zma-miR395f-3p
395	GRMZM2G156599	Yellow stripe 1	miRNA–DEG	zma-miR395g-3p	zma-miR395g-3p
396	GRMZM2G156599	Yellow stripe 1	miRNA–DEG	zma-miR395h-3p	zma-miR395h-3p
397	GRMZM2G156599	Yellow stripe 1	miRNA–DEG	zma-miR395i-3p	zma-miR395i-3p
398	GRMZM2G156599	Yellow stripe 1	miRNA–DEG	zma-miR395j-3p	zma-miR395j-3p
399	GRMZM2G156599	Yellow stripe 1	miRNA–DEG	zma-miR395n-3p	zma-miR395n-3p
400	GRMZM2G156599	Yellow stripe 1	miRNA–DEG	zma-miR395p-3p	zma-miR395p-3p
401	GRMZM2G156599	Yellow stripe 1	TF–DEG	AC187157.4_FG005	HD-ZIP
402	GRMZM2G156599	Yellow stripe 1	TF–DEG	GRMZM2G014653	NAC
403	GRMZM2G156599	Yellow stripe 1	TF–DEG	GRMZM2G100593	NAC
404	GRMZM2G156599	Yellow stripe 1	TF–DEG	GRMZM2G104246	CPP
405	GRMZM2G156599	Yellow stripe 1	TF–DEG	GRMZM5G813651	NAC
406	GRMZM2G156599	Yellow stripe 1	TF–DEG	GRMZM5G870592	MYB
407	GRMZM2G157263	Ferric-chelate reductase	miRNA–DEG	zma-miR164c-3p	zma-miR164c-3p
408	GRMZM2G157263	Ferric-chelate reductase	miRNA–DEG	zma-miR164h-3p	zma-miR164h-3p
409	GRMZM2G157263	Ferric-chelate reductase	TF–DEG	AC207656.3_FG002	ARF
410	GRMZM2G157263	Ferric-chelate reductase	TF–DEG	GRMZM2G003514	MIKC_MADS
411	GRMZM2G157263	Ferric-chelate reductase	TF–DEG	GRMZM2G017087	TALE
412	GRMZM2G157263	Ferric-chelate reductase	TF–DEG	GRMZM2G018398	ERF
413	GRMZM2G157263	Ferric-chelate reductase	TF–DEG	GRMZM2G060517	ERF
414	GRMZM2G157263	Ferric-chelate reductase	TF–DEG	GRMZM2G062244	HD-ZIP
415	GRMZM2G157263	Ferric-chelate reductase	TF–DEG	GRMZM2G153754	CPP
416	GRMZM2G157263	Ferric-chelate reductase	TF–DEG	GRMZM2G159547	MYB
417	GRMZM2G157263	Ferric-chelate reductase	TF–DEG	GRMZM2G172327	MYB

418	GRMZM2G157263	Ferric-chelate reductase	TF–DEG	GRMZM2G320287	C2H2
419	GRMZM2G157263	Ferric-chelate reductase	TF–DEG	GRMZM2G359952	MIKC_MADS
420	GRMZM2G178190	Metal ion transmembrane transporter activity	miRNA–DEG	zma-miR171h-5p	zma-miR171h-5p
421	GRMZM2G178190	Metal ion transmembrane transporter activity	miRNA–DEG	zma-miR171k-5p	zma-miR171k-5p
422	GRMZM2G178190	Metal ion transmembrane transporter activity	miRNA–DEG	zma-miR827-5p	zma-miR827-5p
423	GRMZM2G421857	Vacuolar proton pump 3	miRNA–DEG	zma-miR167a-3p	zma-miR167a-3p
424	GRMZM2G421857	Vacuolar proton pump 3	miRNA–DEG	zma-miR395f-5p	zma-miR395f-5p
425	GRMZM2G421857	Vacuolar proton pump 3	miRNA–DEG	zma-miR169m-3p	zma-miR169m-3p
426	GRMZM2G421857	Vacuolar proton pump 3	miRNA–DEG	zma-miR396f-3p	zma-miR396f-3p
427	GRMZM2G421857	Vacuolar proton pump 3	TF–DEG	GRMZM2G015281	ERF
428	GRMZM2G421857	Vacuolar proton pump 3	TF–DEG	GRMZM2G021790	ERF
429	GRMZM2G421857	Vacuolar proton pump 3	TF–DEG	GRMZM2G057386	ERF
430	GRMZM2G421857	Vacuolar proton pump 3	TF–DEG	GRMZM2G079653	ERF
431	GRMZM2G421857	Vacuolar proton pump 3	TF–DEG	GRMZM2G142179	ERF
432	GRMZM2G421857	Vacuolar proton pump 3	TF–DEG	GRMZM2G144744	GRAS
433	GRMZM2G421857	Vacuolar proton pump 3	TF–DEG	GRMZM2G376255	ERF
434	GRMZM5G827496	NRT1/PTR family 3.1	miRNA–DEG	zma-miR159h-3p	zma-miR159h-3p
435	GRMZM5G827496	NRT1/PTR family 3.1	miRNA–DEG	zma-miR159i-3p	zma-miR159i-3p
436	GRMZM5G827496	NRT1/PTR family 3.1	miRNA–DEG	zma-miR159g-3p	zma-miR159g-3p
437	GRMZM5G827496	NRT1/PTR family 3.1	miRNA–DEG	zma-miR408a	zma-miR408a
438	GRMZM5G827496	NRT1/PTR family 3.1	miRNA–DEG	zma-miR408b-3p	zma-miR408b-3p
439	GRMZM5G827496	NRT1/PTR family 3.1	TF–DEG	GRMZM2G017470	Dof
440	GRMZM5G827496	NRT1/PTR family 3.1	TF–DEG	GRMZM2G033570	EIL
441	GRMZM5G827496	NRT1/PTR family 3.1	TF–DEG	GRMZM2G087804	G2-like
442	GRMZM5G827496	NRT1/PTR family 3.1	TF–DEG	GRMZM2G093725	Dof
443	GRMZM5G827496	NRT1/PTR family 3.1	TF–DEG	GRMZM2G110153	MIKC_MADS
444	GRMZM5G827496	NRT1/PTR family 3.1	TF–DEG	GRMZM2G140694	Dof
445	GRMZM5G827496	NRT1/PTR family 3.1	TF–DEG	GRMZM2G144744	GRAS

446	GRMZM5G827496	NRT1/PTR family 3.1	TF–DEG	GRMZM2G162481	WOX
447	GRMZM5G843141	ATP synthase subunit alpha (atp1-a2)	TF–DEG	GRMZM2G050939	C2H2
448	GRMZM5G843141	ATP synthase subunit alpha (atp1-a2)	TF–DEG	GRMZM2G095904	MYB
449	GRMZM5G843141	ATP synthase subunit alpha (atp1-a2)	TF–DEG	GRMZM2G162481	WOX
450	GRMZM5G843141	ATP synthase subunit alpha (atp1-a2)	TF–DEG	GRMZM2G179885	NAC
451	GRMZM5G862882	Cation transmembrane transporter	miRNA–DEG	zma-miR171d-5p	zma-miR171d-5p
452	GRMZM5G862882	Cation transmembrane transporter	miRNA–DEG	zma-miR171e-5p	zma-miR171e-5p
453	GRMZM5G862882	Cation transmembrane transporter	TF–DEG	AC216247.3_FG001	HSF
454	GRMZM5G862882	Cation transmembrane transporter	TF–DEG	GRMZM2G171365	MIKC_MADS

Table S4. The GRN features of the transporter and mugineic acid pathway genes.

Target		Interactions		
		miRNA–DEG	TF–DEG	Total
GRMZM2G014914	Plasma membrane intrinsic protein 2	4	23	27
GRMZM2G015295	Adenosylhomocysteinase	4	22	26
GRMZM2G015401	Mitochondrial phosphate transporter	10	21	31
GRMZM2G015955	Zinc transporter 4	0	21	21
GRMZM2G027098	Tonoplast intrinsic protein 2	12	19	31
GRMZM2G036908	Cation transmembrane transporter	16	15	31
GRMZM2G050108	Nicotianamine synthase 3	6	15	21
GRMZM2G054123	S-adenosylmethionine synthetase 1	4	14	18
GRMZM2G056908	Tonoplast intrinsic protein 2	2	12	14
GRMZM2G064023	Citrate Synthase 2	4	11	15
GRMZM2G064382	ZRT-IRT-like protein 5	2	11	13
GRMZM2G067546	Vacuolar sorting receptor homolog 1	4	9	13
GRMZM2G069198	NRAMP transporter1	5	8	13
GRMZM2G070360	V-type proton ATPase subunit E3	2	8	10
GRMZM2G070605	S-adenosylmethionine decarboxylase proenzyme	2	8	10
GRMZM2G092125	Plasma membrane intrinsic protein 2	1	8	9
GRMZM2G094497	Vacuolar ATP synthase subunit B	4	7	11
GRMZM2G099340	Metallothionein-like protein type 2	23	6	29
GRMZM2G099628	Probable methionine-tRNA ligase	6	6	12
GRMZM2G104418	Proton-transporting V-type ATPase, V0 domain	2	6	8
GRMZM2G122437	Metal ion transporter	1	6	7
GRMZM2G123486	Heavy metal transport/detoxification superfamily protein	0	6	6
GRMZM2G126860	Vacuolar sorting protein 4b	3	5	8
GRMZM2G128995	Vacuolar proton-transporting V-type ATPase, V1 domain	2	5	7
GRMZM2G131473	Methionine aminopeptidase	5	4	9

GRMZM2G142870	ABC transporter C family member 14	1	4	5
GRMZM2G148800	Oligopeptide transmembrane transporter	7	3	10
GRMZM2G156599	Yellow stripe 1	4	3	7
GRMZM2G157263	Ferric-chelate reductase	4	2	6
GRMZM2G178190	Metal ion transmembrane transporter activity	2	2	4
GRMZM2G421857	Vacuolar proton pump 3	1	1	2
GRMZM5G827496	NRT1/ PTR family 3.1	4	0	4
GRMZM5G843141	ATP synthase subunit alpha (atp1-a2)	3	0	3
GRMZM5G862882	Cation transmembrane transporter	1	0	1

Table S5. Primer sequences of DEGs selected from microarray analyses for qRT-PCR validation.

S. No.	Probe ID	Gene Model	Primer	Primer Sequence	Length	T_m	GC%
1	Zm.12619.1.A1_at	GRMZM2G069198	F	GACATCTCCAGCATGCAACT	20	62	50.00
			R	AGGATGAACAGAAGCCAATACC	22	62	45.46
2	Zm.13452.2.A1_at	GRMZM2G015955	F	ACAACACCGGCAGGAATAG	19	62	52.63
			R	AGCAGATACCGAGTCAAGAATG	22	62	45.46
3	Zm.582.1.S1_at	GRMZM2G156599	F	AGTGAGATGGCATGTGTAGTTG	22	62	45.46
			R	CTGCCTCGATCAAGAGAAGATG	22	62	50.00
4	Zm.614.1.A1_at	GRMZM2G056908	F	GTTCGTCTGGATTCAGCTCAT	21	62	47.62
			R	CCAGGACGACACACATCATT	20	62	50.00

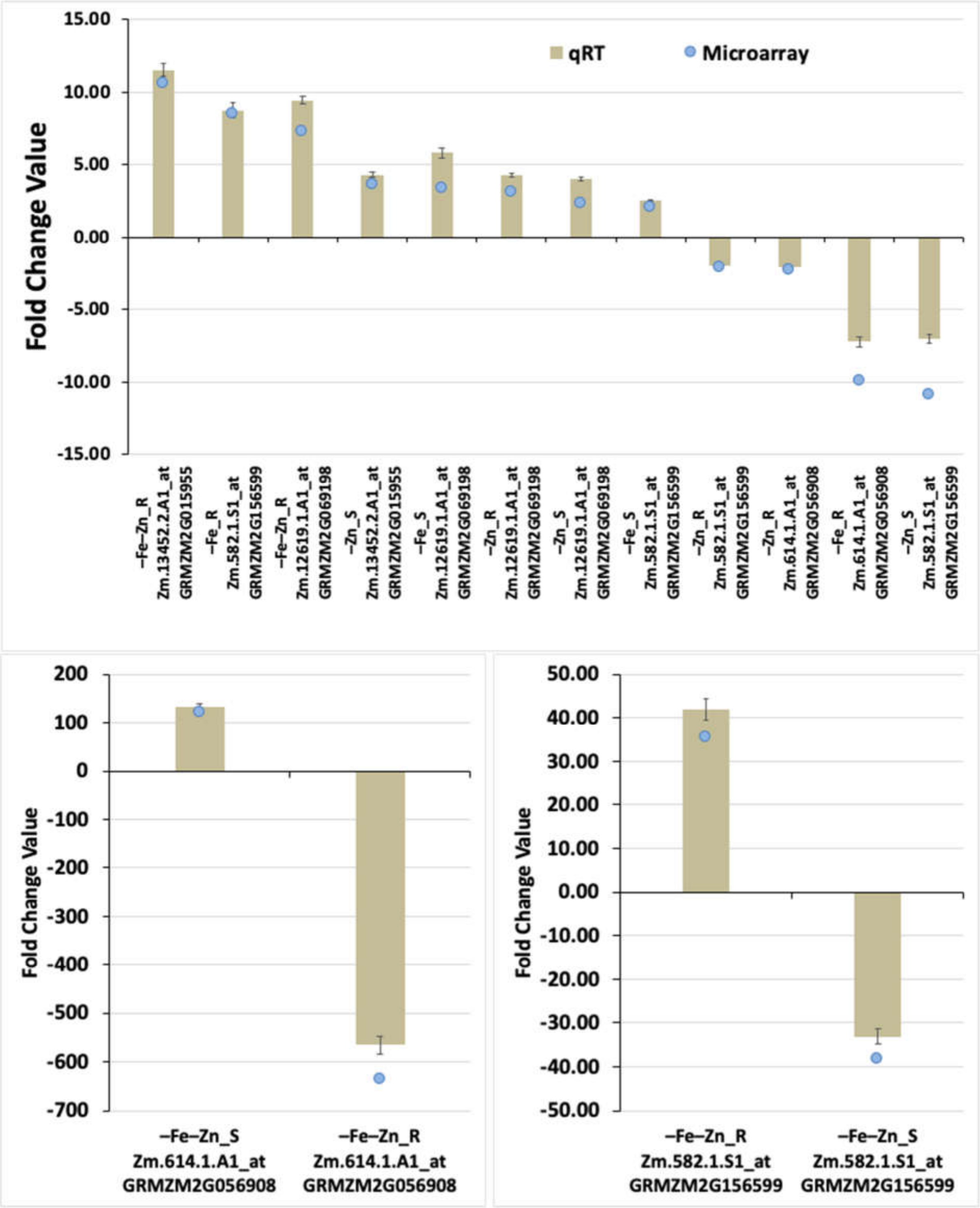

Figure S6. Validation of DEGs from microarray analysis through qRT-PCR. The x axis represents the probes and the y axis represents the fold change expression values of genes. Error bars in the column represent the standard error. The letters 'S' and 'R' in the stress name on the x axis refer to the shoot and root, respectively. The reactions were performed using the Stratagene MX3005P (Agilent Technologies, Santa Clara, California, USA) Real-Time PCR system with the following PCR conditions: 10 min, at 95 °C (preheating), followed by 40 cycles of amplification with denaturation for 30 s at 60 °C, primer annealing for 1 min at 58 °C and primer extension for 30 s at 72 °C.
